# Does variety in hedonic spending improve happiness? Testing alternative causal mechanisms between hedonic variety and subjective well-being

**DOI:** 10.1186/s40359-024-01599-8

**Published:** 2024-02-26

**Authors:** Joe J. Gladstone, Peter M. Ruberton, Seth Margolis, Sonja Lyubomirsky

**Affiliations:** 1 Leeds School of Business, University of Colorado Boulder, Boulder, Colorado United States of America; 2 Department of Psychology, The Pennsylvania State University, State College, Pennsylvania United States of America; 3 Department of Psychology, University of California Riverside, Riverside, California United States of America

**Keywords:** Hedonic adaption, Subjective well-being, Economic psychology, Spending, Consumer behavior

## Abstract

Previous research has found only a small, inconsistent association between hedonic consumption and subjective well-being, often attributed to individuals adapting to the happiness gains from their purchases. Given that diverse experiences can reduce or avert hedonic adaptation, we hypothesized that variety in hedonic spending would be associated with greater well-being. This hypothesis was tested in four studies (total *N* = 2,920), using both self-reported and objective bank-reported spending data. In our correlational analyses, hedonic spending variety was uniquely associated with well-being, even after controlling for total hedonic spending and other financial variables. Our investigation also explored the directional relationship between hedonic spending variety and well-being, yielding mixed results for both causal pathways in two time-lagged panel studies. Additionally, in two parallel experiments, participants reported that varied hedonic spending contributed more to happiness than uniform hedonic spending. These findings have implications for basic well-being science by testing how varied consumption behaviors and well-being are interrelated.

**Supplementary Information:**

The online version contains supplementary material available at 10.1186/s40359-024-01599-8.

## Introduction

Consider two friends, Mark and Maria, who differ in how they spend their money. Specifically, Mark spends most of his discretionary funds on a few pleasurable areas (e.g., sports equipment and music), whereas Maria purchases in a wide variety of hedonic domains (e.g., cinema trips, coffee shops, tourism, and video games), but spends much less on pleasurable goods than Mark does overall. Based on their respective shopping habits, which friend would be expected to be the happier of the two?

The dilemma of Mark and Maria illustrates a central finding in research on wealth and well-being—namely, that money’s contribution to happiness is contingent on how that money is spent [1]. One important distinction is the difference between hedonic and utilitarian consumption. Hedonic products are multisensory, pleasant, and fun; they are enjoyable and appeal to your senses [2, 3]. Examples include cut flowers, video games, and designer shoes. Utilitarian products are primarily instrumental, and their purchase is motivated by the extent to which they are useful, practical, or functional, helping individuals to achieve their goals [2, 3]. Examples include insurance products, home cleaning materials, and medicine. Compared with utilitarian goods, hedonic goods are generally considered more discretionary [4], and they are linked to positive feelings [5]. However, the happiness gains from a single hedonic purchase are short-lived [6, 7], suggesting that hedonic consumption alone is an inefficient path to greater happiness.

Furthermore, although most studies to date have focused on spending as a cause of well-being, a growing body of research suggests that the inverse may be true as well: People’s affective states may influence their desire and engagement with hedonic purchases, including how varied their hedonic spending is. For example, when people are experimentally manipulated into feeling increased positive affect, they show an increased preference for varied consumption of foods [8, 9] and in their choice of brands [10]. Thus, variety in hedonic spending may be a consequence, rather than simply an antecedent, of greater subjective well-being.

To our knowledge, no research has directly tested the link between variety in hedonic spending and well-being. If hedonic spending is related to well-being, is hedonic variety a consequence of well-being, is well-being a consequence of variety, or is there a bidirectional relationship? To address these questions, our research investigates the association between varied hedonic spending and well-being, and the causal direction(s) responsible for this association, using both subjective and objective spending data derived from correlational, longitudinal, and experimental methods.

### How variety may cause well-being

One explanation for why wealth has inconsistent benefits for well-being is hedonic adaptation; that is, the well-being gains or losses derived from different life changes tend to erode with time, such that people return to their baseline level of well-being [11]. In the context of spending behaviors, the positive boost from a recent purchase may quickly wear off due to hedonic adaptation. Like drug addicts, people may become caught up in an addictive cycle of spending and consumption, spending ever-increasing amounts of money in search of ever-declining rewards.

The Hedonic Adaptation Prevention (HAP) model [12, 13] suggests a potential path to mitigating the adaption to hedonic spending: Increase *variety* in spending, particularly variety in hedonic (rather than utilitarian) spending. The theory suggests that incorporating greater variety across spending should help counter the forces of hedonic adaptation by maintaining interest in purchases. This counter-effect occurs because people adapt more slowly to varied, surprising, and novel stimuli. Indeed, having a variety of positive experiences has been linked to greater positive emotions [14, 15].

Furthermore, research has demonstrated that buying experiences can make people happier than buying possessions, in part because experiences involve relatively greater social connection [16]. Therefore, varied hedonic spending may also “buy” a high amount of social interaction, to the extent that variety in hedonic spending reflects relatively high experiential spending. Experiences also tend to be more varied than possessions, and experiences characterized by relatively greater variety are associated with greater well-being [17, 18]. For example, while traveling overseas on holiday, a person will often engage in many novel activities, such as eating different foods, visiting unusual attractions, and meeting new people. By contrast, material purchases, such as new furniture, are not typically surprising or dynamic. After a person acclimates to a new living room set, there remains little novelty or excitement to be gained from the purchase, and the object no longer engenders positive emotions. Therefore, one reason that experiential purchases (e.g., vacations) are associated with greater happiness may be that they provide a variety of positive experiences.

The Hedonic Adaptation Prevention model is not the only relevant theory that supports a potential causal effect of hedonic spending variety on well-being. Wilson and Gilbert also provide a theory of affective adaptation encapsulated by the acronym AREA: attend, react, explain, and adapt [19]. The AREA model attempts to explain why people adapt more quickly to some experiences rather than others. Their explanation is that sense-making plays a key role in this process—that is understanding why something happened helps people to move on and thus adapt to the positive or negative emotions associated with that experience. As a result, when a person is unable to explain an event or experience, the situation exerts a strong pull on their attention, thus inhibiting adaptation. Under this model, varied hedonic spending should “reset” the adaptation process by providing a steady stream of new stimuli (e.g., goods or experiences) to attract attention and, in turn, elicit positive emotions.

### How well-being may cause variety

Variety in hedonic spending may also reflect the habits of inherently happier people. The broaden-and-build theory [20, 21] suggests that positive emotions expand people's immediate thought-action repertoires, broadening their attention, cognition, and range of actions. This theory builds on prior work showing that positive affect facilitates approach behavior [22, 23]. Sensory pleasure, for instance, motivates people to approach and continue consuming whatever stimulus is biologically useful for them at that moment [24]. According to this perspective, positive emotions motivate individuals to engage with their environments and partake in a wider range of activities. By contrast, negative emotions are linked with specific action tendencies (e.g., fear with the urge to escape) that narrow perceived choices to promote quick and decisive action. Therefore, the characteristic thought-action patterns linked with positive emotions—such as exploring, savoring, integrating, or envisioning future achievements—could promote diverse hedonic spending.

Economists can also point to a contrary relationship between varied hedonic spending and well-being. Classical economic theory assumes that consumers’ spending choices are optimized to maximize their well-being [25]. Therefore, any difference in spending variety among consumers could be explained by individual preferences, implying that well-being should be independent of spending on a more diverse set of goods. According to this theory, interventions that aim to increase spending variety may even harm well-being by deviating from the optimal distribution of resources. However, this perspective contradicts a growing body of evidence from behavioral economics and psychology that shows that people often do not solve optimization problems perfectly [26]. Besides the significant cognitive effort required, individuals often mispredict what will make them happy [27, 28], indicating that a more refined understanding of the relationship between spending variety and well-being is needed.

### Research questions and hypotheses

The main goal of the present studies is to examine how hedonic spending variety affects happiness. We suggest the following hypotheses. First, we expect a positive relationship between hedonic spending variety and well-being (Hypothesis 1A). Second, we hypothesize that this correlation will still hold after controlling for potential covariates that may explain the hedonic variety-happiness link (Hypothesis 1B). Specifically, we propose that the amount of variety in hedonic spending will be associated with greater well-being above and beyond overall financial health (i.e., income, liquid wealth, investments, and debt) and total spending on hedonic goods. In other words, we expect that the correlation between hedonic variety and well-being is not merely an artifact of high levels of financial well-being and hedonic consumption. Moreover, spending variety alone should not be related to well-being; only variety in spending for fun or enjoyment (i.e., hedonic spending) should be associated with greater happiness, because only an assortment of *positive* experiences should maximize the hedonic impact of spending. By contrast, we expect that variety in utilitarian spending—spending on functional goods, such as basic life necessities—will not be associated with well-being, because utilitarian purchases do not inherently bring positive emotions. Indeed, diverse utilitarian spending may signal turmoil in a person’s life (e.g., changes in life circumstances, such as moving or divorce).

We also sought to test the causal links between hedonic spending variety and well-being using longitudinal methods. Our evidence includes participants who reported their spending behaviors and well-being at two time points separated by five months. Hypothesis 2 tests between competing causal paths using regressed change models: first, that hedonic spending variety predicts changes in well-being (Hypothesis 2A), and second, that well-being predicts changes in hedonic spending variety, above and beyond initial variety (Hypothesis 2B). Although our research specifically aimed to test the effect of hedonic variety on well-being, our longitudinal data allow us to test the time-lagged association—a compelling indicator of causality—between hedonic variety and happiness in both directions.

We tested our hypotheses across three studies (see Table 1 for a summary). Study 1, which tested Hypotheses 1A and 1B only, used objective spending data from customers at a large bank in the United Kingdom. Studies 2 and 3 used detailed self-reported spending data to replicate and extend the findings from Study 1 by assessing participants’ spending behaviors and well-being at multiple time points, as well as by experimentally manipulating recalled spending behaviors. An additional experimental study (Study 4), drawing participants from the follow-up samples of Studies 2 and 3, further explores these questions.

**Table 1:** Outline of study samples

Study	Sample size	Spending data source	Outcomes	Design	Hypotheses tested
1	527	Bank-reported (UK)	Life satisfaction	Cross-sectional	1A/B
2	Initial: 993 Follow-up: 632	Self-reported (UK)	Positive affect, Life satisfaction	Cross-sectional + 5-month follow-up and experiment	1A/B, 2A/B
3	Initial: 1400 Follow-up: 718	Self-reported (US)	Positive affect, Life satisfaction	Cross-sectional + 5-month follow-up	1A/B, 2A/B
4	632 and 718	Self-reported (UK and US)	Perceived happiness from spending	Experiment on both follow-up samples	2A

## Study 1

### Procedures and participants

Study 1 used objective bank-reported spending data, paired with a survey measuring the life satisfaction of customers. The dataset used in Study 1 was collected by a U.K.-based multinational bank in late 2014. A randomly selected sample of customers of the bank living in the United Kingdom were recruited by email to complete a survey about their financial attitudes and behaviors, as well as their life satisfaction. Customers who completed the survey were asked to consent to having their responses matched with their personal transaction data. Of the 1,013 people who completed the survey, 912 (90%) agreed to have their survey responses matched with their account records. This approach of collecting both objective and subjective data enabled the inclusion of various covariates in our models, such as relationship status, levels of debt, investments, liquid wealth, and income, that have previously been shown to predict subjective and financial well-being [29–34].

We excluded participants who did not appear to use the bank account in this study as their primary spending account. First, we excluded participants who reported that the bank account in this study was not their primary account (remaining *N* = 617). Second, we excluded participants who did not have at least 1 spending transaction in each of the 12 months preceding the survey (remaining *N* = 595). Finally, we excluded participants who had fewer than 135 total spending transactions (i.e., 1 standard deviation below the mean, rounded up), leaving a final sample of 527 participants (54.5% female; *M*_age_ = 37.21, *SD*_age_ = 14.40). Study 1 received ethical approval from the University of Cambridge Institutional Review Board (protocol 15/018) prior to data analysis.

### Survey measures

#### Life satisfaction

Life satisfaction was assessed with the 5-item Satisfaction With Life Scale [35], a widely-used measure of global life evaluation. Each item was completed on a 5-point Likert scale, and answers to the 5 items were averaged (excluding unanswered items), yielding a possible score range of 1 to 5. The scale had good internal reliability in this sample (Cronbach’s α = .86).

#### Employment status

Self-reported employment status was dummy-coded on three variables: Employed (working full-time or part-time; *n* = 401), Student (*n* = 30), and Retired (*n* = 42). Participants who were not in any of the 1-coded groups (i.e., who were not employed, a student, or retired; *n* = 54). were scored as 0 on each variable. Additionally, 21 participants who gave an open-ended “Other” response to the employment question were categorized by one of the authors (PMR); the open-ended responses and their recoded statuses are available in the Supplemental Materials.

#### Relationship status

Participants reported their relationship status as one of three levels: married, living with a partner, or single (including widowed, divorced, or separated). Relationship status was then converted to a single dichotomous variable (In Relationship [married or living with partner] vs. No Relationship) and dummy coded with No Relationship as the 0-coded group (*n* = 222). A small number (*n* = 13) of participants did not report relationship data; these participants were coded as No Relationship.

#### Total investments

Participants reported the total value of their investments, excluding pension plans, at the time of the survey. To correct for positive skew, scores were log transformed after adding 1 to each response so that the minimum value after applying the log transformation was 0 (*M* = 0.60, median = 0, *SD* = 1.39).

#### Debt status

Participants reported their total outstanding debt from personal loans and credit cards, excluding mortgages and business loans, at the time of the survey. Because the majority of participants (*n* = 315) reported having no debt, debt status was converted to a dichotomous variable (Debt vs. No Debt) and dummy coded with No Debt as the 0-coded group.

### Bank-reported measures

#### Spending

All purchases and withdrawals made by participants using the bank account in this study were reported by the bank. Individual transactions were not reported separately; rather, a monthly summary for each unique organization (e.g., “BP Amoco,” “Amazon”), including the number of transactions and amount spent (in British pounds) at that organization each month, was reported. The organizations were grouped by the bank into one of 241 spending categories describing the transaction (e.g., “Public Transport,” “Golf,” “Menswear”). The number of transactions and amount spent in each of these categories were aggregated over the 12 months of available data for each participant. Participants completed an average of 385.89 transactions during the study period (*SD* = 183.23; minimum = 135; maximum = 1,287; median = 355), meaning our analysis was based on 202,362 transactions in total.

#### Liquid wealth

Liquid wealth was the monthly average of participants’ combined checking and savings account balances as reported by the bank on the first day of each month [32]. To correct for positive skew, scores were log transformed prior to analyses after adding a constant to each response so that the minimum value after applying the log transformation was 0 (*M* = 3.54, median = 3.41, *SD* = 0.40).

#### Income

Income was the total credits to participants’ checking accounts over the prior 12 months. To correct for positive skew, scores were log transformed prior to analyses (*M* = 4.30, median = 4.30, *SD* = 0.30).

### Classification of spending as hedonic or utilitarian

Twenty-five British adults (11 male, 14 female; *M*_age_ = 36.08 [*SD* = 12.78]) from the survey website Prolific Academic rated each of the 241 bank-reported spending categories on how hedonic and utilitarian they were (1 = *Not at all*, 9 = *Extremely*). Hedonic and utilitarian ratings were made separately, rather than on a single bipolar scale. All raters read the following definition of hedonic and utilitarian products: “Hedonic products are pleasant and fun; they are enjoyable and appeal to your senses. Utilitarian products are useful, practical, or functional; they help you achieve a goal. It is possible for a product to be equally hedonic and utilitarian. It is also possible for a product to be higher on one quality than it is on the other.” These descriptions are consistent with the definitions we used in the opening of the paper [2, 3]. They then rated how hedonic and utilitarian the spending categories were, with categories presented in a random order. The interrater reliability of these ratings, determined by their intraclass correlation coefficients (ICCs), was excellent (hedonic ICC [2, 25] = .95, utilitarian ICC [2, 25] = .93).

Based on these ratings, the spending categories were classified as either hedonic, utilitarian, both hedonic and utilitarian, or neither hedonic nor utilitarian. A category was classified as hedonic if (a) its mean hedonic rating was greater than 5 (i.e., the midpoint of the scale), (b) its mean utilitarian rating was less than 5, and (c) the mean difference between its hedonic and utilitarian ratings was significant at the *p* < .10 level using a paired *t*-test. The same process, with the hedonic and utilitarian labels swapped, was used to determine utilitarian categories. Spending categories were classified as “both” if their hedonic and utilitarian mean ratings were both above 5. Note that “both” was a stand-alone classification; that is, categories classified as “both” were not also classified as hedonic and utilitarian separately. Categories were classified as “neither” if they did not meet the criteria to be classified as hedonic, utilitarian, or both. A small number of categories that were clearly misinterpreted by the raters (as determined by inspecting the organizations within the categories), or that were classified differently than another psychologically similar category, were reclassified post-rating by the first three authors. Categories that reflected psychologically equivalent spending behaviors (e.g., “Cable & satellite TV” and “Cable TV” or different types of charities) were then combined to ensure that each category genuinely varied from the others. The above criteria produced a final list of 214 discrete spending categories (45 hedonic, 129 utilitarian, 23 both, 17 neither). See [36] for a similar dichotomization approach. The final list of spending categories and their classifications, including all combinations and reclassifications, is available in the Supplemental Materials.

### Calculating spending variety

To assess spending variety, we adapted a measure of emodiversity (i.e., emotional variety; [37]), which was derived from the Shannon biodiversity index of the evenness of species in a biological ecosystem [38, 39]. Specifically, variety was calculated for both hedonic and utilitarian spending using the formula:

$$\text{Variety} = -1 \times \sum\nolimits_{i=1}^{S} (P_{i} \times \ln P_{i})$$

where *S* is the total number of hedonic (utilitarian) categories in which an individual had any spending and *P*_*i*_ is the proportion of total spending in all hedonic (utilitarian) categories from the *i*th category. As such, to calculate hedonic spending variety, we:

1. Divided the amount an individual spent in the first hedonic category by the individual’s total hedonic spending,
2. Multiplied this proportion by its natural logarithm (*P*_*i*_ × ln *P*_*i*_), and
3. Summed these products across all *S* categories and multiplied the result by -1.

We then repeated this process for utilitarian categories to calculate utilitarian spending variety. Participants who had no hedonic spending, and thus would return an undefined value from this variety formula, were manually assigned a hedonic variety score of 0. No participants had zero utilitarian spending.

In our measure of variety resulting from this calculation, high values indicate more varied spending patterns, whereas an individual whose hedonic spending was concentrated entirely in one category would have a variety score of 0. By contrast, an individual who spent the same amount across all possible hedonic categories would have a maximally high variety score. As noted by Quoidboch and colleagues (2014), this measure of variety thus captures both the total number of distinct categories in which a participant had spending (“richness” of spending) and how balanced that participant’s spending was across all categories (“evenness” of spending) [37].

In addition to spending variety, we also calculated the total amount that participants spent across all hedonic and utilitarian categories. To correct for positive skew and account for potential diminishing returns of spending for well-being, total spending was log-transformed (base 10) before analyses.

## Results

### Zero-order correlations

As hypothesized, hedonic spending variety during the preceding 12 months was significantly correlated with life satisfaction (*r* = .12, 95% CI = [.033, .20], *p* = .007), but utilitarian spending variety was not significantly correlated with life satisfaction (*r* = .07, 95% CI = [-.016, .15], *p* = .11). However, the correlations of hedonic and utilitarian variety with life satisfaction did not differ significantly from one another, *t* = 0.90, *p* = .37. Descriptive statistics and zero-order correlations are reported in the Supplemental Materials (Tables S1 and S2).

### Hierarchical regression

To isolate the role of hedonic variety from total log-hedonic spending and overall financial health, we conducted a series of hierarchical regression models. First, demographics and financial variables were entered in preliminary models. Utilitarian variety and log utilitarian spending were entered in Model 1. Total log-hedonic spending was entered in Model 2. Finally, hedonic spending variety was entered in Model 3. Regression statistics from Model 3 for hedonic spending variety are reported in Table 2. Complete regression coefficients and model fit statistics for all models are reported in Supplemental Materials (Tables S3 and S4).

**Table 2:** Summary of regression coefficients for hedonic spending variety, hypothesis 1B (cross-sectional predicting well-being)

Outcome:	Positive Affect				Life Satisfaction			
Study	β	[95% CI]	s*r*	*p*	β	[95% CI]	s*r*	*p*
1					.11	[.008, .21]	.086	.035
2	.15	[.078, .23]	.12	<.001	.073	[-.002, .15]	.058	.055
3 (split self-report)	.085	[.021, .15]	.064	.009	.016	[-.046, .17]	.012	.61
3 (limited self-report)	.10	[.035, .17]	.074	.003	.055	[-.009, .12]	.040	.091
3 (external ratings)	.034	[-.030, .098]	.026	.30	.035	[-.026, .097]	.027	.26

β = Standardized regression coefficient. s*r* = Semipartial correlation coefficient

Denominator degrees of freedom: Study 1 = 510; Study 2 = 968; Study 3 = 1376

In Model 2 total log-hedonic spending, but not total log-utilitarian spending, was uniquely associated with greater life satisfaction. Consistent with our hypothesis, in Model 3, hedonic variety uniquely predicted life satisfaction above and beyond total log-hedonic spending and financial health (see Table 2). Secondary analyses using a sample that included participants excluded by the 135-transaction minimum (total *N* = 595) produced a nearly identical effect of hedonic spending variety in the final model, β = .11, *p* = .045. As such, excluding participants on the basis of a minimum-transaction criterion did not affect our conclusions in Study 1.

By contrast, the unique effect of utilitarian variety was slightly negative and nonsignificant, β = -.01, 95% CI = [-.10, .079], *t* = -0.24, *p* = .81. The semipartial correlation of hedonic variety and life satisfaction (*r*_semipartial_ = .086) was comparable in magnitude to the semipartial correlations of log-liquid wealth (*r*_semipartial_ = .11) and log-investments (*r*_semipartial_ = .11) with life satisfaction. Furthermore, the effect of total log-hedonic spending was reduced to nonsignificance when hedonic variety was included in the model, suggesting that the role of hedonic spending in well-being was explained by increases in spending variety concurrent with increases in raw hedonic spending.

### Moderation effect of overall wealth

Our hierarchical regression models showed that hedonic spending variety was associated with well-being independently of overall wealth and hedonic spending. However, to show more directly that hedonic variety relates to well-being independently of total wealth, we examined several models testing the interactions between hedonic variety and other measures of wealth—specifically, income, liquid wealth, and investments. All covariates were also included in the moderation models. None of the financial variables moderated the association between hedonic spending variety and life satisfaction, absolute value of all βs ≤ .03, all *p*s > .52. Additionally, even after controlling for the interactions with wealth, the main effect of hedonic variety remained significant or marginally significant (controlling for interaction with investments; *p* = .055). Thus, we found no evidence that the role of hedonic spending variety in life satisfaction was moderated by total wealth.

## Study 2

The results of Study 1 provided encouraging evidence for the importance of hedonic spending variety to well-being: Hedonic variety was modestly, but significantly, associated with greater life satisfaction, whereas utilitarian variety was not. However, Study 1 assessed only the cognitive component of subjective well-being (i.e., life satisfaction), whereas past research suggests variety might be more important for the emotional aspect of well-being—in particular, for positive moods and emotions (e.g., [14, 15, 40]).

In Study 2, we addressed these limitations using a single large self-report sample, allowing us to directly replicate the main finding of Study 1 using a larger sample and measuring both life satisfaction and positive affect as outcomes. Study 2 also allowed us to isolate the role of hedonic spending variety from two alternative variables that might explain its effect—namely, spending on high-status and experiential goods.

## Materials and methods

### Participants

Participants were 993 adults living in the United Kingdom recruited from Prolific Academic between September 27 and September 30, 2016 (59.2% female;* M*_age_ = 35.22, *SD*_age_ = 12.11). We determined our minimum sample size to provide at least 80% power between Studies 1 and 2 combined (α = .05, *ρ* = .08, based on the results from Study 1). This analysis produced a target *N* of 1,224 participants in Studies 1 and 2 combined, or 697 participants in Study 2 alone. We elected to use a pooled power analysis for Study 2 to maximize efficiency of resource use, given the large sample required. Given available resources, we set our target *N* for Study 2 at 1,000 participants, which yields approximately 72% power at α = .05 and *ρ* = .08 in Study 2 alone and approximately 88% power in Studies 1 and 2 combined. The raw data file for Study 2, Time 1 is available on the Open Science Framework (osf.io/xwpjn/?view_only=6aac277546184216a3059bfd328358a4). The research in Study 2 was approved by the Institutional Review Board at the University of California, Riverside (protocol HS-16-135). Consent was obtained electronically prior to each survey.

All 993 participants in Study 2 were recontacted through Prolific Academic to complete a follow-up survey. The follow-up survey was administered between February 21 and March 2, 2017, approximately 5 months after the initial survey. Of the 993 eligible participants, 632 (63.6%) completed the follow-up survey. This sample size was not determined in advance, but rather by the number of participants who completed the follow-up survey within 10 days of its posting. After 10 days, the survey was closed, and no further responses were collected. Participants who completed the follow-up survey reported slightly lower Time 1 well-being (life satisfaction: *t*[991] = -2.49, *p* = .013, *d* = -0.16; positive affect: *t*[991] = -2.30, *p* = .022, *d* = -0.15) and hedonic spending variety (*t*[991] = -3.07, *p* = .002, *d* = -0.20) than participants who did not complete the follow-up. The raw data file for Study 2, Time 2 is available on the Open Science Framework (osf.io/xwpjn/?view_only=6aac277546184216a3059bfd328358a4).

### Procedure

Participants were recruited online for a “Spending behaviour study.” After completing measures of life satisfaction and affect, they selected from a checklist all spending categories from which they personally made at least one purchase during the past 12 months. The spending categories closely reflected those used in Study 1, except that categories classified as “Neither” (i.e., neither hedonic nor utilitarian) were not included to reduce participant burden. Additionally, categories that were semantically similar to one another were combined (e.g., “Pensions” and “Pensioners”), even if they were separate categories in Study 1, and nonspecific categories (e.g., “Direct shopping other”) were not included. These criteria produced a final checklist of 186 categories (44 hedonic, 121 utilitarian, 21 both). To make the checklist easier to follow, categories were presented in groups with similar categories based on higher-level spending groups provided by the bank in Study 1. The full list of categories is available in the Supplemental Material.

After completing the spending category checklist, participants were presented with the categories they selected and indicated the number of purchases they made and their best estimates of the exact amount they spent in each category over the past 12 months. They also reported how much they spent as an ordinal variable using a list of predefined spending ranges (“Less than £1,” “£1 to £3,” “£4 to £10,” “£11 to £30,” “£31 to £100,” etc., up to “More than £10,000.”). Note that these ranges follow powers of 10 in approximately 0.5-log units (e.g., the range £31 to £100 is equal to £10^1.49^ to £10^2^.)

The follow-up assessment for Study 2 followed the same procedure as the initial assessment, except that participants reported their spending behaviors “since 1 October 2016” (i.e., since the end of the initial survey). Well-being and financial information measures were the same as the initial assessment, except that participants reported their positive and negative emotions over the previous five months.

### Measures

#### Demographics

Participants reported their age in years, sex, and relationship status (Married, Separated/Divorced, Widowed, Single). Participants’ employment statuses (Employed, Student, Retired, None) were reported by Prolific Academic. Sex, relationship status, and employment status were dummy coded, with Male, Single, and None as the 0-coded groups, respectively.

#### Life satisfaction

As in Study 1, life satisfaction was assessed using the Satisfaction With Life Scale [35] using a 7-point Likert scale. Answers to the 5 items were averaged (excluding unanswered items), giving a possible score range of 1 to 7. Reliability was good in this sample (Cronbach’s α = .90).

#### Positive and negative affect

Positive and negative affect were assessed using the Affect-Adjective Scale [41], which measures the extent to which participants felt four positive emotions (“Happy,” “Pleased,” “Joyful,” “Enjoyment/fun”) and five negative emotions (“Worried/anxious,” “Angry,” “Frustrated,” “Depressed/blue,” “Unhappy”). Participants reported the extent to which they felt each emotion “in the past year” using a 7-point scale from *Not at all* to *Extremely*. Answers to the positive and negative affect items were averaged separately (excluding unanswered items), providing a possible score range of 1 to 7 for each variable. Cronbach’s αs for positive and negative affect were .93 and .89, respectively.

#### Financial information

Participants reported (a) the approximate average balance of their checking and savings accounts at the end of each month over the previous year (i.e., liquid wealth); (b) their personal annual income; (c) the total value any investments they owned; and (d) the outstanding balances of their credit card and personal loan debt (excluding mortgages), if any. As in Study 1, liquid wealth, income, and investments were log-10 transformed prior to analysis and debt status was converted to a dichotomous variable (0 = no debt).

#### Spending and spending variety

Total hedonic and utilitarian spending, as well as spending variety, were calculated using the same procedures as Study 1, based on the exact spending amounts reported by participants for each category. In cases where participants did not report an exact amount for a given category, but did select a predefined spending range, the amount of spending for that category was set at the base 10 logarithmic midpoint of the chosen spending range after subtracting 1 from the low end of the range. For example, the range £300 to £1000 is equal to £10^2.48^ to £10^3^. The logarithmic midpoint of this range is therefore 10 to the power of the average of 2.48 and 3, or 10^2.74^, which is 547.72. (The range “Less than £1” was defined as £10^-0.523^ to £10^0^ = £0.30 to £1. The range “More than £10,000” was defined as £10^4^ to £10^4.478^ = £10,000 to £30,000.) Spending values were imputed in this manner for 11.7% of all selected spending categories, and the imputed values were used to calculate both total spending and spending variety.

**Fig. 1:**
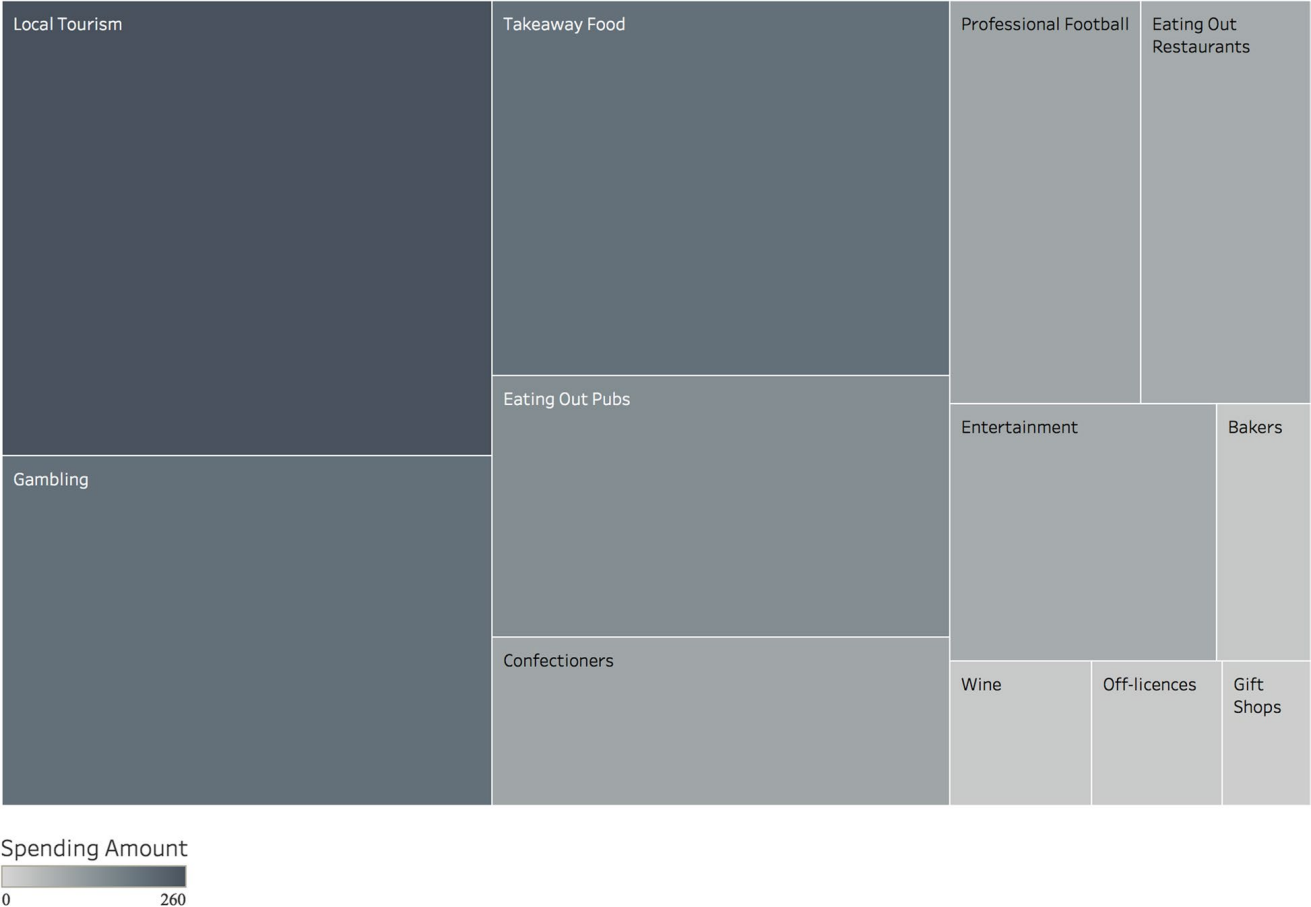
Treemap plot of a participant from Study 2 (Time 1) at the 75th percentile of hedonic spending variety.  The size and shade of the box indicates the relative amount of spending in each category

**Fig. 2:**
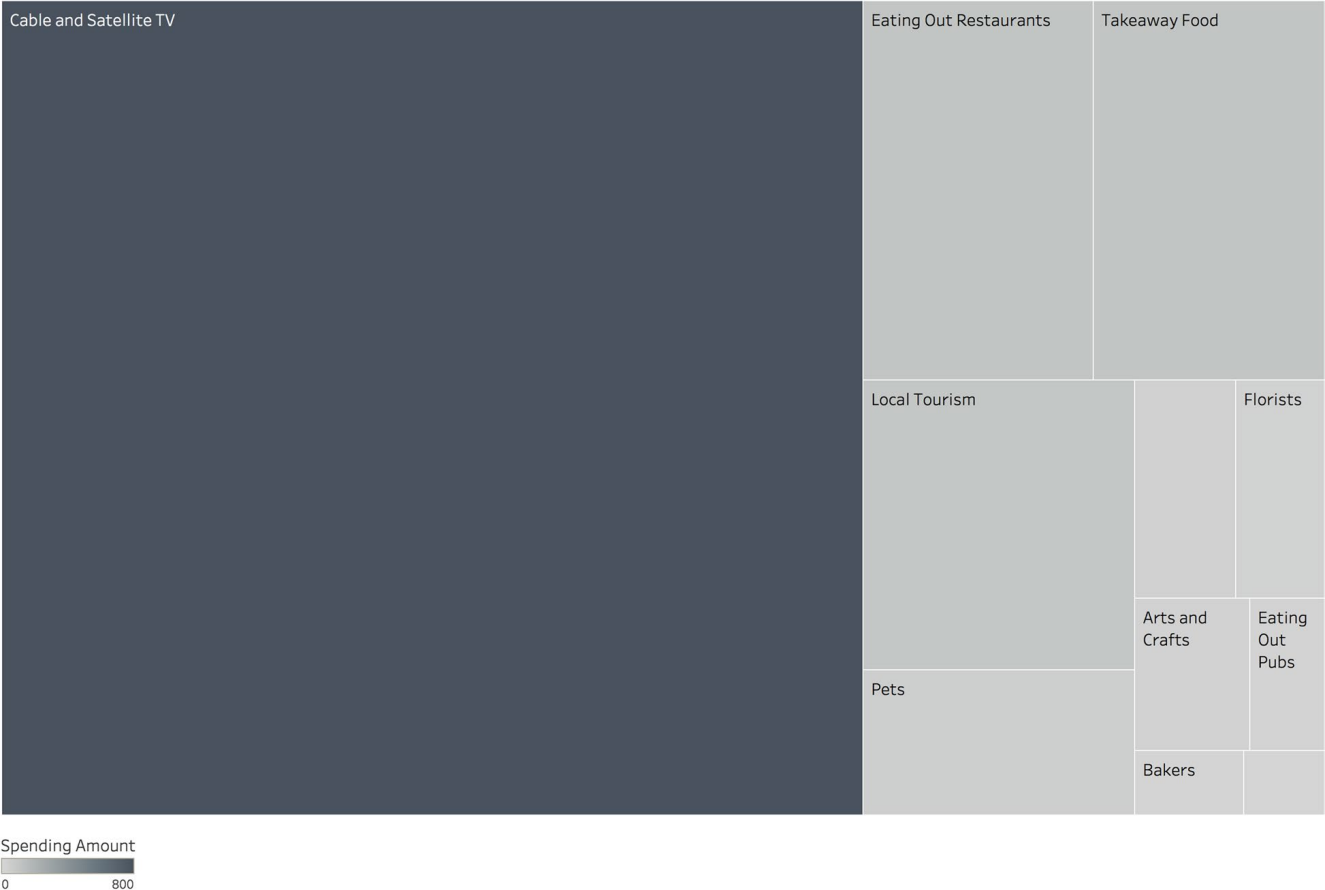
Treemap plot of a participant from Study 2 (Time 1) at the 23rd percentile of hedonic spending variety.  The size and shade of the box indicates the relative amount of spending in each category

To demonstrate our approach to measuring spending variety, we showcase "treemap" visualizations based on the hedonic spending data from two different individuals in Study 2 (Time 1), following the same variety measurement methodology as in Study 1. Both participants had identical total hedonic spending amounts (£1,229 over a 12-month period). However, there was a notable difference in their spending variety: the first participant (illustrated in Fig. 1) ranked in the 75th percentile for variety (score = 2.19), while the second participant (shown in Fig. 2) was in the 23rd percentile (score = 1.33). This contrast provides a clear comparison of high versus low spending variety, independent of the total amount spent.

In the treemap plots, each box corresponds to expenditure in a specific category by each participant. The size and color intensity of these boxes visually represent the relative spending amounts: larger and darker boxes indicate higher spending in that category compared to smaller, lighter boxes. For instance, Fig. 1 displays a participant with high spending variety, indicated by relatively balanced spending across numerous hedonic categories, such as domestic UK tourism, gambling, and takeaway and pub food. In contrast, Fig. 2 shows a participant with low spending variety, where a significant portion of their hedonic spending is concentrated on cable/satellite television, with fewer categories represented. This visual representation helps to clearly differentiate between participants with high and low levels of spending variety.

### Experiential and status spending classifications

An alternative explanation for our proposed effect is that the role of spending variety could instead be driven by spending that is more experiential (vs. material) in nature, or by spending on high-status goods and services. Specifically, if hedonic spending that is varied is also highly experiential or high-status, then any associations between variety and well-being may be due to experiential or status spending influencing subjective well-being, instead of hedonic variety.

In addition to the hedonic and utilitarian category ratings, we recruited a new sample of independent raters (*N* = 36 adults from Prolific Academic; *M*_age_ = 34.78) to rate the spending categories, in a random order, on their perceived social status (high status vs. low status), as well as whether the spending category was mostly material or experiential. Social status spending was defined as: “A high-status spending category increases the social prestige of its owner. A low-status spending category decreases the social prestige of its owner. Some fall in the middle and are seen as neither high nor low in status.” Material/experiential spending was defined as: “Material spending is spending on tangible goods, something you can hold in your hand and is long-lasting. Experiential spending is spending on experiences, where you cannot hold this physically and are left only with memories after the experience.”

Ratings on each dimension were made on 1 (*Very low status* and *Completely material*) to 7 (*Very high status* and *Completely experiential*) scales, and ratings were averaged across all raters to calculate the status and experientialism scores for each category. A category was classified as high-status or experiential if its status or experientialism score was at least 1 standard deviation above the respective mean score across all categories. High-status spending categories included “Sailing,” “Jewelry,” and “Antiques.” Experiential spending categories included “Massage parlors,” “Bowling,” and “Dancing.” Total spending in the high-status and experiential categories, separately, was then calculated for each participant. Procedures and analyses for experiential and status spending classifications were preregistered on the Open Science Framework (osf.io/sxcve/?view_only=551470806103495bb8c2d38434646e3d).

## Results

### Zero-order correlations

As hypothesized, hedonic spending variety during the preceding 12 months was significantly correlated with both life satisfaction (*r* = .12, 95% CI = [.061, .18], *p* < .001) and positive affect (*r* = .20, 95% CI = [.14, .26], *p* < .001). By contrast, utilitarian variety was significantly correlated with life satisfaction (*r* = .08, 95% CI = [.014, .14], *p* = .016), but positive affect did not reach significance at the 5% level (*r* = .055, 95% CI = [-.007, .12], *p* = .081). The correlation between hedonic variety and positive affect was significantly stronger than the correlation between utilitarian variety and positive affect, *t* = 4.16, *p* < .001; however, the correlations of hedonic and utilitarian variety with life satisfaction did not differ significantly from one another, *t* = 1.33, *p* = .18. Descriptive statistics and zero-order correlations for Study 2, Time 1 are reported in Supplemental Materials (Tables S5 and S6).

### Hierarchical regression

We conducted a series of hierarchical regression models predicting both positive affect and life satisfaction using the same model building procedure as in Study 1. Regression statistics for hedonic spending variety for both outcomes are reported in Table 3. Complete regression coefficients and model fit statistics for all models for both outcomes are reported in Supplemental Materials (Tables S7 through S10).

**Table 3:** Summary of regression coefficients for hedonic spending variety, hypothesis 2A (time-lagged predicting well-being)

Outcome:	Positive Affect				Life Satisfaction			
Study	β	[95% CI]	s*r*	*p*	β	[95% CI]	s*r*	*p*
2	.080	[.010, .15]	.059	.025	.069	[.013, .13]	.052	.016
3 (split self-report)	.047	[-.039, .085]	.017	.47	.015	[-.032, .063]	.011	.52
3 (limited self-report)	.002	[-.059, .064]	.002	.94	.025	[-.021, .071]	.019	.29
3 (external ratings)	.017	[-.043, .078]	.013	.58	.040	[-.006, .086]	.030	.090

β = Standardized regression coefficient. s*r* = Semipartial correlation coefficient

Denominator degrees of freedom: Study 2 = 608; Study 3 = 695

#### Positive affect

Consistent with our hypothesis, as shown in Table 2, hedonic variety uniquely predicted positive affect above and beyond total log-hedonic spending and financial health in Model 3; by contrast, the unique effect of utilitarian variety was slightly negative and nonsignificant, β = -.06, 95% CI = [-.14, .011], *t* = -1.00, *p* = .092. The semipartial correlation of hedonic variety and positive affect (*r*_semipartial_ = .12) was comparable in magnitude to the semipartial correlation of marriage with positive affect (*r*_semipartial_ = .13).

#### Life satisfaction

As shown in Table 2, in Model 3, the unique association of hedonic variety with life satisfaction was in the hypothesized direction and marginally significant using a two-tailed test; by contrast, the unique effect of utilitarian variety was slightly negative and nonsignificant β = -.04, 95% CI = [-.11, .093], *t* = -1.00, *p* = .32. The semipartial correlation of hedonic variety and life satisfaction (*r*_semipartial_ = .058) was comparable in magnitude to the semipartial correlations of log-liquid wealth (*r*_semipartial_ = .061) and age (*r*_semipartial_ = -.061) with life satisfaction. In contrast with the results from Study 1, total log-hedonic spending retained a marginal association with life satisfaction when controlling for hedonic variety, β = .08, 95% CI = [-.004, .17], *t* = 1.86, *p* = .063. However, the residual effect of total log-hedonic spending was weakened slightly (β = .11 vs. .08) when hedonic variety was included (Model 3) compared with when hedonic variety was not included (Model 2).

### Alternative explanations: status and experiential spending

To test whether hedonic spending variety predicts well-being above and beyond high-status and experiential spending, we first calculated semipartial correlations between varied hedonic spending and well-being, controlling for the amount of high-status spending and the extent of experiential spending, with no other covariates. The semipartial correlations were .11 and .18 for life satisfaction and positive affect, respectively, only slightly below the zero-order correlations between varied hedonic spending and well-being.

We also computed semipartial correlations between varied hedonic spending and well-being after controlling for all covariates in the final hierarchical regression models in addition to high-status and experiential spending. The semipartial correlations were .06 and .12 for life satisfaction and positive affect, respectively. These semipartial correlations are almost identical to the semipartial correlations that did not control for high-status and experiential spending (*r* = .06 and *r* = .12 for life satisfaction and positive affect, respectively). Taken together, these findings indicate that the association between varied hedonic spending and well-being was not merely an artifact of high-status or experiential spending.

### Moderation effect of overall wealth

As in Study 1, we tested the moderating role of total wealth on the association between hedonic spending variety and well-being. With all covariates included, no significant interactions emerged between hedonic spending variety and log income or log investments predicting either life satisfaction or positive affect. However, there was a modest interaction between hedonic variety and log liquid wealth predicting both outcomes (life satisfaction: β = .089, *p* = .010; positive affect: β = .083, *p* = .018), such that varied hedonic spending had a relatively stronger relationship with well-being for wealthier individuals. The simple slopes of hedonic variety for participants with liquid wealth 1 standard deviation below the mean were nonsignificant (life satisfaction β = -.012, *p* = .827; positive affect β = .074, *p* = .20), indicating that varied hedonic spending did not predict well-being for participants with less money in their checking and savings accounts.

### Time 2 zero-order correlations

Hedonic spending variety during the 5-month follow-up period was significantly correlated with both life satisfaction (*r* = .10, 95% CI = [.026, .18], *p* = .008) and positive affect (*r* = .15, 95% CI = [.07, .22], *p* < .001) at the follow-up assessment. By contrast, utilitarian variety was not significantly correlated with life satisfaction (*r* = .003, 95% CI = [-.07, .08], *p* = .93) or positive affect (*r* = .039, 95% CI = [-.039, .12], *p* = .33) at follow-up. The correlations between hedonic variety and well-being were significantly stronger than the correlations between utilitarian variety and well-being for both life satisfaction (*t* = 2.50, *p* = .013) and positive affect (*t* = 2.73, *p* = .006). Descriptive statistics and zero-order correlations for Study 2, Time 2 are reported in Supplemental Materials (Tables S21 and S22, respectively).

### Well-being-as-outcome regressed change models

To assess whether hedonic variety predicted changes in well-being during the follow-up period, we conducted a series of hierarchical regression models predicting Time 2 life satisfaction and positive affect from Time 2 spending variety. Models were built using the same procedures as for Hypothesis 1B, with the additional covariate of Time 1 life satisfaction or positive affect in all models; an additional preliminary model assessing the role of Time 1 well-being only was also examined for each outcome. Demographic variables were measured at Time 1, and financial and spending variables (including variety) were measured at Time 2. Regression statistics for hedonic spending variety for both outcomes are reported in Table 3. Complete regression statistics from all models are reported in Supplemental Materials (Tables S23 through S26).

#### Positive affect

Time 1 positive affect explained most of the variance in Time 2 positive affect in Model 0 (*R*^2^ = .55). However, consistent with our hypothesis, in Model 5, hedonic variety uniquely predicted Time 2 positive affect above and beyond Time 1 positive affect, total log-hedonic spending, and financial health (see Table 3). By contrast, the unique effect of utilitarian variety was slightly negative and marginally significant, β = -.055, 95% CI = [-.12, .010], *t* = -1.65, *p* = .099. The semipartial correlation of hedonic variety and follow-up positive affect (*r*_semipartial_ = .059) was comparable in magnitude to the semipartial correlation of marriage with follow-up positive affect (*r*_semipartial_ = .057). Furthermore, the effect of total log-hedonic spending was nonsignificant in both Models 4 and 5: Hedonic spending variety, but not total amount of hedonic spending, predicted changes in positive affect.

#### Life satisfaction

Life satisfaction demonstrated greater stability over time than positive affect, with Time 1 life satisfaction explaining most of the variance in Time 2 life satisfaction in Model 0, *R*^2^ = .71. However, consistent with Hypothesis 2A, hedonic variety uniquely predicted Time 2 life satisfaction above and beyond Time 1 life satisfaction, total log-hedonic spending, and financial health (see Table 3). By contrast, the unique effect of utilitarian variety was slightly negative and nonsignificant, β = -.04, 95% CI = [-.092, .016], *t* = -1.39, *p* = .17. The semipartial correlation of hedonic variety and follow-up life satisfaction (*r*_semipartial_ = .052) was comparable in magnitude to the semipartial correlation of marriage with follow-up life satisfaction (*r*_semipartial_ = .064). Furthermore, the effect of total log-hedonic spending was nonsignificant in both Models 4 and 5: Hedonic spending variety, but not total amount of hedonic spending, predicted changes in life satisfaction.

### Variety-as-outcome regressed change models

To parse the direction of the link between hedonic variety and well-being, we tested an additional regression model predicting Time 2 hedonic variety from Time 2 life satisfaction and positive affect, controlling for Time 1 hedonic variety, with all covariates included. As shown in Table 4, neither Time 2 life satisfaction nor Time 2 positive affect significantly predicted Time 2 hedonic spending variety above and beyond Time 1 hedonic variety, suggesting that in Study 2, hedonic variety promoted higher well-being over time, but not vice-versa.

**Table 4:** Summary of regression coefficients for well-being, hypothesis 2b (time-lagged predicting hedonic spending variety)

Predictor:	Positive Affect				Life Satisfaction			
Study	β	[95% CI]	s*r*	*p*	β	[95% CI]	s*r*	*p*
2	.041	[-.016, .098]	.039	.16	.039	[-.019, .097]	.036	.19
3 (split self-report)	.012	[-.032, .080]	.022	.39	.030	[-.029, .090]	.026	.31
3 (limited self-report)	.032	[-.028, .092]	.029	.30	.033	[-.031, .096]	.028	.32
3 (external ratings)	.038	[-.019, .094]	.035	.19	.031	[-.029, .091]	.027	.32

β = Standardized regression coefficient. s*r* = Semipartial correlation coefficient

Denominator degrees of freedom: Study 2 = 608; Study 3 = 697

### Fully cross-lagged models

No significant cross-lagged effects emerged for either Time 1 hedonic spending variety predicting Time 2 well-being (life satisfaction: β = .027, *p* = .21; positive affect: β = .029, *p* = .29) or for Time 1 well-being predicting Time 2 hedonic spending variety (positive affect: β = .006, *p* = .85; life satisfaction: β = -.001, *p* =.97).

## Study 3

We sought to test whether our findings from the first two studies would replicate using a pre-registered sample of participants. Study 3 retested the cross-sectional findings from Studies 1 and 2 using a larger, U.S.-based sample of participants, with the addition of a participant-specific measure of hedonic (vs. utilitarian) spending, as well as retesting the longitudinal findings by including a follow-up survey of participants 5-months later. The methods, analyses, and hypotheses for Study 3 were pre-registered on the Open Science Framework prior to data collection (pre-registration link for Hypotheses 2A and 2B: osf.io/g6r3w/?view_only=a6136d7d80644a60a87c7cf49c4f9c85).

## Materials and method

### Participants

Participants were 1400 adults living in the United States recruited from Prolific Academic between November 15th and 25th of 2017 (50.6% female;* M*_age_ = 32.92, *SD*_age_ = 11.61). A total of 1400 individuals completed the Study 3 survey.

We determined the sample size for Study 3 using a power analysis based on the effect found in Study 2. We calculated that we needed a sample of 1338 to provide 80% power, pooling across Studies 2 and 3 (i.e., meta-analytically), to detect the effect of hedonic variety on life satisfaction (α = .80, ρ = .05). We therefore aimed to collect a sample of 1400 at Time 1. The research in Study 3 was approved by the Institutional Review Board at the University of California, Riverside (protocol HS-16-135).

All participants in Study 3 were also re-contacted through Prolific Academic to complete a follow-up survey. Of the 1400 eligible participants, 718 (51.3%) completed the follow-up survey. The follow-up survey was administered between April 14 and April 24 of 2018, approximately 5 months after the initial survey. Compared to participants who did not complete the follow-up, participants in the Time 2 survey reported lower Time 1 life satisfaction (*t*[1398] = -4.16, *p* < .001, *d* = -0.22) and positive affect (*t*[1398] = -4.92, *p* < .001, *d* = -0.26), as well as marginally lower hedonic spending variety (*t*[1398] = -1.94, *p* = .053, *d* = -0.10). The raw data file for Study 3, Times 1 and 2, are available on the Open Science Framework (osf.io/5vxne/?view_only=3d769a4338a84346b9299d4afd4d88dc).

### Procedure

The procedure for Study 3 was the same as for Study 2, with several modifications. First, to reduce participant burden, the initial spending category checklist questionnaire was omitted. Instead, participants were presented with the spending details questions (i.e., number of purchases and amount spent) for all possible categories and were asked to report about only those categories in which they made any purchases in the past 12 months. Second, for each category in which participants reported spending, they also indicated whether their purchases in that category were “mostly for pleasure and enjoyment” (i.e., hedonic), “mostly for functionality and practical uses” (i.e., utilitarian), or “a roughly equal mix of functionality and pleasure.” This change allowed us to determine whether spending was identified as hedonic or utilitarian on a participant-by-participant basis, rather than relying solely on third-party ratings as was done in Studies 1 and 2.

The list of spending categories was nearly identical to those used in Study 2, with adjustments to make the categories relevant for a U.S.-based sample. For example, the category “Solicitor Fees” was amended to “Attorney Fees,” and “U.K. Tourism” was amended to “US Tourism.” The full list of categories is available in Supplemental Materials.

The follow-up assessment for Sample 3 followed the same procedure as the first assessment, except that participants reported their spending behaviors “since November 19th, 2017” (i.e., since the end of the baseline survey). Well-being and financial information measures were the same as in the first assessment, except that participants reported their positive and negative emotions over the previous 5 months instead of the previous 12 months.

### Measures

Demographic, positive and negative affect, and life satisfaction measures were the same as in Study 2, except that the affect scale included three new items reflecting relatively low-arousal affect (“Peaceful/serene,” “Relaxed/calm,” and “Dull/bored”). Although we did not pre-register a hypothesized difference in the effects of hedonic variety for high- and low-arousal positive affect, exploratory analyses revealed that the pattern of effects we report held only for the original high-arousal positive affect items. Therefore, to simplify comparisons with Study 2, we report only effects for high-arousal positive affect in Study 3, with the caveat that these findings may not generalize to lower-arousal positive emotions. Furthermore, as in Study 2, we neither expected nor found a consequential association between hedonic spending variety and negative affect (see pre-registration). Finally, the relationship status demographic question included the new option, “In a relationship (unmarried),” which was treated as a separate dummy variable in regression models.

### Spending and spending variety

Total hedonic and utilitarian spending and spending variety were calculated in the same manner as in Studies 1 and 2. To extend upon our earlier analysis, the hedonic/utilitarian classifications were also calculated in three different ways in the new sample. First, hedonic and utilitarian classifications were determined using the same external ratings used in Studies 1 and 2 (henceforth the “externally-rated” approach). Second, hedonic and utilitarian classifications were determined using participants’ own ratings of whether their spending in each category was primarily hedonic or utilitarian, excluding categories that participants reported as equally hedonic and utilitarian (henceforth the “limited self-report” approach). This second approach thus mirrors the method used in Studies 1 and 2, in which categories that were not clearly rated as hedonic or utilitarian were excluded. Third, hedonic/utilitarian classifications were again determined using participants’ own ratings, but spending in the equally hedonic and utilitarian categories was allocated as both hedonic and utilitarian. To avoid double-counting spending, the total amount of spending in such categories was divided in half before calculating each variable; in other words, half of the spending in a “both” category was treated as hedonic, and half was treated as utilitarian (henceforth the “split self-report” approach). Total (log-transformed) hedonic and utilitarian spending, and spending variety, were calculated using each set of hedonic/utilitarian classifications.

We did not hypothesize any differences in results between these approaches to classifying spending. However, as noted in our pre-registration, the third approach, which incorporates all categories in which participants reported having spending and provides a relatively “weighted” measure of hedonic vs. utilitarian ratings, likely has the strongest ecological validity. For brevity, we therefore discuss results only for the split self-reported and externally-rated versions of the spending categories, except where the split self-report and limited self-report measures produced divergent results.

## Results

### Zero-order correlations

As hypothesized, both the split self-report and externally-rated measures of hedonic spending variety during the preceding 12 months correlated significantly with both life satisfaction and positive affect. In contrast with the findings from Studies 1 and 2, utilitarian variety was also significantly correlated with both life satisfaction and positive affect. For both outcomes, the correlations with utilitarian variety were weaker than with hedonic variety using the self-reported measures of hedonic/utilitarian spending, although the difference was not significant for life satisfaction. However, using the externally-rated measure, the utilitarian variety correlation was slightly, but nonsignificantly, stronger than the hedonic variety correlation for both life satisfaction and positive affect. Descriptive statistics and zero-order correlations for Study 3 are reported in Supplemental Materials (Tables S11 and S12, respectively).

### Hierarchical regression

We conducted a series of hierarchical regression models predicting both life satisfaction and positive affect using the same model building procedure as in Studies 1 and 2. Regression statistics for hedonic spending variety for both outcomes and all spending measurement approaches are reported in Table 2. Complete regression coefficients and model fit statistics for all regression models are reported in Supplemental Materials (Tables S13 through S20).

#### Positive affect

As hypothesized, in Model 3, hedonic variety uniquely predicted positive affect using the split self-report measure of hedonic/utilitarian spending (see Table 2), whereas the unique effect of utilitarian variety was nonsignificant. Contrary to our hypotheses, however, the final unique effect of total hedonic spending remained significant using the split self-report measures of spending, β = .15, 95% CI = [.13, .38], *t* = 4.00, *p* < .001. The semipartial correlation of hedonic variety and positive affect in the split self-report model (*r*_semipartial_ = .06) was similar to the equivalent correlation in Study 2 and comparable in magnitude to the semipartial correlation of investments with positive affect (*r*_semipartial_ = .07).

In contrast with the results from Studies 1 and 2, no significant effect of hedonic spending variety was found in the final model when using externally-rated hedonic/utilitarian spending, although the unique effect of utilitarian variety was unexpectedly strong and positive (β = .10, 95% CI = [.07, .33], *t* = 3.02, *p* = .003).

#### Life satisfaction

As shown in Table 2, in Model 3, the unique association of hedonic variety with life satisfaction was not significant using the split self-report or externally-rated measures. However, the effect was in the hypothesized direction and marginally significant using the limited self-report measures of hedonic/utilitarian spending. The unique effect of utilitarian variety was similar in magnitude to hedonic variety, but nonsignificant, using the self-report approaches, but was positive and significant using the externally-rated approach, β = .10, 95% CI = [.088, .36], *t* = 3.25, *p* = .001. Finally, total hedonic spending predicted life satisfaction using the split self-report approach, β = .10, 95% CI = [.064, .33], *t* = 2.90, *p* =.004, but not the limited self-report or externally-rated approaches.

### Moderation of overall wealth

The unique association of hedonic spending variety with life satisfaction and positive affect was not moderated by any indicators of wealth, regardless of how hedonic spending was assessed, although the interaction between limited self-report variety and log liquid wealth predicting positive affect was marginally significant (β = .042, *p* = .097).

### Internal meta-analysis of positive affect and life satisfaction correlations

To assess the unique role of hedonic spending variety in life satisfaction across all three studies, we meta-analytically combined the semipartial correlations between hedonic variety and life satisfaction from the final models. Only the correlations using the split self-report measures of hedonic/utilitarian spending in Study 3 were considered. Using a fixed-effects model with inverse-variance weighting, the meta-analytic correlation between hedonic variety and life satisfaction was small but significant (*r*_meta-analytic_ = .04, 95% CI = [.003, .08], *p* = .04). We repeated this meta-analytic approach for positive affect in Studies 2 and 3. In a fixed-effects model with inverse-variance weighting, the meta-analytic correlation was significant (*r*_meta-analytic_ = .09, 95% CI = [.05, .13], *p* < .001) and comparable to the meta-analytic correlation between total log-hedonic spending and positive affect (*r*_meta-analytic_ = .08, 95% CI = .035, .11], *p* < .001).

Taken together, the results from across the three studies provide broadly supportive evidence that hedonic spending variety is correlated with well-being (Hypothesis 1A). However, our findings for Hypothesis 1B were more mixed. Although a unique association between hedonic variety and well-being emerged in all three studies, the results from Study 3 using the externally-rated spending classifications did not replicate the pattern of results from the first two samples. In Studies 1 and 2, hedonic spending variety predicted well-being whereas utilitarian spending variety did not. However, contrary to our expectation that only hedonic variety would be linked with greater well-being, Study 3 showed evidence for an effect of both hedonic and utilitarian variety. Additionally, the regression models from Study 3 maintained a significant, positive association between total hedonic spending and well-being even after incorporating hedonic spending variety into the models. This residual effect suggests hedonic purchases may be linked to greater well-being regardless of how varied those purchases are. Despite these qualifications, our results revealed a consistent correlation between hedonic spending variety and both life satisfaction and positive affect, above and beyond other indicators of financial health.

## Time-lagged correlations between hedonic spending variety and well-being

We followed up with participants after a 5-month lag to test if variety predicts changes in well-being or if well-being predicts changes in hedonic spending variety (or both). Our theoretical basis for predicting a causal link between hedonic spending variety and well-being implicitly treats each hedonic spending category as its own “source” of well-being to which people will adapt over time. A person with highly varied hedonic spending habits should therefore experience less overall adaptation, and thus greater well-being, than a person with relatively stable spending habits, because each novel type of hedonic spending offsets hedonic adaptation to other sources. In other words, each spending-based happiness source “resets” the total amount of adaptation to hedonic purchases, allowing the purchases to bring about relatively more well-being [42]. Correspondingly, when spending variety decreases, overall well-being should decrease concurrently because of the loss of the adaptation-resetting effects that varied purchases provide. As such, our temporal models assume that variety can only impact *proximal* well-being, rather than *future* well-being. For example, people with low variety in the 5-month follow-up period should report relatively low well-being over those 5 months, even if their variety over the prior 12 months was high: If variety decreases, the hedonic benefits of variety should correspondingly decrease as well, with no “carry-over” benefits of the high initial variety. To that end, our models do not show, or attempt to show, that Time 1 variety *prospectively* predicts increases in well-being, but rather that hedonic spending variety *over a given time period* predicts relative increases in well-being over the same time period (but not vice-versa). Following this line of reasoning, we used regressed change models predicting Time 2 well-being from Time 2 predictors, controlling for Time 1 well-being.

Although our theory focuses on the proximal influence of variety on well-being, and these were the models we pre-registered, for comparison purposes we also briefly report results from conventional cross-lagged models predicting Time 2 well-being from Time 1 spending variety and, simultaneously, vice-versa.

### Time 2 zero-order correlations

As hypothesized, hedonic spending variety during the 5-month follow-up period was significantly correlated with both life satisfaction and positive affect at the follow-up assessment using all three measures of hedonic/utilitarian spending. Follow-up utilitarian variety was also significantly correlated with life satisfaction, albeit more weakly than hedonic variety, but was only correlated with positive affect using the externally-rated hedonic/utilitarian measure, and not the two self-report measures. Using the split self-report measures of variety, the correlations between Time 2 hedonic variety and well-being were stronger than the correlations between utilitarian variety and well-being for both life satisfaction (*t* = 1.84, *p* = .066) and positive affect (*t* = 2.62, *p* = .009). Descriptive statistics and zero-order correlations for Study 3, Time 2 are reported in Supplemental Materials (Tables S27 and S28, respectively).

### Well-being-as-outcome regressed change models

To assess whether hedonic variety predicted changes in well-being during the follow-up period, we conducted a series of hierarchical regression models predicting Time 2 positive affect and life satisfaction. The model building procedure was the same as in Study 2. Regression statistics for hedonic spending variety for both outcomes and all spending measures are reported in Table 3. Complete regression statistics for all models are reported in Supplemental Materials (Tables S29 through S36).

#### Positive affect

Time 1 positive affect explained most of the variance in Time 2 positive affect in Model 0, *R*^2^ = .60. However, contrary to our hypothesis, hedonic variety did not uniquely predict Time 2 positive affect above and beyond Time 1 positive affect and the other covariates using any measures of hedonic/utilitarian spending, all βs ≤ .03 (see Table 3), although all three effects were in the hypothesized direction. By contrast, the unique effects of utilitarian variety (β = -.10, *p* < .001) and total hedonic spending (β = .07, *p* = .044) were significant using both the split self-report approach.

#### Life satisfaction

Time 1 life satisfaction explained most of the variance in Time 2 life satisfaction in Model 0, *R*^2^ = .76. Paralleling the findings for positive affect, hedonic variety did not uniquely predict Time 2 life satisfaction in the final models using any measures of hedonic/utilitarian spending (see Table 3), although all three effects were positive and the effect using the externally-rated measures was marginally significant. By contrast, in the models using split self-report measures, both utilitarian variety (β = -.05, 95% CI = [-.23, -.017], *t* = 2.27, *p* = .024) and total hedonic spending (β = .08, 95% CI = [.049, .25], *t* = 2.94, *p* = .003) were significantly associated with life satisfaction.

### Variety-as-outcome regressed change models

As with Study 2, we tested an additional regression model predicting Time 2 hedonic variety from Time 2 life satisfaction and positive affect, controlling for Time 1 hedonic variety, with all covariates included. As shown in Table 4, neither Time 2 life satisfaction nor Time 2 positive affect significantly predicted Time 2 hedonic spending variety above and beyond Time 1 hedonic variety using any of the measures of hedonic (vs. utilitarian) spending.

### Fully cross-lagged models

As in Study 2, there were no significant cross-lagged effects for Time 1 hedonic spending variety predicting Time 2 life satisfaction or positive affect using any of the hedonic spending measures. Contrasting with Study 2, a small but significant cross-lagged path predicting Time 2 hedonic spending variety from Time 1 positive affect emerged using the split self-reported measures of hedonic spending (β = .077, *p* = .016), but not using the externally-rated measure (β = .045, *p* = .13). A similar pattern emerged in models predicting Time 2 variety from Time 1 life satisfaction (split self-report: β = .10, *p* = .001; externally-rated: β = .051, *p* = .082).

## Study 4

The outcomes of Studies 1-3 consistently indicated a positive relationship between the variety of hedonic spending and enhanced well-being. However, due to the correlational nature of these studies, they do not definitively establish causation, especially in light of mixed results regarding Hypotheses 2A and 2B. To further explore the causal dimension, we conducted an experimental study aimed at examining whether varied hedonic spending yields greater perceived happiness compared to uniform spending. In this experiment, participants were presented with lists of spending categories—either varied or uniform—based on their past purchases or hypothetical spending scenarios. They then assessed how their spending in these categories contributed to their happiness. This experimental approach draws parallels to previous studies that investigated the effects of spending on experiential versus material goods on well-being [17] and those comparing the impact of spending time versus money [43]). While acknowledging that the act of spending on varied goods and experiences is not identical to retrospectively evaluating their hedonic impact, this experimental setup provides a valuable test of the potential causal relationship between hedonic spending variety and well-being. By randomly assigning participants to varied or uniform spending categories, we aimed to isolate the effect of variety on perceived happiness, thereby adding a critical experimental dimension to our exploration of this relationship.

## Materials and methods

### Participants

The experimental study involved participants who had already completed follow-up surveys in Studies 2 and 3. Participants engaged in one of two distinct experiments. The first focused on the effects of their actual spending, and the second on the impact of hypothetical spending scenarios. Both experiments were structured using a 2x2 factorial design, examining the effects of two variables: type of spending (hedonic vs. utilitarian) and variety of spending (varied vs. uniform). The distribution of participants across each condition and experiment is detailed in Table 5 (top).

**Table 5:** Sample Size Per Condition in the Real and Hypothetical Spending Experiments, Study 2 Sample (top) and Study 3 Sample (bottom)

Variety of spending	Experiment			
	Real		Hypothetical	
	Hedonic	Utilitarian	Hedonic	Utilitarian
Variety (7 categories)	*n* = 104	*n* = 109	*n* = 52	*n* = 56
Uniformity (1 category)	*n* = 111	*n* = 102	*n* = 54	*n* = 44
Variety (7 categories)	*n* = 69	*n* = 73	*n* = 110	*n* = 106
Uniformity (1 category)	*n* = 66	*n* = 68	*n* = 127	*n* = 101

For the real spending experiment, participants qualified if their Time 1 spending data met specific criteria: (a) at least one purchase in seven or more different hedonic categories, (b) at least one purchase in seven or more utilitarian categories, (c) a minimum of seven purchases in one or more hedonic categories, and (d) a similar pattern in one or more utilitarian categories. These criteria were aligned with the different conditions of the real spending experiment.

Participants whose spending patterns did not satisfy all four criteria—for example, those who did not make purchases in at least seven different hedonic categories at Time 1—were ineligible for random assignment to all conditions in the real spending experiment. Instead, these participants were included in the hypothetical spending experiment.

This dual-experiment approach allowed us to comprehensively investigate the impact of hedonic spending variety. It enabled the inclusion of all participants, not just those with extensive spending data, thereby facilitating a broader examination of the effects of actual and hypothetical spending behaviors on well-being.

Participants in the study were presented with either a diverse selection of seven spending categories (variety condition) or a single spending category (uniformity condition). The categories were exclusively hedonic or utilitarian. In the real spending experiment, participants were shown categories in which they reported at least one purchase at Time 1. Specifically, those in the variety condition were shown seven categories randomly chosen from those where they had made at least one purchase. In contrast, those in the uniformity condition were shown one category, randomly selected, where they had made at least seven purchases. Participants were then asked to recall the specific items they purchased in these categories over the past year. For those in the hypothetical spending experiment, the process involved categories in which they hadn't made any purchases at Time 1, and they were asked to imagine what they might buy in these categories.

Participants then answered four questions about these purchases, adapted from prior research [17]. Two questions focused on the hedonic impact of the purchases (“Taken as a whole, how much do these purchases contribute to your happiness in life?” and “When you think about these purchases, how happy do they make you?”), while the other two dealt with the perceived value of the purchases (“To what extent do you think the money spent on these purchases would have been better spent on some other purchase(s) that would have made you happier?” and “Overall, to what extent would you say that these purchases were money well-spent?”). In the hypothetical experiment, these questions were posed as if the purchases were potential future expenditures. Responses were recorded on a 9-point scale, ranging from 0 (*Not at all*) to 8 (*Extremely*).

An exploratory factor analysis with oblimin rotation on the responses from the real spending experiment indicated two distinct factors: happiness contribution and perceived value. However, as our primary interest lies in the hedonic impact of spending variety, not the perceived value, our analysis focused solely on the happiness contribution questions. These were combined into a single composite score for further analysis.

The participants in the Study 3 sample followed the same procedures as the Study 2 sample, with one modification: In the real spending experiment, spending categories were classified as hedonic or utilitarian based on participants’ self-reported hedonic/utilitarian classifications (omitting split hedonic/utilitarian categories) instead of the externally-rated classifications used in Study 2. The sample from Study 3 served as a direct replication of the one from Study 2. The experimental procedures, hypotheses, and analyses for the Study 3 sample were pre-registered on the Open Science Framework (available at osf.io/cm7tg/?view_only=0d35063a89db4e6480b1e5588ea7807f).

## Results

### Real spending (Study 2 sample)

**Fig. 3:**
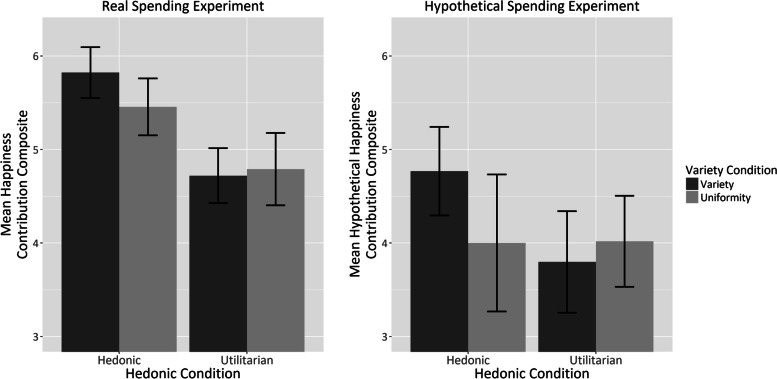
Mean happiness contribution of purchases across hedonic/utilitarian and variety/uniformity conditions in the Study 2 sample experiments. Real experiment: left side, N = 424; hypothetical experiment: right side, N = 204. Error bars are 95% confidence intervals. Happiness contribution scores ranged from 0 to 8

For the real spending experiment, a 2 (hedonic vs. utilitarian) by 2 (variety vs. uniformity) factorial ANOVA was calculated to examine differences in how much each type of spending contributed to participants’ happiness. As illustrated in Fig. 3 (left), a significant main effect emerged for hedonic spending, such that participants who considered purchases in hedonic categories reported that those purchases contributed more to their happiness than participants who considered purchases in utilitarian categories, *F*(1, 422) = 30.44, *p* < .01, η_partial_ = .26. There was no main effect of considering varied purchases over uniform purchases, *F*(1, 422) = 0.87, *p* = .35, η_partial_ = .045, nor was there an interaction between hedonic/utilitarian and variety/uniformity, *F*(1, 422) = 1.85, *p* = .17, η_partial_ = .067. In other words, the happiness contribution of varied purchases over uniform purchases was the same for both hedonic and utilitarian spending, refuting Hypothesis 3, although the pattern of the means was consistent with our hypothesis.

However, because we only hypothesized a difference between variety and uniformity for hedonic purchases and did not expect a difference in either direction between variety and uniformity for utilitarian purchases, this interaction represents a relatively imprecise test of our hypothesis of interest. To provide a more specific test of our hypothesis, we examined the difference between varied and uniform spending among only those participants (*n* = 215) who identified hedonic purchases. Participants who identified purchases across 7 hedonic categories reported that their purchases contributed marginally more to their happiness than participants who identified purchases in a single hedonic category, *t*(213) = 1.76, *p* = .079, *d* = 0.24, 95% CI = [-0.028, 0.51]. Although modest, this effect was in the hypothesized direction and would have been significant using a one-tailed test (*p*_one-tailed_ = .040). The results of the real spending experiment therefore partially supported our hypothesis: Participants reported a slightly larger happiness impact from varied hedonic purchases than from uniform hedonic purchases, but this effect was not reliably stronger than the happiness impact of varied utilitarian purchases.

### Hypothetical spending (Study 2 sample)

As illustrated in Fig. 3 (right), a marginal main effect emerged for hedonic spending, such that participants who identified hypothetical hedonic purchases reported that those purchases would bring them greater happiness than participants who identified hypothetical utilitarian purchases, *F*(1, 202) = 2.86, *p* = .092, η_partial_ = .12. The main effect of variety vs. uniformity was nonsignificant, *F*(1, 202) = 0.95, *p* = .33, η_partial_ = .068. There was a marginally significant interaction between hedonic/utilitarian and variety/uniformity, *F*(1, 202) = 3.09, *p* = .080, η_partial_ = .12. Specifically, participants who identified varied hedonic purchases expected marginally greater happiness from those purchases than participants who identified uniform hedonic purchases, *t*(98) = 1.80, *p* = .075, *d* = 0.36, 95% CI [-0.036, 0.75], but no such variety/uniformity difference emerged among participants who identified utilitarian purchases, *t*(104) = -0.60, *p* = .55, *d* = -0.12, 95% CI [-0.50, 0.27].

### Real spending (Study 3 sample)

**Fig. 4:**
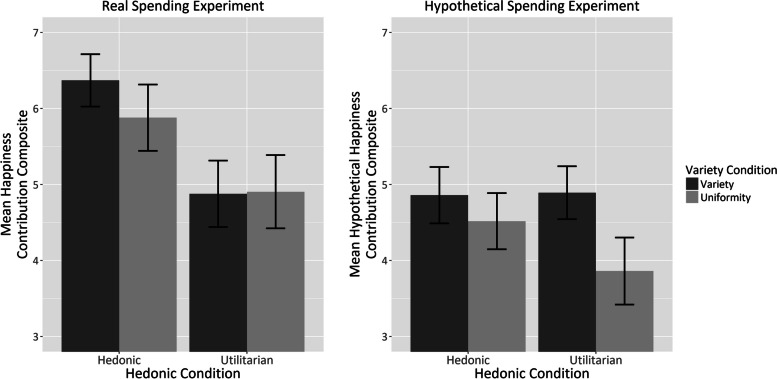
Mean happiness contribution of purchases across hedonic/utilitarian and variety/uniformity conditions in the Study 3 sample experiments. Real experiment: left side, N = 276; hypothetical experiment: right side, N = 444. Error bars are 95% confidence intervals. Happiness contribution scores ranged from 0 to 8

As shown in Fig. 4 (left), a significant main effect of hedonic spending emerged, such that participants who considered hedonic purchases reported more happiness from those purchases than participants who considered utilitarian purchases, *F*(1, 272) = 32.18, *p* < .001, η_partial_ = .33, whereas no main effect emerged for varied vs. uniform purchases, *F*(1, 272) = 1.08, *p* = .30, η_partial_ = .064. Counter to our hypotheses, there was no interaction between hedonic/utilitarian and variety/uniformity, *F*(1, 272) = 1.42, *p* = .23, η_partial_ = .072.

To parse the unique effect of the hedonic variety condition, relative to hedonic uniformity, we examined the difference between varied and uniform spending among only those participants who considered hedonic purchases (*n* = 135). Participants who identified purchases in a variety of hedonic categories reported more happiness from those purchases than participants who identified purchases in just one hedonic category, *t*(133) = 1.75, *p* = .083, *d* = 0.30, 95% CI = [-0.040, 0.64]. Additionally, the meta-analytic (fixed-effects model) *d* of the two samples combined was significant, *d*_meta-analytic_ = 0.27, *t* = 2.45, *p* = .015. Taken together, the results the experiments provide evidence for a small-to-medium effect of varied hedonic spending, relative to uniform hedonic spending, on perceived happiness contributions.

### Hypothetical spending (study 3 sample)

A marginally significant interaction between type of spending and variety emerged, *F*(1, 440) = 3.01, *p* = .079, η_partial_ = .084. As shown in Fig. 4 (right), this interaction was driven by low expected happiness from uniform utilitarian purchases, rather than high expected happiness from varied hedonic purchases. In contrast with the findings from the Study 2 sample, there was no difference between the variety and uniformity conditions among participants who considered hedonic purchases, *t*(235) = 1.29, *p* = .20, *d* = 0.17, although the effect was in the hypothesized direction. Conversely, both planned contrasts were significant using one-tailed tests (contrast 1: *t*[440] = 1.94, *p*_one-tailed_ = .027, *r*_contrast_ = .092; contrast 2: *t*[440] = 1.95, *p*_one-tailed_ = .026, *r*_contrast_ = .092), indicating that the hypothesized patterns of results did significantly fit the data. In sum, participants who considered a variety of hedonic purchases did report relatively high expected happiness from those purchases, but not necessarily to a greater extent than the other types of purchases.

## Discussion

Taken together, our results show that participants who recalled varied hedonic purchases reported greater happiness boosts from those purchases than participants who recalled uniform hedonic purchases. Additionally, hypothetical varied purchases produced greater expected happiness boosts than hypothetical uniform purchases. However, the expected interactions between type of purchase and spending variety were nonsignificant in all four experiments, although the patterns of means (illustrated in Figs. 3 and 4) followed our hypotheses, suggesting that such an interaction may emerge in a more highly-powered experiment. The experimental results thus partially support our prediction that varied hedonic spending imparts a well-being boost, relative to uniform hedonic spending, but this boost cannot be clearly differentiated from the benefits of varied utilitarian spending, relative to uniform utilitarian spending. A critical consideration in interpreting these findings is that the self-report data used in our experiment may not accurately capture the real impact of spending on happiness. Instead, these reports might reflect the participants' preconceived notions or beliefs about spending and happiness, which could be subject to inaccuracies or biases.

## General discussion

We investigated the relationship between spending on hedonic variety and well-being across multiple studies, including both subjective and objective spending data and a pre-registered replication. We examined the question of how hedonic spending variety relates to well-being from two theoretical approaches: the Hedonic Adaption Prevention (HAP) model [12] and broaden-and-build theory [20, 21]. In summary, although we found relatively robust evidence for a cross-sectional correlation between hedonic spending variety and well-being (Hypotheses 1A and 1B), the results examining the causal direction of this relationship were mixed and inconclusive (Hypotheses 2A, 2B, and 3).

### Evidence for a unique association between hedonic spending variety and well-being

Our findings generally supported Hypotheses 1A and 1B, with the cross-sectional results illustrating a correlation between hedonic spending variety and well-being, above and beyond overall financial circumstances and the total amount spent on hedonic goods. According to the HAP model [12], only hedonic spending *variety* should be uniquely related to greater well-being, whereas the overall amount of hedonic spending should have no, or reduced, well-being benefits above and beyond spending variety: Hedonic adaptation minimizes the net happiness boost from spending alone, but variety in that spending can buffer against the detrimental effects of adaptation. However, in Studies 2 and 3, total hedonic spending remained correlated (albeit inconsistently) with well-being even after controlling for spending variety, indicating that total hedonic spending and hedonic variety may have independent associations with well-being. Our finding that overall hedonic spending is a significant predictor of well-being aligns with previous studies in economics examining the link between consumption patterns and well-being. For example, Noll and Weick [44], using data from the German Socio-Economic Panel survey, identified a positive relationship between expenditures on leisure and clothing and higher subjective well-being. Our measure of total hedonic spending includes expenditures in similar categories, which have been historically associated with enhanced well-being. This reinforces the notion that while hedonic variety is important, the sheer volume of hedonic spending also plays a role in influencing well-being.

In Studies 2 and 3, we found that hedonic spending variety was more closely linked to positive affect than to overall life satisfaction. This distinction in impact may stem from the nature of positive affect, which tends to be more responsive to the frequency of positive experiences, as opposed to life satisfaction, a generally less malleable construct [45]. This suggests that positive affect more accurately reflects the advantages of diverse experiences. Furthermore, in line with our hypothesis, hedonic variety did not show a unique relationship with negative affect in either study.

These findings are consistent with previous work on the emotional benefits of forging positive experiences that are frequent rather than intense (e.g., several modest restaurant dinners rather than a single blowout; [46]) and on the rewards of separating rather than combining positive experiences (e.g., rationing out *Game of Thrones* episodes week-by-week rather than binging on several at a time; [47]). Research suggests that the reason dividing spending into smaller chunks increases well-being is because a disproportionate amount of the pleasure of a positive experience is gained from the first portion of the experience. As Pollan [48] put it, “the banquet is in the first bite” (p. 111). Over time, the enjoyment of a pleasurable good or experience declines but can be replenished after a break [6, 42, 49]. Therefore, just as dividing consumption into smaller doses by separating purchases over time can increase the pleasure a person receives from this consumption, so too might spending on varied products and experiences.

Our research adds to the understanding of financial behaviors and well-being by providing evidence from both objective and self-reported data. While previous studies often relied on self-reported purchasing intentions or histories (e.g., [50, 51], we enhanced the reliability of our findings by including actual spending data extracted from transaction records. This approach addresses potential biases in self-reported data and contributes to an emerging field that leverages digitally-recorded behaviors from customer bank accounts to explore psychological phenomena [33, 34, 52–57].

### Evidence for varied hedonic spending causing increased well-being

Our research also set out to test the directionality of the link between hedonic spending variety and well-being. We hypothesized that spending variety might promote greater well-being by mitigating the negative effects of hedonic adaptation. In Studies 2 and 3, we attempted to establish causal relationships through time-lagged assessments. Despite these efforts, the results were not entirely conclusive. In Study 2, our regressed change model controlled for initial levels of well-being and other relevant variables, suggesting that hedonic variety indeed contributes to increased happiness. Conversely, well-being over the follow-up period did not predict concurrent hedonic spending variety above and beyond the amount of variety participants already had. This supports Hypothesis 2A, indicating that spending variety leads to greater well-being, rather than simply being a characteristic of inherently happier individuals (Hypothesis 2B).

The results from Study 2 lend credence to the idea that diversifying experiences can reduce hedonic adaptation, in line with the Hedonic Adaptation Prevention model [14]. However, the absence of a similar lagged effect in Study 3 calls into question the robustness of this finding. While the effect in Study 2 may have been a statistical anomaly, this seems unlikely given the substantial evidence supporting the role of variety in enhancing well-being.

In addition to the time-lagged evidence, in Study 4, we conducted experiments to more directly investigate the causal link between hedonic variety and well-being. Participants who reviewed a range of hedonic purchases perceived these as contributing more to their happiness compared to those who reviewed a single type of purchase. Although these findings aligned with Hypothesis 3, the impact was similar for both hedonic and utilitarian spending, partially challenging our hypothesis. Hence, while varied hedonic purchases were associated with greater recalled happiness, this effect wasn't distinct from the overall impact of hedonic spending.

To further our understanding, future experimental work is necessary to conclusively determine the causal role of spending variety in enhancing well-being. Such research should distinguish spending variety from the total expenditure and examine the immediate impact of varied spending on happiness, beyond just recalled experiences. For instance, manipulating spending behavior to be either varied or uniform and observing its prospective effects on well-being would be invaluable. This approach, combined with our findings, has significant implications for enhancing consumer happiness. For example, large online retailers could encourage hedonic variety via discounts or special offers to incentivize greater variety. Similarly, online recommendation systems based on artificial intelligence (e.g., recommended purchases on Amazon) could potentially be improved by enhancing content variety.

### Evidence for greater well-being causing more varied hedonic spending

Investigating the relationship between well-being and the diversity of hedonic spending, Hypothesis 2B suggested that individuals with higher levels of happiness might engage in more varied spending due to broader interests and perspectives. This hypothesis is in line with the broaden-and-build theory [20, 21], which proposes that positive emotions expand an individual's thought-action repertoire, enhancing their cognitive and behavioral range. The theory posits that positive emotions encourage individuals to engage more with their surroundings and participate in a wider array of activities, potentially leading to more diverse hedonic spending.

The models exploring whether well-being leads to varied hedonic spending, however, offer mixed support for this hypothesis. While the cross-lagged models in Study 3 suggest a potential causal influence of initial well-being on later spending variety, the lack of supportive evidence from Study 2 make it difficult to draw firm conclusions about these causal relationships. Given these mixed findings, further research is needed to clarify when and why hedonic variety might predict concurrent increases in happiness (or vice-versa).

Overall, our results do not robustly support either the HAP model or the broaden-and-build theory, leaving the nature and causal dynamics of the relationship between hedonic spending variety and happiness somewhat unclear. It is possible that both mechanisms are at play, perhaps offsetting some of the expected directional outcomes. Nonetheless, in the context of the replication crisis in psychology [58], we believe it is more valuable to present the full set of our complex and nuanced findings, rather than oversimplifying them. By doing so, we hope to establish a foundation for future research exploring the intricate relationship between varied purchases, or other diverse experiences, and happiness.

### Limitations and future research

Our study focused on the variety of spending across different categories, not within them, which is a significant limitation. This methodology may miss the subtle diversity present within individual categories, as we do not have data on the specific items purchased. Additionally, our approach might not capture the unique joy and satisfaction individuals gain from spending in areas that align more deeply with their self-identity or hobbies [59, 60], such as golf or antique collecting. Such expenditures could offer considerable personal variety and fulfillment, yet may not be adequately represented as varied spending in our analysis. Despite these limitations, the categorization we established in Study 1, and applied in Studies 2 and 3, was designed to highlight meaningful *psychological* differences between spending categories. For example, although the category “Sports other” may contain spending on goods for both rugby and tennis, such purchases are likely not psychologically “varied,” and thus would not bolster well-being even if our theoretical framework is correct. Our results suggest that the inter-category variety provides a credible measure of an individual's overall spending diversity. This finding holds true even though we do not capture the specifics of intra-category spending. In other words, overlooked diversity within categories does not negate our conclusion that broad patterns of varied spending across categories are important for happiness. Nevertheless, future research should consider transaction-level spending data to parse the level (i.e., categories vs. specific purchases) at which varied hedonic spending is most relevant for well-being.

Our analyses implicitly examined spending variety as a stable, trait-like tendency over a given period of time—that is, the models assumed that spending was reliably varied (or consistent) over the full period assessed. However, spending behaviors could be highly varied during a relatively short window of time within the full assessment period but also relatively *stable* outside of that high-variety window. For example, a person’s short-term spending habits may be temporarily more varied than usual when starting a new relationship or moving to a new location. As noted above in the introduction to Hypotheses 2A and 2B, our theoretical model of hedonic adaptation prevention suggests that spending variety offsets adaptation by providing a steady “stream” of novel goods and experiences. However, if all hedonic spending variety occurs in a relatively short period, then adaptation to all hedonic purchases would happen simultaneously, negating any offsetting effects from the variety. Because we assessed spending behaviors retrospectively and cross-sectionally, our operational definition of spending variety cannot differentiate between stable “trait” variety and short-term “state” variety. This conflation of long-term and short-term variety may have resulted in overestimates of *habitual* hedonic spending variety for people who had brief bursts of highly varied purchases. However, if the hedonic benefits of variety do not apply to such individuals (due to more rapid hedonic adaptation), then they also should have had lower well-being than individuals with consistently high hedonic spending variety; as such, any overestimates of habitual variety due to this trait/state conflation should only have diminished the effects of hedonic spending variety on well-being.

Future studies should delve into how temporal patterns in spending affect well-being. Our research primarily focused on the diversity of purchases within a specific timeframe, such as a year, without considering how the timing and sequence of repeated purchases (e.g., A, B, C, A, B, C) might influence hedonic adaptation and overall happiness. This exploration would help provide a deeper understanding of how spending habits develop over time and their subsequent influence on individual happiness and satisfaction. Such research could clarify whether spreading out similar types of purchases over time, thereby creating a pattern of spending, might mitigate hedonic adaptation in a way similar to or even more effectively than a greater variety of purchases. Consider a collector who finds great joy in regularly acquiring items from a single or a few related categories. Similarly, an individual who dedicates a significant portion of their budget to an expensive hobby like golf often reports high satisfaction levels. These examples suggest that consistent spending patterns, even with limited variety, can yield substantial happiness.

Our variety measure may be challenged for its reliance on some potentially unrealistic assumptions. To address this, we tried an alternative method by simply counting the number of categories in each person’s spending. These parallel analyses mostly confirmed our original results. For instance, in the follow-up sample of Study 2, after adjusting for all covariates, we found a significant and positive link between the number of hedonic categories a person spent on and their well-being (β = .13, *p* = .027), but no such link for the number of utilitarian categories (β = -.05, *p* = .448). A similar pattern emerged for positive affect. This finding is consistent with our original results, given the high correlation between our emodiversity-based measure and a simple category count (hedonic, *r* = .80; utilitarian, *r* = .66). However, we preferred the emodiversity-based measure over the simpler count. This choice was based on the emodiversity measure’s demonstrated ability to capture both the variety and balance of spending across different domains, as supported by previous research on emotional variety and well-being [37].

### Generalizability considerations

Our samples drew participants from two “WEIRD” (Western, educated, industrialized, rich, democratic) populations [61]. Consequently, our findings might not be universally applicable, particularly to populations from less wealthy or non-Western cultures. However, the fundamental principles of hedonic adaptation and its mitigation are likely to be consistent across different groups. While cultural variations might influence the specific activities or purchases that bring happiness [62], we expect the process of adapting to these happiness sources and the benefit of variety within them likely remain constant. For instance, the effectiveness of performing varied acts of kindness in enhancing happiness, observed in both American and South Korean contexts [63] illustrates this point.

Additionally, our results suggest that the benefits of hedonic spending variety on well-being are independent of overall wealth and the total amount spent on hedonic goods. This implies that the psychological advantages of diversified spending could be relevant even for those with limited disposable income for hedonic purposes. However, just as cultural differences across nations are likely to influence which goods are considered “hedonic,” so too are socioeconomic differences within a nation, which may also influence what constitutes hedonic spending. A taxi ride, for example, could be a luxury for someone with a lower income but a necessity for someone wealthier. Given that previous research has revealed surprising variability in psychological phenomena between WEIRD and non-WEIRD societies [64], future research should directly test differences between cultural and socioeconomic groups to determine whether the role of hedonic spending variety in well-being—and the general role of variety in preventing hedonic adaptation—varies across groups.

Future research should also explore the motivations behind individuals' preferences for more or less varied spending and whether these preferences correlate with higher well-being. People generally desire variety in their experiences and consumption [65–67], yet there is significant variation in spending diversity, as shown in our treemap plots (Figs 1 and 2). These individual differences in preference for variety could influence the hedonic benefits derived from spending diversity. For example, individuals with a natural inclination for high environmental stimulation, often linked to extraversion [68, 69] might exhibit a stronger relationship between spending variety and well-being. Similarly, those with high levels of openness, a trait associated with a penchant for novelty and varied experiences [70], might naturally choose a wider range of purchases or experiences, enhancing their well-being. Thus, future studies should consider these and other personality traits that might play a role in the complex relationship between spending variety and well-being. Additionally, given that people often fail to predict the affective outcomes of their consumption decisions accurately [71], it would be valuable for future research to investigate people's beliefs about the hedonic impact of spending variety and how these beliefs might affect the observed benefits of varied spending.

### Context and concluding thoughts

In conclusion, our research offers partial support for theories emphasizing the role of variety in the relationship between spending and well-being. Specifically, our findings align with broader models that suggest diversifying experiences can mitigate hedonic adaptation to positive stimuli and indicates that happier individuals may be naturally inclined towards varied experiences. While the effect sizes observed in our studies were modest, even small increases in positive affect can lead to substantial improvements in overall happiness at the population level [72–76]. Our findings suggest that distributing discretionary spending across a wider array of smaller pleasures, rather than concentrating on a few larger ones, could enhance happiness. This spending pattern might also be characteristic of inherently happier individuals. Returning to the story of Mark and Maria, our research suggests that it is, indeed, Maria’s small but varied shopping cart, rather than Mark’s large but unvarying cart, that is linked to the most happiness—but future research is still needed to parse the exact reasons that Maria’s cart is the happier one.

### Supplementary Information

**Supplementary Material 1.** 

## Data Availability

The raw data to recreate the analyses presented in this manuscript are provided on the Open Science Framework (https://osf.io/5vxne/?view_only=3d769a4338a84346b9299d4afd4d88dc). The exception to this is the data used in Study 1, which are owned by a bank in the United Kingdom and the authors do not have permission to share the data publicly. Researchers who meet the criteria for access to confidential data should contact the corresponding author to request access to the Study 1 data.
