# Supplementary material for: Does variety in hedonic spending improve happiness? Testing alternative causal mechanisms between hedonic variety and subjective well-being
Source: BMC Psychol. 2024 Feb 26;12:98. doi: 10.1186/s40359-024-01599-8 (PMC10897990; doi:10.1186/s40359-024-01599-8)
Supplement: Supplementary file 1 — Supplementary Material 1. [file 40359_2024_1599_MOESM1_ESM.docx]

List of spending categories in Study 1. Italicized categories were not evaluated as independent categories, but instead were combined with similar categories.

The Original Classification column reflects the classification derived from external ratings. In some cases, these classifications were amended by the authors (classification marked in italics); the new classifications are noted in the Comments column.

| Spending Category | Original Classification | Comments |
| --- | --- | --- |
| Airports & duty free | Both |  |
| Books | Both |  |
| Catalogue & bargain stores | Both |  |
| Children | Both |  |
| Clothes other | *Both* | RECLASSIFY to Utilitarian to match other clothing categories |
| Computers & technology | Both |  |
| Culture & heritage | Both |  |
| Cycling | Both |  |
| Dairy products | Both |  |
| Department stores | Both |  |
| Direct shopping other | Both |  |
| foreign air travel | Both |  |
| Gardening | Both |  |
| General retail | Both |  |
| Health & fitness | Both |  |
| *Home furnishing* | Both | COMBINE to “Furniture [all]” |
| Home improvement | Both |  |
| Hotels | Both |  |
| Ladies’ clothes | *Both* | RECLASSIFY to Utilitarian to match other clothing categories |
| Mobile telephone | Both |  |
| *Newspapers & magazines* | *Both* | RECLASSIFY to Utilitarian and COMBINE to “News [all]” |
| Radio & electrical | Both |  |
| Shoe shops | Both |  |
| Swimming | Both |  |
| Wildlife | Both |  |
| Antiques | Hedonic |  |
| *Art* | Hedonic | COMBINE to “Art institutions” |
| *Arts* | Hedonic | COMBINE to “Art institutions” |
| Arts & crafts | Hedonic |  |
| Athletics | Hedonic |  |
| Bakers & confectioners | Hedonic |  |
| Billiards & pool | Hedonic |  |
| Bowling | Hedonic |  |
| *Cable & satellite TV* | Hedonic | COMBINE to “Cable & satellite TV [all]” |
| *Cable TV* | Hedonic | COMBINE to “Cable & satellite TV [all]” |
| Caravans & camping | Hedonic |  |
| Cinema | Hedonic |  |
| Clubs & societies | Hedonic |  |
| Coffee Shops | Hedonic |  |
| Confectioners & tobacconists | Hedonic |  |
| Cricket | Hedonic |  |
| Dancing | Hedonic |  |
| Days out & UK tourism | Hedonic |  |
| Eating out - pubs | Hedonic |  |
| Eating out - restaurants | Hedonic |  |
| Entertainment | Hedonic |  |
| Escort & introduction agencies | Hedonic |  |
| Florists | Hedonic |  |
| *Football* | Hedonic | COMBINE to “Professional Football” |
| foreign travel other | Hedonic |  |
| Gambling | Hedonic |  |
| Gift shops | Hedonic |  |
| Golf | Hedonic |  |
| Gymnastics | Hedonic |  |
| Hair & beauty | Hedonic |  |
| Jewellery | Hedonic |  |
| Massage parlours | Hedonic |  |
| Music | Hedonic |  |
| Off licences | Hedonic |  |
| Pets | Hedonic |  |
| Photography | Hedonic |  |
| *Premiership Football* | Hedonic | COMBINE to “Professional Football” |
| Rambling & climbing | Hedonic |  |
| Sailing | Hedonic |  |
| Sport other | Hedonic |  |
| Sports clubs | Hedonic |  |
| Takeaways | Hedonic |  |
| Television other | Hedonic |  |
| Tennis & squash | Hedonic |  |
| Theatrical clothing hire | Hedonic |  |
| Toys & hobbies | Hedonic |  |
| Travel agents | Hedonic |  |
| *Travel other* | *Hedonic* | RECLASSIFY to Utilitarian (clearly misinterpreted by raters; refers to bus tolls, taxi fees, etc.) and COMBINE to “Transport [all]” |
| Videos | Hedonic |  |
| Wine | Hedonic |  |
| Boys & girls camps | Neither |  |
| Buying & selling clubs | Neither |  |
| Catalogue shopping | Neither |  |
| China & glass | Neither |  |
| Credit cards | Neither |  |
| Extended Warranty | Neither |  |
| *Friendly society other* | *Neither* | RECLASSIFY to Utilitarian and COMBINE to “Insurance other [all]” (refers to a type of insurance org; suspect that raters just didn’t know what this is) |
| Furriers | Neither |  |
| Leather goods & luggage | Neither |  |
| Motorcycling | Neither |  |
| Other services | Neither |  |
| Religious other | Neither |  |
| Retail other | Neither |  |
| Stamps & coins | Neither |  |
| Storecards | Neither |  |
| Subscriptions | Neither |  |
| TV rental | Neither |  |
| Academic | *Utilitarian* | RECLASSIFY to Neither (just one person’s MENSA dues) |
| Accountants’ fees | Utilitarian |  |
| Advertising services | Utilitarian |  |
| Agricultural | Utilitarian |  |
| *Animal charities* | Utilitarian | COMBINE to “Charity [all]” |
| *ATM cash* | Utilitarian | COMBINE to “Cash [all]” |
| Automative equipment | Utilitarian |  |
| Bank other | Utilitarian |  |
| Bike Insurance | Utilitarian |  |
| *Branch cash* | Utilitarian | COMBINE to “Cash [all]” |
| *BT* | Utilitarian | COMBINE to “Telecom [all]” |
| *Building professionals* | Utilitarian | COMBINE to “Unique professional organizations” |
| Building society other | Utilitarian |  |
| Business & finance | Utilitarian |  |
| Business & secretarial schools | Utilitarian |  |
| Car dealers | Utilitarian |  |
| Car hire | Utilitarian |  |
| Car other | Utilitarian |  |
| Car parks | Utilitarian |  |
| Cash & carry | Utilitarian |  |
| *Charities other* | Utilitarian | COMBINE to “Charity [all]” |
| *Charity appeals* | Utilitarian | COMBINE to “Charity [all]” |
| Chemists | Utilitarian |  |
| Child day care services | Utilitarian |  |
| Child Trust Fund | Utilitarian |  |
| *Children’s charities* | Utilitarian | COMBINE to “Charity [all]” |
| Children’s clothes | Utilitarian |  |
| Civil service | Utilitarian |  |
| Cleaning | Utilitarian |  |
| *Colleges* | Utilitarian | COMBINE to “Colleges & universities” |
| Commercial supplies | Utilitarian |  |
| Conference organisers | Utilitarian |  |
| Consultants | Utilitarian |  |
| Counselling services | Utilitarian |  |
| Credit card related | Utilitarian |  |
| Credit Reporting Agencies | Utilitarian |  |
| Dental care | Utilitarian |  |
| Digital | Utilitarian |  |
| Discount stores | Utilitarian |  |
| DIY | Utilitarian |  |
| *Doctors* | Utilitarian | COMBINE to “General health” |
| Driving schools & education | Utilitarian |  |
| DSS | Utilitarian |  |
| Education other | Utilitarian |  |
| Education profession | Utilitarian |  |
| Electricians | Utilitarian |  |
| Electricity | Utilitarian |  |
| Electronic commerce & IT | Utilitarian |  |
| Elementary & secondary schools | Utilitarian |  |
| Employment agencies | Utilitarian |  |
| Energy | Utilitarian |  |
| Engineering companies & surveyors | Utilitarian |  |
| *Engineers* | Utilitarian | COMBINE to “Unique professional organizations” |
| *Environmental charities* | Utilitarian | COMBINE to “Charity [all]” |
| Fabrics, wool & sewing | Utilitarian |  |
| Family clothes | Utilitarian |  |
| Farming & forestry | Utilitarian |  |
| *Financial services other* | Utilitarian | COMBINE to “Financial services other [all]” |
| Food retailers | Utilitarian |  |
| foreign currency & VAT refunds | Utilitarian |  |
| Frozen foods | Utilitarian |  |
| Fund Managers | Utilitarian |  |
| Funeral parlours | Utilitarian |  |
| *Furniture* | *Utilitarian* | RECLASSIFY to Both and COMBINE to “Furniture [all]” (functionally equivalent to “Home furnishing,” which is unambiguously rated as both) |
| Gas | Utilitarian |  |
| Government agencies | Utilitarian |  |
| Government postal services | Utilitarian |  |
| Graduates | Utilitarian |  |
| Hardware | Utilitarian |  |
| Health insurance | Utilitarian |  |
| *Health other* | Utilitarian | COMBINE to “General health” |
| Hearing aids | Utilitarian |  |
| Heating & plumbing | Utilitarian |  |
| Home Breakdown Cover | Utilitarian |  |
| *Home insurance* | Utilitarian |  |
| Hospitals | Utilitarian | COMBINE to “General health” |
| HR professionals | Utilitarian |  |
| *Human rights charities* | Utilitarian | COMBINE to “Charity [all]” |
| Information retrieval | Utilitarian |  |
| *Insurance other* | Utilitarian | COMBINE to “Insurance other [all]” |
| Intra-government purchases | Utilitarian |  |
| Investment | Utilitarian |  |
| Janitorial services | Utilitarian |  |
| Laundry | Utilitarian |  |
| Life assurance | Utilitarian |  |
| Loans | Utilitarian |  |
| Local council | Utilitarian |  |
| *Local transport* | Utilitarian | COMBINE to “Transport [all]” |
| Manufacturing | Utilitarian |  |
| *Medical charities* | Utilitarian | COMBINE to “Charity [all]” |
| *Medical professionals* | Utilitarian | COMBINE to “General health” |
| Menswear | Utilitarian |  |
| Miscellaneous services | Utilitarian |  |
| Money orders & wire transfers | Utilitarian |  |
| Mortgage protection | Utilitarian |  |
| Motor insurance | Utilitarian |  |
| Motor rescue | Utilitarian |  |
| Motoring other | Utilitarian |  |
| New finance other | Utilitarian |  |
| *News media* | Utilitarian | COMBINE to “News [all]” |
| *Newsagents* | Utilitarian | COMBINE to “News [all]” |
| Online Platform | Utilitarian |  |
| Opticians | Utilitarian |  |
| Orthopaedic goods | Utilitarian |  |
| *Other Finance* | Utilitarian | COMBINE to “Financial services other [all]” |
| Pawnbrokers | Utilitarian |  |
| Pensioners | Utilitarian |  |
| Pensions | Utilitarian |  |
| Pet Insurance | Utilitarian |  |
| Petrol | Utilitarian |  |
| Politics & current affairs | Utilitarian |  |
| Prison service | Utilitarian |  |
| Private Medical Cover | Utilitarian |  |
| Professional services other | Utilitarian |  |
| Publishing & printing | Utilitarian |  |
| Rent | Utilitarian |  |
| Repair shops | Utilitarian |  |
| Residential Mortgages | Utilitarian |  |
| Sales & marketing | Utilitarian |  |
| Salvage yards | Utilitarian |  |
| Savings | Utilitarian |  |
| Scientists | Utilitarian |  |
| Secretarial & support services | Utilitarian |  |
| Security | Utilitarian |  |
| Sharesave | Utilitarian |  |
| Short Term Borrowing | Utilitarian |  |
| Sign makers & writers | Utilitarian |  |
| Solicitors | Utilitarian |  |
| Stationery | Utilitarian |  |
| Stockbrokers | Utilitarian |  |
| Supermarkets | Utilitarian |  |
| Surplus stores | Utilitarian |  |
| Surveyors | Utilitarian |  |
| Taxation | Utilitarian |  |
| *Telephone other* | Utilitarian | COMBINE to “Telecom [all]” |
| *Third world charities* | Utilitarian | COMBINE to “Charity [all]” |
| Tool hire | Utilitarian |  |
| Traffic fines | Utilitarian |  |
| *Transport* | Utilitarian | COMBINE to “Transport [all]” |
| Travel insurance | Utilitarian |  |
| TV licence | Utilitarian |  |
| *UK rail transport* | Utilitarian | COMBINE to “Transport [all]” |
| Unions & other subscriptions | Utilitarian |  |
| *Universities* | Utilitarian | COMBINE to “Colleges & universities” |
| University Application | Utilitarian |  |
| Vocational schools | Utilitarian |  |
| Water | Utilitarian |  |

List of spending categories in Sample 2

| Spending category | Classification |
| --- | --- |
| Airports & duty free | Both |
| Books | Both |
| Catalogue & bargain stores | Both |
| Children | Both |
| Computers & technology | Both |
| Culture & heritage | Both |
| Cycling | Both |
| Dairy products | Both |
| Department stores | Both |
| foreign air travel | Both |
| foreign travel other | Both |
| Furniture_all | Both |
| Gardening | Both |
| Health & fitness | Both |
| Home improvement | Both |
| Hotels | Both |
| Mobile telephone | Both |
| Radio & electrical | Both |
| Shoe shops | Both |
| Swimming | Both |
| Wildlife | Both |
| Antiques | Hedonic |
| Art institutions | Hedonic |
| Arts & crafts | Hedonic |
| Athletics | Hedonic |
| Bakers & confectioners | Hedonic |
| Billiards & pool | Hedonic |
| Bowling | Hedonic |
| Cable & satellite TV_all | Hedonic |
| Caravans & camping | Hedonic |
| Cinema | Hedonic |
| Clubs & societies | Hedonic |
| Coffee Shops | Hedonic |
| Confectioners & tobacconists | Hedonic |
| Cricket | Hedonic |
| Dancing | Hedonic |
| Days out & UK tourism | Hedonic |
| Eating out - pubs | Hedonic |
| Eating out - restaurants | Hedonic |
| Entertainment | Hedonic |
| Escort & introduction agencies | Hedonic |
| Florists | Hedonic |
| Gambling | Hedonic |
| Gift shops | Hedonic |
| Golf | Hedonic |
| Gymnastics | Hedonic |
| Hair & beauty | Hedonic |
| Jewellery | Hedonic |
| Massage parlours | Hedonic |
| Music | Hedonic |
| Off licences | Hedonic |
| Pets | Hedonic |
| Photography | Hedonic |
| Professional Football | Hedonic |
| Rambling & climbing | Hedonic |
| Sailing | Hedonic |
| Sport other | Hedonic |
| Sports clubs | Hedonic |
| Takeaways | Hedonic |
| Television other | Hedonic |
| Tennis & squash | Hedonic |
| Theatrical clothing hire | Hedonic |
| Toys & hobbies | Hedonic |
| Travel agents | Hedonic |
| Videos | Hedonic |
| Wine | Hedonic |
| Accountants’ fees | Utilitarian |
| Advertising services | Utilitarian |
| Agricultural | Utilitarian |
| Automative equipment | Utilitarian |
| Bike Insurance | Utilitarian |
| Building society other | Utilitarian |
| Business & finance | Utilitarian |
| Business & secretarial schools | Utilitarian |
| Car dealers | Utilitarian |
| Car hire | Utilitarian |
| Car other | Utilitarian |
| Car parks | Utilitarian |
| Cash | Utilitarian |
| Cash & carry | Utilitarian |
| Charity | Utilitarian |
| Chemists | Utilitarian |
| Child Trust Fund | Utilitarian |
| Children’s clothes | Utilitarian |
| Civil service | Utilitarian |
| Cleaning | Utilitarian |
| Clothes other | Utilitarian |
| Colleges & universities | Utilitarian |
| Commercial supplies | Utilitarian |
| Conference organisers | Utilitarian |
| Consultants | Utilitarian |
| Counselling services | Utilitarian |
| Credit card related | Utilitarian |
| Credit Reporting Agencies | Utilitarian |
| Dental care | Utilitarian |
| Discount stores | Utilitarian |
| DIY | Utilitarian |
| Driving schools & education | Utilitarian |
| DSS | Utilitarian |
| Education other | Utilitarian |
| Education profession | Utilitarian |
| Electricians | Utilitarian |
| Electricity | Utilitarian |
| Electronic commerce & IT | Utilitarian |
| Elementary & secondary schools | Utilitarian |
| Employment agencies | Utilitarian |
| Engineering companies & surveyors | Utilitarian |
| Fabrics, wool & sewing | Utilitarian |
| Family clothes | Utilitarian |
| Farming & forestry | Utilitarian |
| Food retailers | Utilitarian |
| Frozen foods | Utilitarian |
| Fund Managers | Utilitarian |
| Funeral parlours | Utilitarian |
| Gas | Utilitarian |
| General health | Utilitarian |
| Government agencies | Utilitarian |
| Government postal services | Utilitarian |
| Graduates | Utilitarian |
| Hardware | Utilitarian |
| Health insurance | Utilitarian |
| Hearing aids | Utilitarian |
| Heating & plumbing | Utilitarian |
| Home Breakdown Cover | Utilitarian |
| Home insurance | Utilitarian |
| HR professionals | Utilitarian |
| Information retrieval | Utilitarian |
| Insurance other_all | Utilitarian |
| Intra-government purchases | Utilitarian |
| Investment | Utilitarian |
| Janitorial services | Utilitarian |
| Ladies’ clothes | Utilitarian |
| Laundry | Utilitarian |
| Life assurance | Utilitarian |
| Loans | Utilitarian |
| Local council | Utilitarian |
| Manufacturing | Utilitarian |
| Menswear | Utilitarian |
| Misc financial services | Utilitarian |
| Money orders & wire transfers | Utilitarian |
| Mortgage protection | Utilitarian |
| Motor insurance | Utilitarian |
| Motor rescue | Utilitarian |
| Motoring other | Utilitarian |
| News | Utilitarian |
| Online Platform | Utilitarian |
| Opticians | Utilitarian |
| Orthopaedic goods | Utilitarian |
| Pawnbrokers | Utilitarian |
| Pensions | Utilitarian |
| Pet Insurance | Utilitarian |
| Petrol | Utilitarian |
| Politics & current affairs | Utilitarian |
| Prison service | Utilitarian |
| Private Medical Cover | Utilitarian |
| Publishing & printing | Utilitarian |
| Rent | Utilitarian |
| Repair shops | Utilitarian |
| Residential Mortgages | Utilitarian |
| Sales & marketing | Utilitarian |
| Salvage yards | Utilitarian |
| Savings | Utilitarian |
| Scientists | Utilitarian |
| Secretarial & support services | Utilitarian |
| Security | Utilitarian |
| Sharesave | Utilitarian |
| Short Term Borrowing | Utilitarian |
| Sign makers & writers | Utilitarian |
| Solicitors | Utilitarian |
| Stationery | Utilitarian |
| Stockbrokers | Utilitarian |
| Supermarkets | Utilitarian |
| Surplus stores | Utilitarian |
| Surveyors | Utilitarian |
| Taxation | Utilitarian |
| Telecom | Utilitarian |
| Tool hire | Utilitarian |
| Traffic fines | Utilitarian |
| Transport_all | Utilitarian |
| Travel insurance | Utilitarian |
| TV licence | Utilitarian |
| Unions & other subscriptions | Utilitarian |
| Unique professional organizations | Utilitarian |
| University Application | Utilitarian |
| Vocational schools | Utilitarian |
| Water | Utilitarian |

List of spending categories in Sample 3

| Spending category | Classification |
| --- | --- |
| Airports & duty free | Both |
| Books | Both |
| Catalog & bargain stores | Both |
| Children | Both |
| Computers & technology | Both |
| Culture & heritage | Both |
| Cycling | Both |
| Department stores | Both |
| foreign air travel | Both |
| foreign travel other | Both |
| Gardening | Both |
| Health & fitness | Both |
| Home improvement | Both |
| Hotels | Both |
| Mobile telephone | Both |
| Radio & electrical | Both |
| Shoe shops | Both |
| Swimming | Both |
| Wildlife | Both |
| Antiques | Hedonic |
| Art institutions | Hedonic |
| Arts & crafts | Hedonic |
| Bakers & confectioners | Hedonic |
| Billiards & pool | Hedonic |
| Bowling | Hedonic |
| Cable & satellite TV | Hedonic |
| Caravans & camping | Hedonic |
| Carry-out | Hedonic |
| Cinema | Hedonic |
| Clubs & societies | Hedonic |
| Coffee shops | Hedonic |
| Convenience store | Hedonic |
| Costume store | Hedonic |
| Dancing | Hedonic |
| Dating services & escorts | Hedonic |
| Days out & US tourism | Hedonic |
| Eating out - bars | Hedonic |
| Eating out - restaurants | Hedonic |
| Entertainment | Hedonic |
| Florists | Hedonic |
| Gambling | Hedonic |
| Gift shops | Hedonic |
| Golf | Hedonic |
| Gymnastics | Hedonic |
| Hair & beauty | Hedonic |
| Hiking & climbing | Hedonic |
| Jewelry | Hedonic |
| Liquor store | Hedonic |
| Live sports events | Hedonic |
| Massage parlors | Hedonic |
| Music | Hedonic |
| Pets | Hedonic |
| Photography | Hedonic |
| Sailing | Hedonic |
| Sports clubs | Hedonic |
| Sports other | Hedonic |
| Tennis & squash | Hedonic |
| Toys & hobbies | Hedonic |
| Track and field | Hedonic |
| Travel agents | Hedonic |
| Videos | Hedonic |
| Wine | Hedonic |
| Internet streaming services | Undetermined |
| Accountants’ fees | Utilitarian |
| Advertising services | Utilitarian |
| Agricultural | Utilitarian |
| Attorney fees | Utilitarian |
| Auto insurance | Utilitarian |
| Automotive equipment | Utilitarian |
| Bike insurance | Utilitarian |
| Business & finance | Utilitarian |
| Business & secretarial schools | Utilitarian |
| Car dealers | Utilitarian |
| Car other | Utilitarian |
| Car rental | Utilitarian |
| Cash | Utilitarian |
| Charity | Utilitarian |
| Children’s clothes | Utilitarian |
| Cleaning | Utilitarian |
| Clothes for family members | Utilitarian |
| Clothes other | Utilitarian |
| Colleges & universities | Utilitarian |
| Commercial supplies | Utilitarian |
| Conferences | Utilitarian |
| Consultants | Utilitarian |
| Counseling services | Utilitarian |
| Credit reporting agencies | Utilitarian |
| Dental care | Utilitarian |
| Discount stores | Utilitarian |
| DIY | Utilitarian |
| Driving schools & education | Utilitarian |
| Education other | Utilitarian |
| Education profession | Utilitarian |
| Electricians | Utilitarian |
| Electricity | Utilitarian |
| Electronic commerce & IT | Utilitarian |
| Elementary & secondary schools | Utilitarian |
| Employment agencies | Utilitarian |
| Engineering companies | Utilitarian |
| Fabrics, wool & sewing | Utilitarian |
| Farming & forestry | Utilitarian |
| Food retailers | Utilitarian |
| Frozen foods | Utilitarian |
| Fund managers | Utilitarian |
| Funerals | Utilitarian |
| Gas | Utilitarian |
| Gasoline | Utilitarian |
| Government agencies | Utilitarian |
| Government postal services | Utilitarian |
| Government services | Utilitarian |
| Hardware | Utilitarian |
| Health insurance | Utilitarian |
| Health insurance premiums | Utilitarian |
| Hearing aids | Utilitarian |
| Heating & plumbing | Utilitarian |
| Home breakdown insurance | Utilitarian |
| Home insurance | Utilitarian |
| HR professionals | Utilitarian |
| Information retrieval | Utilitarian |
| Intra-government purchases | Utilitarian |
| Investment | Utilitarian |
| Janitorial services | Utilitarian |
| Ladies’ clothes | Utilitarian |
| Laundry | Utilitarian |
| Life insurance | Utilitarian |
| Loans | Utilitarian |
| Local government | Utilitarian |
| Manufacturing | Utilitarian |
| Menswear | Utilitarian |
| Money orders & wire transfers | Utilitarian |
| Mortgage protection | Utilitarian |
| News | Utilitarian |
| Online platform | Utilitarian |
| Opticians | Utilitarian |
| Orthopedic goods | Utilitarian |
| Other automotive | Utilitarian |
| Other transport | Utilitarian |
| Parking | Utilitarian |
| Pawn shop | Utilitarian |
| Pensions | Utilitarian |
| Pet insurance | Utilitarian |
| Pharmacists | Utilitarian |
| Phone company | Utilitarian |
| Politics & current affairs | Utilitarian |
| Prison service | Utilitarian |
| Publishing & printing | Utilitarian |
| Rent | Utilitarian |
| Repair shops | Utilitarian |
| Residential mortgages | Utilitarian |
| Roadside assistance | Utilitarian |
| Sales & marketing | Utilitarian |
| Salvage yards | Utilitarian |
| Savings | Utilitarian |
| Secretarial & support services | Utilitarian |
| Security | Utilitarian |
| Short term borrowing | Utilitarian |
| Sign makers & writers | Utilitarian |
| Stationery | Utilitarian |
| Stockbrokers | Utilitarian |
| Supermarkets | Utilitarian |
| Surplus stores | Utilitarian |
| Surveyors | Utilitarian |
| Taxation | Utilitarian |
| Tool rental | Utilitarian |
| Traffic fines | Utilitarian |
| Travel agents | Utilitarian |
| Travel insurance | Utilitarian |
| Unions & other subscriptions | Utilitarian |
| University application | Utilitarian |
| Vocational schools | Utilitarian |
| Water | Utilitarian |
| Wholesalers | Utilitarian |

| Table S1  *Descriptive Statistics from Sample 1* | | | | | |
| --- | --- | --- | --- | --- | --- |
|  | Mean | Standard deviation | Minimum | Median | Maximum |
| Age in years | 37.21 | 14.4 | 18 | 33 | 75 |
| Life satisfaction | 3.15 | 0.89 | 1.00 | 3.20 | 5.00 |
| Hedonic variety | 1.56 | 0.53 | 0.00 | 1.66 | 2.63 |
| Utilitarian variety | 2.05 | 0.38 | 0.27 | 2.08 | 2.86 |
| Hedonic spending (prior 12 months, £) | 1635.30 | 1583.37 | 0.00 | 1230.93 | 15260.12 |
| Utilitarian spending (prior 12 months, £) | 8729.86 | 6659.86 | 652.67 | 6837.83 | 45322.80 |
| Income (annual, £) | 24899.53 | 19875.48 | 767.04 | 19907.04 | 222472.92 |
| Liquid wealth (monthly, £) | 4115.35 | 8980.54 | -1653.83 | 914.25 | 73768.50 |
| Investments (time-of-study, £) | 4209.65 | 27769.61 | 0.00 | 0.00 | 400000.00 |
| *Categorical Variables* | Category | *n* |  |  |  |
| Gender (1 missing) | Female | 287 |  |  |  |
|  | Male | 239 |  |  |  |
| Relationship status (13 missing) | Married or cohabitating | 292 |  |  |  |
|  | Not married or cohab. | 235 |  |  |  |
| Employment status (0 missing) | Employed | 401 |  |  |  |
|  | Student | 30 |  |  |  |
|  | Retired | 42 |  |  |  |
|  | None | 54 |  |  |  |
| Debt status (0 missing) | Has debt | 212 |  |  |  |
|  | No debt | 315 |  |  |  |
| *Note*. Spending, income, and liquid wealth were bank-reported; investments were self-reported. Life satisfaction was measured using the Satisfaction With Life Scale (Diener et al., 1985) on a 5-point Likert scale. Total *N* = 527. | | | | | |

| Table S2  *Correlation Matrix of Sample 1 (Bank-Reported Data) Variables* | | | | | | | | | | | |
| --- | --- | --- | --- | --- | --- | --- | --- | --- | --- | --- | --- |
|  |  | A | B | C | D | E | F | G | H | I | J |
| A | Life satisfaction |  |  |  |  |  |  |  |  |  |  |
| B | Hedonic variety | .12** |  |  |  |  |  |  |  |  |  |
| C | Utilitarian variety | .07 | .25*** |  |  |  |  |  |  |  |  |
| D | log Total hedonic spending | .17*** | .41*** | .29*** |  |  |  |  |  |  |  |
| E | log Total utilitarian spending | .04 | .08 | .28*** | .28*** |  |  |  |  |  |  |
| F | Age | -.06 | -.42*** | .02 | -.18*** | .37*** |  |  |  |  |  |
| G | Married/cohabitating | .12** | .00 | .11* | .09* | .27*** | .13** |  |  |  |  |
| H | log Income | .11* | -.02 | .27*** | .33*** | .66*** | .28*** | .25*** |  |  |  |
| I | log Liquid wealth | .18*** | -.15*** | .05 | .09* | .23*** | .24*** | -.03 | .29*** |  |  |
| J | log Investments | .12** | -.06 | .10* | .09* | .21*** | .15*** | .02 | .24*** | .19*** |  |
| K | Debt status (1 = has debt) | -.13** | .02 | .15*** | -.02 | .18*** | .07 | .16*** | .13** | -.25*** | .01 |
| *Note*. **p* < .05. ***p* < .01. ****p* < .001. *N* = 527.  Spending, income, and liquid wealth were bank-reported; demographics, investments, and debt were self-reported. | | | | | | | | | | | |

| Table S3  *Preliminary Regression Models Predicting Life Satisfaction (Sample 1)* | | | | | | | | | |
| --- | --- | --- | --- | --- | --- | --- | --- | --- | --- |
|  |  | Demographics only | | |  |  | Financial variables | | |
| Predictor |  | *B* (*SE*) |  | β |  |  | *B* (*SE*) |  | β |
| Intercept |  | 2.76 (0.19) |  | *** |  |  | 1.54 (0.48) |  | ** |
| Age | - | 0.01 (0.00) | - | .13* |  | - | 0.01 (0.00) | - | .16** |
| Married/cohabitating |  | 0.23 (0.08) |  | .13** |  |  | 0.25 (0.08) |  | .14** |
| Employed |  | 0.53 (0.13) |  | .26*** |  |  | 0.45 (0.13) |  | .22*** |
| Student |  | 0.69 (0.21) |  | .18*** |  |  | 0.61 (0.20) |  | .16** |
| Retired |  | 0.98 (0.20) |  | .30*** |  |  | 0.77 (0.20) |  | .24*** |
| Female |  | 0.08 (0.08) |  | .04 |  |  | 0.09 (0.08) |  | .05 |
| log Income |  |  |  |  |  |  | 0.14 (0.14) |  | .05 |
| log Liquid wealth |  |  |  |  |  |  | 0.26 (0.11) |  | .12* |
| log Investments |  |  |  |  |  |  | 0.07 (0.03) |  | .11* |
| Debt status (1 = has debt) |  |  |  |  |  | - | 0.16 (0.08) | - | .09* |
| *R*^2^ (adjusted *R*^2^) |  | .081 (.067) | | |  |  | .13 (.11) | | |
| Δ*R*^2^ |  |  |  |  |  |  | .047 |  |  |
| Model *F* (*df*_numerator_, *df*_denominator_) |  | 5.74 (8, 518) | | |  |  | 6.29 (12, 514) | | |
| *F*-change (*df*_numerator_, *df*_denominator_) |  |  | | |  |  | 6.87 (4, 514) | | |
| *Note*. **p* < .05. ***p* < .01. ****p* < .001.  Nominal demographic variables (e.g., employment) are dummy coded. Missingness dummy variables are not reported. Spending, income, and liquid wealth were bank-reported; demographics, investments, and debt were self-reported. | | | | | | | | | |

| Table S4  *Regression Models for Spending and Spending Variety Predicting Life Satisfaction (Sample 1, Hypothesis 1B)* | | | | | | | | | | | | | | | |
| --- | --- | --- | --- | --- | --- | --- | --- | --- | --- | --- | --- | --- | --- | --- | --- |
|  |  | Model 1: Utilitarian spending and variety | | |  |  | Model 2: Hedonic spending | | |  |  | Model 3: Hedonic variety | | | |
| Predictor |  | *B* (*SE*) |  | β |  |  | *B* (*SE*) |  | β |  |  | *B* (*SE*) |  | β | *r*_semipartial_ |
| Intercept |  | 1.68 (0.54) |  | ** |  |  | 1.58 (0.54) |  | ** |  |  | 1.47 (0.54) |  | ** |  |
| Age | - | 0.01 (0.00) | - | .15** |  | - | 0.01 (0.00) | - | .11† |  | - | 0.00 (0.00) | - | .07 | .04 |
| Married/cohabitating |  | 0.26 (0.08) |  | .14** |  |  | 0.26 (0.08) |  | .14** |  |  | 0.26 (0.08) |  | .15** | .13 |
| Employed |  | 0.44 (0.13) |  | .21*** |  |  | 0.43 (0.13) |  | .21*** |  |  | 0.44 (0.13) |  | .21*** | .14 |
| Student |  | 0.61 (0.20) |  | .16** |  |  | 0.61 (0.20) |  | .16** |  |  | 0.63 (0.20) |  | .16** | .13 |
| Retired |  | 0.75 (0.20) |  | .23*** |  |  | 0.71 (0.20) |  | .22*** |  |  | 0.72 (0.20) |  | .22*** | .14 |
| Female |  | 0.09 (0.08) |  | .05 |  |  | 0.11 (0.08) |  | .06 |  |  | 0.08 (0.08) |  | .05 | .05 |
| log Income |  | 0.20 (0.17) |  | .07 |  |  | 0.13 (0.17) |  | .04 |  |  | 0.18 (0.17) |  | .06 | .04 |
| log Liquid wealth |  | 0.27 (0.11) |  | .12* |  |  | 0.27 (0.11) |  | .12* |  |  | 0.29 (0.11) |  | .13** | .11 |
| log Investments |  | 0.07 (0.03) |  | .11* |  |  | 0.07 (0.03) |  | .11* |  |  | 0.07 (0.03) |  | .11** | .11 |
| Debt status (1 = has debt) | - | 0.16 (0.08) | - | .09* |  | - | 0.14 (0.08) | - | .08† |  | - | 0.14 (0.08) | - | .08† | .07 |
| log Utilitarian spending | - | 0.13 (0.16) | - | .05 |  | - | 0.19 (0.16) | - | .07 |  | - | 0.26 (0.16) | - | .10 | .07 |
| Utilitarian variety |  | 0.06 (0.10) |  | .03 |  |  | 0.01 (0.10) |  | .01 |  | - | 0.02 (0.11) | - | .01 | .01 |
| log Hedonic spending |  |  |  |  |  |  | 0.18 (0.08) |  | .11* |  |  | 0.13 (0.09) |  | .07 | .06 |
| Hedonic variety |  |  |  |  |  |  |  |  |  |  |  | 0.18 (0.09) |  | .11* | .09 |
| *R*^2^ (adjusted *R*^2^) |  | .13 (.11) | | |  |  | .14 (.11) | | |  |  | .15 (.12) | | |  |
| Δ*R*^2^ |  | .002 |  |  |  |  | .008 |  |  |  |  | .007 |  |  |  |
| Model *F* (*df*_numerator_, *df*_denominator_) |  | 5.45 (14, 512) | | |  |  | 5.46 (15, 511) | | |  |  | 5.43 (16, 510) | | |  |
| *F*-change (*df*_numerator_, *df*_denominator_) |  | 0.46 (2, 512) | | |  |  | 5.00 (1, 511) | | |  |  | 4.46 (1, 510) | | |  |
| *Note*. †*p* < .10. **p* < .05. ***p* < .01. ****p* < .001.  Nominal demographic variables (e.g., employment) are dummy coded. Missingness dummy variables are not reported. Spending, income, and liquid wealth were bank-reported; demographics, investments, and debt were self-reported. | | | | | | | | | | | | | | | |

| Table S5  *Descriptive Statistics from Sample 2, Time 1* | | | | | | |
| --- | --- | --- | --- | --- | --- | --- |
|  | Mean | Standard deviation | Minimum | Median | Maximum | |
| Age in years | 35.22 | 12.11 | 18 | 33 | 73 | |
| Life satisfaction | 4.17 | 1.29 | 1.00 | 4.40 | 7.00 | |
| Positive affect | 4.32 | 1.29 | 1.00 | 4.50 | 7.00 | |
| Hedonic variety | 1.72 | 0.62 | 0.00 | 1.85 | 3.15 | |
| Utilitarian variety | 1.84 | 0.62 | 0.00 | 1.93 | 3.49 | |
| Hedonic spending (prior 12 months, £) | 2401.43 | 4331.60 | 0.00 | 1490.00 | 100813.00 | |
| Utilitarian spending (prior 12 months, £) | 73650.52 | 1746712.97 | 0.00 | 11696.50 | 55025554.00 | |
| Income (annual, £) | 20157.96 | 29969.48 | 0.00 | 16000.00 | 697000.00 | |
| Liquid wealth (monthly, £) | 8161.10 | 33716.87 | -7000.00 | 1230.00 | 700100.00 | |
| Investments (time-of-study, £) | 12215.90 | 70727.69 | 0.00 | 0.00 | 1000000.00 | |
| *Categorical Variables* | Category | *n* |  |  |  | |
| Gender (4 missing, 3 other) | Female | 588 |  |  |  | |
|  | Male | 398 |  |  |  | |
| Relationship status (37 missing) | Married or relationship | 391 |  |  |  | |
|  | Separated | 51 |  |  |  | |
|  | Widowed | 9 |  |  |  | |
|  | Single | 505 |  |  |  | |
| Employment status (61 missing) | Employed | 714 |  |  |  | |
|  | Student | 263 | (172 student and employed) | | |  |
|  | Retired | 120 | (22 retired and student) | |  | |
|  | None | 29 |  |  |  | |
| Debt status (9 missing) | Has debt | 382 |  |  |  | |
|  | No debt | 602 |  |  |  | |
| *Note*. Life satisfaction was measured using the Satisfaction With Life Scale (Diener et al., 1985). Positive affect was measured with the Affect-Adjective Scale (Diener & Emmons, 1984). Both outcomes used a 7-point Likert scale.  Utilitarian spending statistics exclude one outlier participant who reported over £10,000,000,000 in purchases, mostly cash withdrawals. All other statistics include all participants.  Total *N* = 993. Employment status sample sizes sum to more than the total *N* because some participants reported multiple employment statuses (e.g., employed and student). | | | | | | |

| Table S6  *Correlation Matrix of Sample 2, Time 1 Variables* | | | | | | | | | | | |
| --- | --- | --- | --- | --- | --- | --- | --- | --- | --- | --- | --- |
|  |  | A | B | C | D | E | F | G | H | I | J |
| A | Life satisfaction |  |  |  |  |  |  |  |  |  |  |
| B | Positive affect | .68*** |  |  |  |  |  |  |  |  |  |
| C | Hedonic variety | .12*** | .20*** |  |  |  |  |  |  |  |  |
| D | Utilitarian variety | .08* | .06 | .39*** |  |  |  |  |  |  |  |
| E | log Total hedonic spending | .15*** | .17*** | .51*** | .42*** |  |  |  |  |  |  |
| F | log Total utilitarian spending | .12*** | .15*** | .40*** | .17*** | .61*** |  |  |  |  |  |
| G | Age | .02 | -.04 | -.06 | .30*** | .15*** | .15*** |  |  |  |  |
| H | log Income | .02 | .02 | .08* | .23*** | .19*** | .13*** | .19*** |  |  |  |
| I | log Liquid wealth | .10** | .03 | .00 | .09** | .16*** | .15*** | .20*** | .14*** |  |  |
| J | log Investments | .09** | .04 | -.02 | .12*** | .11*** | .11*** | .27*** | .16*** | .33*** |  |
| K | Debt status (1 = has debt) | .01 | .04 | .10** | .13*** | .16*** | .16*** | .04 | .10** | -.20*** | -.03 |
| *Note*. **p* < .05. ***p* < .01. ****p* < .001. *N* = 993. | | | | | | | | | | | |

| Table S7  *Preliminary Regression Models Predicting Positive Affect (Sample 2, Time 1)* | | | | | | | | | |
| --- | --- | --- | --- | --- | --- | --- | --- | --- | --- |
|  |  | Demographics only | | |  |  | Financial variables | | |
| Predictor |  | *B* (*SE*) |  | β |  |  | *B* (*SE*) |  | β |
| Intercept |  | 4.19 (0.20) |  | *** |  |  | 3.53 (0.61) |  | *** |
| Age | - | 0.01 (0.00) | - | .06 |  | - | 0.01 (0.00) | - | .08† |
| Female |  | 0.25 (0.08) |  | .10** |  |  | 0.28 (0.09) |  | .11** |
| Married |  | 0.47 (0.10) |  | .18*** |  |  | 0.44 (0.10) |  | .17*** |
| Separated | - | 0.09 (0.20) | - | .02 |  | - | 0.11 (0.20) | - | .02 |
| Widowed | - | 0.50 (0.44) | - | .04 |  | - | 0.51 (0.44) | - | .04 |
| Student |  | 0.15 (0.11) |  | .05 |  |  | 0.15 (0.11) |  | .05 |
| Employed |  | 0.07 (0.15) |  | .03 |  |  | 0.02 (0.15) |  | .01 |
| Retired | - | 0.18 (0.19) | - | .05 |  | - | 0.21 (0.19) | - | .05 |
| log Liquid wealth |  |  |  |  |  |  | 0.16 (0.15) |  | .04 |
| log Income |  |  |  |  |  |  | 0.01 (0.04) |  | .01 |
| log Investments |  |  |  |  |  |  | 0.04 (0.03) |  | .05 |
| Debt status (1 = has debt) |  |  |  |  |  |  | 0.10 (0.09) |  | .04 |
| *R*^2^ (adjusted *R*^2^) |  | .053 (.041) | | |  |  | .063 (.043) | | |
| Δ*R*^2^ |  |  |  |  |  |  | .010 |  |  |
| Model *F* (*df*_numerator_, *df*_denominator_) |  | 4.53 (12, 980) | | |  |  | 3.25 (20, 972) | | |
| *F*-change (*df*_numerator_, *df*_denominator_) |  |  | | |  |  | 1.32 (8, 972) | | |
| *Note*. †*p* < .10. **p* < .05. ***p* < .01. ****p* < .001.  Nominal demographic variables (e.g., employment) are dummy coded. Missingness dummy variables are not reported. | | | | | | | | | |

| Table S8  *Regression Models for Spending and Spending Variety Predicting Positive Affect (Sample 2, Time 1)* | | | | | | | | | | | | | | | |
| --- | --- | --- | --- | --- | --- | --- | --- | --- | --- | --- | --- | --- | --- | --- | --- |
|  |  | Model 1: Utilitarian spending and variety | | |  |  | Model 2: Hedonic spending | | |  |  | Model 3: Hedonic variety | | | |
| Predictor |  | *B* (*SE*) |  | β |  |  | *B* (*SE*) |  | β |  |  | *B* (*SE*) |  | β | *r*_semipartial_ |
| Intercept |  | 3.07 (0.62) |  | *** |  |  | 3.11 (0.62) |  | *** |  |  | 2.99 (0.62) |  | *** |  |
| Age | - | 0.01 (0.00) | - | .09* |  | - | 0.01 (0.00) | - | .09* |  | - | 0.01 (0.00) | - | .06 | .04 |
| Female |  | 0.25 (0.09) |  | .10** |  |  | 0.25 (0.09) |  | .09** |  |  | 0.22 (0.08) |  | .09** | .08 |
| Married |  | 0.41 (0.10) |  | .15*** |  |  | 0.40 (0.10) |  | .15*** |  |  | 0.42 (0.10) |  | .16*** | .13 |
| Separated | - | 0.09 (0.20) | - | .02 |  | - | 0.10 (0.20) | - | .02 |  | - | 0.11 (0.20) | - | .02 | .02 |
| Widowed | - | 0.55 (0.44) | - | .04 |  | - | 0.44 (0.44) | - | .03 |  | - | 0.39 (0.44) | - | .03 | .03 |
| Student |  | 0.13 (0.11) |  | .04 |  |  | 0.15 (0.11) |  | .05 |  |  | 0.15 (0.11) |  | .05 | .04 |
| Employed | - | 0.01 (0.15) | - | .00 |  | - | 0.00 (0.15) | - | .00 |  | - | 0.02 (0.15) | - | .01 | .00 |
| Retired | - | 0.22 (0.19) | - | .06 |  | - | 0.22 (0.19) | - | .06 |  | - | 0.21 (0.19) | - | .05 | .03 |
| log Liquid wealth |  | 0.08 (0.15) |  | .02 |  |  | 0.05 (0.15) |  | .01 |  |  | 0.08 (0.15) |  | .02 | .02 |
| log Income |  | 0.00 (0.04) |  | .00 |  | - | 0.00 (0.04) | - | .00 |  |  | 0.01 (0.04) |  | .00 | .00 |
| log Investments |  | 0.03 (0.03) |  | .04 |  |  | 0.03 (0.03) |  | .04 |  |  | 0.04 (0.03) |  | .05 | .04 |
| Debt status (1 = has debt) |  | 0.05 (0.09) |  | .02 |  |  | 0.04 (0.09) |  | .01 |  |  | 0.05 (0.09) |  | .02 | .02 |
| log Utilitarian spending (exact) |  | 0.22 (0.06) |  | .12*** |  |  | 0.11 (0.08) |  | .06 |  |  | 0.05 (0.08) |  | .03 | .02 |
| Utilitarian variety |  | 0.03 (0.07) |  | .01 |  | - | 0.04 (0.08) | - | .02 |  | - | 0.13 (0.08) | - | .06† | .05 |
| log Hedonic spending (exact) |  |  |  |  |  |  | 0.22 (0.09) |  | .11* |  |  | 0.12 (0.09) |  | .06 | .04 |
| Hedonic variety |  |  |  |  |  |  |  |  |  |  |  | 0.32 (0.08) |  | .15*** | .12 |
| *R*^2^ (adjusted *R*^2^) |  | .075 (.054) | | |  |  | .081 (.059) | | |  |  | .096 (.073) | | |  |
| Δ*R*^2^ |  | .012 |  |  |  |  | .006 |  |  |  |  | .015 |  |  |  |
| Model *F* (*df*_numerator_, *df*_denominator_) |  | 3.57 (22, 970) | | |  |  | 3.71 (23, 969) | | |  |  | 4.27 (24, 968) | | |  |
| *F*-change (*df*_numerator_, *df*_denominator_) |  | 6.43 (2, 970) | | |  |  | 6.38 (1, 969) | | |  |  | 15.82 (1, 968) | | |  |
| *Note*. †*p* < .10. **p* < .05. ***p* < .01. ****p* < .001.  Nominal demographic variables (e.g., employment) are dummy coded. Missingness dummy variables are not reported. | | | | | | | | | | | | | | | |

| Table S9  *Preliminary Regression Models Predicting Life Satisfaction (Sample 2, Time 1)* | | | | | | | | | |
| --- | --- | --- | --- | --- | --- | --- | --- | --- | --- |
|  |  | Demographics only | | |  |  | Financial variables | | |
| Predictor |  | *B* (*SE*) |  | β |  |  | *B* (*SE*) |  | β |
| Intercept |  | 3.81 (0.20) |  | *** |  |  | 2.45 (0.60) |  | *** |
| Age | - | 0.01 (0.00) | - | .07 |  | - | 0.01 (0.00) | - | .10* |
| Female |  | 0.23 (0.08) |  | .09** |  |  | 0.27 (0.08) |  | .10** |
| Sex other | - | 0.83 (0.48) | - | .05† |  | - | 0.69 (0.48) | - | .04 |
| Married |  | 0.81 (0.10) |  | .31*** |  |  | 0.80 (0.10) |  | .30*** |
| Separated |  | 0.11 (0.20) |  | .02 |  |  | 0.14 (0.20) |  | .02 |
| Widowed |  | 0.47 (0.43) |  | .03 |  |  | 0.45 (0.43) |  | .03 |
| Student |  | 0.27 (0.10) |  | .09* |  |  | 0.26 (0.10) |  | .09* |
| Employed |  | 0.12 (0.14) |  | .04 |  |  | 0.10 (0.15) |  | .03 |
| Retired | - | 0.05 (0.18) | - | .01 |  | - | 0.08 (0.19) | - | .02 |
| log Liquid wealth |  |  |  |  |  |  | 0.36 (0.15) |  | .08* |
| log Income |  |  |  |  |  | - | 0.01 (0.04) | - | .00 |
| log Investments |  |  |  |  |  |  | 0.04 (0.03) |  | .06† |
| Debt status (1 = has debt) |  |  |  |  |  |  | 0.04 (0.08) |  | .02 |
| *R*^2^ (adjusted *R*^2^) |  | .090 (.079) | | |  |  | .11 (.083) | | |
| Δ*R*^2^ |  |  |  |  |  |  | .012 |  |  |
| Model *F* (*df*_numerator_, *df*_denominator_) |  | 8.09 (12, 980) | | |  |  | 5.52 (20, 972) | | |
| *F*-change (*df*_numerator_, *df*_denominator_) |  |  | | |  |  | 1.60 (8, 972) | | |
| *Note*. †*p* < .10. **p* < .05. ***p* < .01. ****p* < .001.  Nominal demographic variables (e.g., employment) are dummy coded. Missingness dummy variables are not reported. | | | | | | | | | |

| Table S10  *Regression Models for Spending and Spending Variety Predicting Life Satisfaction (Sample 2, Time 1)* | | | | | | | | | | | | | | | |
| --- | --- | --- | --- | --- | --- | --- | --- | --- | --- | --- | --- | --- | --- | --- | --- |
|  |  | Model 1: Utilitarian spending and variety | | |  |  | Model 2: Hedonic spending | | |  |  | Model 3: Hedonic variety | | | |
| Predictor |  | *B* (*SE*) |  | β |  |  | *B* (*SE*) |  | β |  |  | *B* (*SE*) |  | β | *r*_semipartial_ |
| Intercept |  | 2.19 (0.61) |  | *** |  |  | 2.22 (0.61) |  | *** |  |  | 2.16 (0.61) |  | *** |  |
| Age | - | 0.01 (0.00) | - | .11* |  | - | 0.01 (0.00) | - | .10* |  | - | 0.01 (0.00) | - | .09* | .06 |
| Female |  | 0.26 (0.08) |  | .10** |  |  | 0.25 (0.08) |  | .10** |  |  | 0.24 (0.08) |  | .09** | .09 |
| Sex other | - | 0.67 (0.48) | - | .04 |  | - | 0.62 (0.48) | - | .04 |  | - | 0.64 (0.48) | - | .04 | .04 |
| Married |  | 0.78 (0.10) |  | .29*** |  |  | 0.77 (0.10) |  | .29*** |  |  | 0.78 (0.10) |  | .30*** | .24 |
| Separated |  | 0.15 (0.20) |  | .03 |  |  | 0.15 (0.20) |  | .02 |  |  | 0.14 (0.20) |  | .02 | .02 |
| Widowed |  | 0.42 (0.43) |  | .03 |  |  | 0.53 (0.43) |  | .04 |  |  | 0.55 (0.43) |  | .04 | .04 |
| Student |  | 0.25 (0.11) |  | .09* |  |  | 0.27 (0.11) |  | .09** |  |  | 0.27 (0.11) |  | .09** | .08 |
| Employed |  | 0.08 (0.15) |  | .03 |  |  | 0.08 (0.15) |  | .03 |  |  | 0.07 (0.15) |  | .03 | .02 |
| Retired | - | 0.08 (0.19) | - | .02 |  | - | 0.08 (0.18) | - | .02 |  | - | 0.07 (0.18) | - | .02 | .01 |
| log Liquid wealth |  | 0.31 (0.15) |  | .07* |  |  | 0.28 (0.15) |  | .07† |  |  | 0.30 (0.15) |  | .07* | .06 |
| log Income | - | 0.01 (0.04) | - | .01 |  | - | 0.02 (0.04) | - | .01 |  | - | 0.01 (0.04) | - | .01 | .01 |
| log Investments |  | 0.04 (0.03) |  | .05 |  |  | 0.04 (0.03) |  | .05 |  |  | 0.04 (0.03) |  | .06† | .05 |
| Debt status (1 = has debt) |  | 0.01 (0.09) |  | .00 |  |  | 0.00 (0.09) |  | .00 |  |  | 0.01 (0.09) |  | .00 | .00 |
| log Utilitarian spending (exact) |  | 0.12 (0.06) |  | .06* |  |  | 0.02 (0.08) |  | .01 |  | - | 0.01 (0.08) | - | .01 | .00 |
| Utilitarian variety |  | 0.03 (0.07) |  | .02 |  | - | 0.03 (0.08) | - | .02 |  | - | 0.08 (0.08) | - | .04 | .03 |
| log Hedonic spending (exact) |  |  |  |  |  |  | 0.21 (0.08) |  | .11* |  |  | 0.16 (0.09) |  | .08† | .06 |
| Hedonic variety |  |  |  |  |  |  |  |  |  |  |  | 0.15 (0.08) |  | .07† | .06 |
| *R*^2^ (adjusted *R*^2^) |  | .11 (.086) | | |  |  | .11 (.090) | | |  |  | .11 (.093) | | |  |
| Δ*R*^2^ |  | .026 |  |  |  |  | .006 |  |  |  |  | .003 |  |  |  |
| Model *F* (*df*_numerator_, *df*_denominator_) |  | 5.22 (22, 970) | | |  |  | 5.29 (23, 969) | | |  |  | 5.24 (24, 968) | | |  |
| *F*-change (*df*_numerator_, *df*_denominator_) |  | 2.18 (2, 970) | | |  |  | 6.16 (1, 969) | | |  |  | 3.68 (1, 968) | | |  |
| *Note*. †*p* < .10. **p* < .05. ***p* < .01. ****p* < .001.  Nominal demographic variables (e.g., employment) are dummy coded. Missingness dummy variables are not reported. | | | | | | | | | | | | | | | |

| Table S11  *Descriptive Statistics from Sample 3, Time 1* | | | | | | |
| --- | --- | --- | --- | --- | --- | --- |
|  | Mean | Standard deviation | Minimum | Median | Maximum | |
| Age in years | 32.91 | 11.60 | 18 | 30 | 77 | |
| Life satisfaction | 4.22 | 1.54 | 1.00 | 4.40 | 7.00 | |
| Positive affect | 4.39 | 1.41 | 1.00 | 4.50 | 7.00 | |
| Hedonic variety (externally-rated hedonic) | 1.29 | 0.69 | 0.00 | 1.37 | 3.51 | |
| Hedonic variety (self-reported hedonic, ver. 1) | 1.43 | 0.74 | 0.00 | 1.49 | 4.29 | |
| Hedonic variety (self-reported hedonic, ver. 2) | 1.78 | 0.73 | 0.00 | 1.89 | 4.29 | |
| Utilitarian variety (externally-rated utilitarian) | 1.62 | 0.69 | 0.00 | 1.66 | 4.30 | |
| Utilitarian variety (self-reported utilitarian, ver. 1) | 1.42 | 0.71 | 0.00 | 1.47 | 3.85 | |
| Utilitarian variety (self-reported utilitarian, ver. 2) | 1.70 | 0.71 | 0.00 | 1.76 | 4.31 | |
| Hedonic spending ($; externally-rated hedonic) | 3941.43 | 24852.69 | 0.00 | 1732.05 | 915656.51 | |
| Hedonic spending ($; self-reported hedonic, ver. 1) | 6427.64 | 30390.13 | 0.00 | 1853.00 | 915716.51 | |
| Hedonic spending ($; self-reported hedonic, ver. 2) | 10135.02 | 32809.91 | 0.00 | 3881.00 | 922207.09 | |
| Utilitarian spending ($; externally-rated utilitarian) | 29609.48 | 90676.97 | 0.00 | 16062.00 | 2929044.00 | |
| Utilitarian spending ($; self-reported utilitarian, ver. 1) | 22619.52 | 86741.38 | 0.00 | 11400.00 | 2929911.00 | |
| Utilitarian spending ($; self-reported utilitarian, ver. 2) | 26326.90 | 87606.64 | 0.00 | 14531.75 | 2930121.50 | |
| Income (annual, $) | 49785.66 | 168703.73 | 0.00 | 25000.00 | 4000000.00 | |
| Liquid wealth (monthly, $) | 14767.81 | 156848.36 | -800.00 | 1500.00 | 5500000.00 | |
| Investments (time-of-study, $) | 28858.57 | 105363.24 | 0.00 | 0.00 | 1400000.00 | |
| *Categorical Variables* | Category | *n* |  |  |  | |
| Gender (3 missing) | Female | 689 |  |  |  | |
|  | Male | 710 |  |  |  | |
| Relationship status (13 missing) | Married | 463 |  |  |  | |
|  | In relationship | 307 |  |  |  | |
|  | Separated | 66 |  |  |  | |
|  | Widowed | 15 |  |  |  | |
|  | Single | 538 |  |  |  | |
| Employment status (0 missing) | Employed | 973 |  |  |  | |
|  | Student | 462 | (291 student and employed) | | |  |
|  | Retired or not seeking | 161 | (43 not seeking and student) | | |  |
|  | None | 140 |  |  |  | |
| Debt status (10 missing) | Has debt | 744 |  |  |  | |
|  | No debt | 648 |  |  |  | |
| *Note*. Life satisfaction was measured using the Satisfaction With Life Scale (Diener et al., 1985). Positive affect was measured with the Affect-Adjective Scale (Diener & Emmons, 1984). Both outcomes used a 7-point Likert scale.  For self-reported hedonic vs. utilitarian variables, version 1 excludes categories rated as equally hedonic and utilitarian; version 2 allocates equally hedonic/utilitarian categories as 50% hedonic and utilitarian.  Total *N* = 1402. Employment status sample sizes sum to more than the total *N* because some participants reported multiple employment statuses (e.g., employed and student). | | | | | | |

| Table S12  *Correlation Matrix of Sample 3, Time 1 Variables* | | | | | | | | | | | |
| --- | --- | --- | --- | --- | --- | --- | --- | --- | --- | --- | --- |
|  |  | A | B | C | D | E | F | G | H | I |  |
| A | Life satisfaction |  |  |  |  |  |  |  |  |  |  |
| B | Positive affect | .74*** |  |  |  |  |  |  |  |  |  |
| C | Hedonic variety (externally-rated hedonic) | .12*** | .14*** |  |  |  |  |  |  |  |  |
| D | Hedonic variety (self-reported hedonic, ver. 1) | .18*** | .21*** | .81*** |  |  |  |  |  |  |  |
| E | Hedonic variety (self-reported hedonic, ver. 2) | .14*** | .18*** | .78*** | .83*** |  |  |  |  |  |  |
| F | Utilitarian variety (externally-rated utilitarian) | .20*** | .18*** | .47*** | .51*** | .63*** |  |  |  |  |  |
| G | Utilitarian variety (self-reported utilitarian, ver. 1) | .14*** | .10*** | .45*** | .39*** | .42*** | .84*** |  |  |  |  |
| H | Utilitarian variety (self-reported utilitarian, ver. 2) | .13*** | .11*** | .48*** | .39*** | .56*** | .91*** | .90*** |  |  |  |
| I | log Total hedonic spending (externally-rated hedonic) | .11*** | .14*** | .60*** | .54*** | .58*** | .48*** | .44*** | .49*** |  |  |
| J | log Total hedonic spending (self-reported hedonic, ver. 1) | .19*** | .21*** | .54*** | .63*** | .59*** | .48*** | .36*** | .38*** | .75*** |  |
| K | log Total hedonic spending (self-reported hedonic, ver. 2) | .20*** | .21*** | .49*** | .52*** | .53*** | .51*** | .38*** | .46*** | .78*** |  |
| L | log Total utilitarian spending (externally-rated utilitarian) | .14*** | .14*** | .41*** | .39*** | .42*** | .44*** | .43*** | .42*** | .65*** |  |
| M | log Total utilitarian spending (self-reported utilitarian, ver. 1) | .08** | .06* | .39*** | .33*** | .37*** | .45*** | .51*** | .46*** | .57*** |  |
| N | log Total utilitarian spending (self-reported utilitarian, ver. 2) | .09** | .08** | .40*** | .33*** | .38*** | .43*** | .48*** | .47*** | .62*** |  |
| O | Age | .05* | -.01 | .04 | .01 | .06* | .28*** | .30*** | .30*** | .14*** |  |
| P | log Income | .15*** | .13*** | .16*** | .19*** | .20*** | .30*** | .25*** | .26*** | .26*** |  |
| Q | log Liquid wealth | .22*** | .16*** | .15*** | .19*** | .15*** | .15*** | .11*** | .10*** | .19*** |  |
| R | log Investments | .25*** | .17*** | .15*** | .18*** | .15*** | .20*** | .17*** | .15*** | .20*** |  |
| S | Debt status (1 = has debt) | -.02 | -.02 | .09*** | .09*** | .11*** | .20*** | .20*** | .20*** | .13*** |  |

| Table S12 (continued) | | | | | | | | | | | |
| --- | --- | --- | --- | --- | --- | --- | --- | --- | --- | --- | --- |
|  |  | J | K | L | M | N | O | P | Q | R |  |
| J | log Total hedonic spending (self-reported hedonic, ver. 1) |  |  |  |  |  |  |  |  |  |  |
| K | log Total hedonic spending (self-reported hedonic, ver. 2) | .83*** |  |  |  |  |  |  |  |  |  |
| L | log Total utilitarian spending (externally-rated utilitarian) | .61*** | .75*** |  |  |  |  |  |  |  |  |
| M | log Total utilitarian spending (self-reported utilitarian, ver. 1) | .48*** | .53*** | .87*** |  |  |  |  |  |  |  |
| N | log Total utilitarian spending (self-reported utilitarian, ver. 2) | .50*** | .64*** | .92*** | .93*** |  |  |  |  |  |  |
| O | Age | .10*** | .15*** | .19*** | .18*** | .19*** |  |  |  |  |  |
| P | log Income | .28*** | .32*** | .35*** | .29*** | .30*** | .23*** |  |  |  |  |
| Q | log Liquid wealth | .25*** | .28*** | .26*** | .20*** | .22*** | .01 | .29*** |  |  |  |
| R | log Investments | .26*** | .27*** | .28*** | .22*** | .23*** | .21*** | .30*** | .45*** |  |  |
| S | Debt status (1 = has debt) | .12*** | .11*** | .15*** | .16*** | .15*** | .10*** | .15*** | -.12*** | .04 |  |
| *Note*. **p* < .05. ***p* < .01. ****p* < .001. *N* = 1402.  For self-reported hedonic vs. utilitarian variables, version 1 excludes categories rated as equally hedonic and utilitarian; version 2 allocates equally hedonic/utilitarian categories as 50% hedonic and utilitarian. | | | | | | | | | | | |

| Table S13  *Preliminary Regression Models Predicting Life Satisfaction (Sample 3, Time 1)* | | | | | | | | | |
| --- | --- | --- | --- | --- | --- | --- | --- | --- | --- |
|  |  | Demographics only | | |  |  | Financial variables | | |
| Predictor |  | *B* (*SE*) |  | β |  |  | *B* (*SE*) |  | β |
| Intercept |  | 3.34 (0.17) |  | .00*** |  |  | 2.33 (0.31) |  | .00*** |
| Age | - | 0.01 (0.00) | - | .04 |  | - | 0.01 (0.00) | - | .06† |
| Female | - | 0.14 (0.08) | - | .05† |  | - | 0.01 (0.08) | - | .00 |
| Married |  | 1.44 (0.10) |  | .44*** |  |  | 1.34 (0.10) |  | .41*** |
| In relationship, unmarried |  | 0.60 (0.10) |  | .16*** |  |  | 0.58 (0.10) |  | .16*** |
| Separated |  | 0.38 (0.20) |  | .05† |  |  | 0.52 (0.20) |  | .07** |
| Widowed |  | 0.49 (0.38) |  | .03 |  |  | 0.43 (0.37) |  | .03 |
| Student |  | 0.45 (0.09) |  | .14*** |  |  | 0.50 (0.09) |  | .15*** |
| Employed |  | 0.47 (0.10) |  | .14*** |  |  | 0.29 (0.11) |  | .09** |
| Retired |  | 0.29 (0.15) |  | .06† |  |  | 0.29 (0.15) |  | .06* |
| log Liquid wealth |  |  |  |  |  |  | 0.27 (0.08) |  | .10*** |
| log Income |  |  |  |  |  |  | 0.03 (0.04) |  | .03 |
| log Investments |  |  |  |  |  |  | 0.10 (0.02) |  | .14*** |
| Debt status (1 = has debt) |  |  |  |  |  | - | 0.16 (0.08) | - | .05* |
| *R*^2^ (adjusted *R*^2^) |  | .17 (.17) | | |  |  | .21 (.20) | | |
| Δ*R*^2^ |  |  | | |  |  | .040 | | |
| Model *F* (*df*_numerator_, *df*_denominator_) |  | 26.20 (11, 1388) | | |  |  | 19.57 (19, 1380) | | |
| *F*-change (*df*_numerator_, *df*_denominator_) |  |  | | |  |  | 8.83 (8, 1380) | | |
| *Note*. †*p* < .10. **p* < .05. ***p* < .01. ****p* < .001.  Nominal demographic variables (e.g., employment) are dummy coded. Missingness dummy variables are not reported. | | | | | | | | | |

| Table S14  *Regression Models for Spending and Spending Variety Predicting Life Satisfaction (Sample 3, Time 1; Externally-Rated Hedonic/Utilitarian Categories)* | | | | | | | | | | | | | | | |
| --- | --- | --- | --- | --- | --- | --- | --- | --- | --- | --- | --- | --- | --- | --- | --- |
|  |  | Model 1: Utilitarian spending and variety | | |  |  | Model 2: Hedonic spending | | |  |  | Model 3: Hedonic variety | | | |
| Predictor |  | *B* (*SE*) |  | β |  |  | *B* (*SE*) |  | β |  |  | *B* (*SE*) |  | β | *r*_semipartial_ |
| Intercept |  | 2.54 (0.33) |  | .00*** |  |  | 2.54 (0.33) |  | .00*** |  |  | 2.55 (0.33) |  | .00*** |  |
| Age | - | 0.01 (0.00) | - | .07* |  | - | 0.01 (0.00) | - | .07* |  | - | 0.01 (0.00) | - | .07* | .05 |
| Female |  | 0.00 (0.08) |  | .00 |  |  | 0.01 (0.08) |  | .00 |  | - | 0.00 (0.08) | - | .00 | .00 |
| Married |  | 1.30 (0.10) |  | .40*** |  |  | 1.30 (0.10) |  | .40*** |  |  | 1.30 (0.10) |  | .40*** | .30 |
| In relationship, unmarried |  | 0.58 (0.10) |  | .16*** |  |  | 0.59 (0.10) |  | .16*** |  |  | 0.58 (0.10) |  | .16*** | .14 |
| Separated |  | 0.53 (0.20) |  | .07** |  |  | 0.52 (0.20) |  | .07** |  |  | 0.52 (0.20) |  | .07** | .06 |
| Widowed |  | 0.46 (0.37) |  | .03 |  |  | 0.46 (0.37) |  | .03 |  |  | 0.48 (0.37) |  | .03 | .03 |
| Student |  | 0.50 (0.09) |  | .15*** |  |  | 0.50 (0.09) |  | .15*** |  |  | 0.50 (0.09) |  | .15*** | .13 |
| Employed |  | 0.27 (0.11) |  | .08* |  |  | 0.27 (0.11) |  | .08* |  |  | 0.27 (0.11) |  | .08* | .06 |
| Retired or not seeking employment |  | 0.29 (0.14) |  | .06* |  |  | 0.29 (0.14) |  | .06* |  |  | 0.29 (0.14) |  | .06* | .05 |
| log Liquid wealth |  | 0.28 (0.08) |  | .10*** |  |  | 0.28 (0.08) |  | .10*** |  |  | 0.28 (0.08) |  | .10*** | .09 |
| log Income |  | 0.03 (0.04) |  | .03 |  |  | 0.03 (0.04) |  | .03 |  |  | 0.03 (0.04) |  | .03 | .02 |
| log Investments |  | 0.11 (0.02) |  | .14*** |  |  | 0.11 (0.02) |  | .14*** |  |  | 0.11 (0.02) |  | .14*** | .12 |
| Debt status (1 = has debt) | - | 0.19 (0.08) | - | .06* |  | - | 0.19 (0.08) | - | .06* |  | - | 0.19 (0.08) | - | .06* | .06 |
| log Utilitarian spending (exact) | - | 0.13 (0.06) | - | .07* |  | - | 0.12 (0.07) | - | .06† |  | - | 0.12 (0.07) | - | .06† | .04 |
| Utilitarian variety |  | 0.24 (0.06) |  | .11*** |  |  | 0.24 (0.07) |  | .11*** |  |  | 0.22 (0.07) |  | .10** | .08 |
| log Hedonic spending (exact) |  |  |  |  |  | - | 0.02 (0.06) | - | .01 |  | - | 0.05 (0.06) | - | .03 | .02 |
| Hedonic variety |  |  |  |  |  |  |  |  |  |  |  | 0.08 (0.07) |  | .04 | .03 |
| *R*^2^ (adjusted *R*^2^) |  | .22 (.21) | | |  |  | .22 (.21) | | |  |  | .22 (.21) | | |  |
| Δ*R*^2^ |  | .009 | | |  |  | .000 | | |  |  | .001 | | |  |
| Model *F* (*df*_numerator_, *df*_denominator_) |  | 18.65 (21, 1378) | | |  |  | 17.80 (22, 1377) | | |  |  | 17.08 (23, 1376) | | |  |
| *F*-change (*df*_numerator_, *df*_denominator_) |  | 8.03 (2, 1378) | | |  |  | 0.10 (1, 1377) | | |  |  | 1.25 (1, 1376) | | |  |
| *Note*. †*p* < .10. **p* < .05. ***p* < .01. ****p* < .001.  Nominal demographic variables (e.g., employment) are dummy coded. Missingness dummy variables are not reported. | | | | | | | | | | | | | | | |

| Table S15  *Regression Models for Spending and Spending Variety Predicting Positive Affect (Sample 3, Time 1; Self-Reported Hedonic/Utilitarian Categories, Equally Hedonic/Utilitarian Categories Allocated 50% Hedonic/50% Utilitarian)* | | | | | | | | | | | | | | | |
| --- | --- | --- | --- | --- | --- | --- | --- | --- | --- | --- | --- | --- | --- | --- | --- |
|  |  | Model 1: Utilitarian spending and variety | | |  |  | Model 2: Hedonic spending | | |  |  | Model 3: Hedonic variety | | | |
| Predictor |  | *B* (*SE*) |  | β |  |  | *B* (*SE*) |  | β |  |  | *B* (*SE*) |  | β | *r*_semipartial_ |
| Intercept |  | 4.42 (0.33) |  | .00*** |  |  | 4.20 (0.33) |  | .00*** |  |  | 4.20 (0.33) |  | .00*** |  |
| Age | - | 0.01 (0.00) | - | .10** |  | - | 0.01 (0.00) | - | .09** |  | - | 0.01 (0.00) | - | .09** | .06 |
| Female | - | 0.07 (0.07) | - | .02 |  | - | 0.08 (0.07) | - | .03 |  | - | 0.09 (0.07) | - | .03 | .03 |
| Married |  | 1.05 (0.10) |  | .35*** |  |  | 1.01 (0.10) |  | .34*** |  |  | 1.02 (0.10) |  | .34*** | .26 |
| In relationship, unmarried |  | 0.65 (0.10) |  | .19*** |  |  | 0.63 (0.10) |  | .18*** |  |  | 0.61 (0.10) |  | .18*** | .16 |
| Separated |  | 0.40 (0.19) |  | .06* |  |  | 0.43 (0.19) |  | .07* |  |  | 0.44 (0.19) |  | .07* | .06 |
| Widowed |  | 0.44 (0.36) |  | .03 |  |  | 0.41 (0.35) |  | .03 |  |  | 0.41 (0.35) |  | .03 | .03 |
| Student |  | 0.44 (0.09) |  | .15*** |  |  | 0.43 (0.08) |  | .14*** |  |  | 0.42 (0.08) |  | .14*** | .12 |
| Employed |  | 0.12 (0.10) |  | .04 |  |  | 0.10 (0.10) |  | .03 |  |  | 0.10 (0.10) |  | .03 | .02 |
| Retired or not seeking employment |  | 0.10 (0.14) |  | .02 |  |  | 0.11 (0.14) |  | .02 |  |  | 0.11 (0.14) |  | .03 | .02 |
| log Liquid wealth |  | 0.14 (0.07) |  | .06† |  |  | 0.09 (0.07) |  | .04 |  |  | 0.09 (0.07) |  | .04 | .03 |
| log Income |  | 0.06 (0.03) |  | .06† |  |  | 0.05 (0.03) |  | .05 |  |  | 0.05 (0.03) |  | .05 | .04 |
| log Investments |  | 0.07 (0.02) |  | .10** |  |  | 0.06 (0.02) |  | .09** |  |  | 0.06 (0.02) |  | .09** | .07 |
| Debt status (1 = has debt) | - | 0.15 (0.07) | - | .05* |  | - | 0.14 (0.07) | - | .05† |  | - | 0.14 (0.07) | - | .05† | .05 |
| log Utilitarian spending (exact) | - | 0.08 (0.05) | - | .05 |  | - | 0.22 (0.06) | - | .13*** |  | - | 0.22 (0.06) | - | .13*** | .09 |
| Utilitarian variety |  | 0.14 (0.06) |  | .07* |  |  | 0.08 (0.06) |  | .04 |  |  | 0.00 (0.07) |  | .00 | .00 |
| log Hedonic spending (exact) |  |  |  |  |  |  | 0.31 (0.06) |  | .18*** |  |  | 0.26 (0.06) |  | .15*** | .10 |
| Hedonic variety |  |  |  |  |  |  |  |  |  |  |  | 0.16 (0.06) |  | .08** | .06 |
| *R*^2^ (adjusted *R*^2^) |  | .15 (.14) | | |  |  | .17 (.16) | | |  |  | .17 (.16) | | |  |
| Δ*R*^2^ |  | .004 | | |  |  | .016 | | |  |  | .004 | | |  |
| Model *F* (*df*_numerator_, *df*_denominator_) |  | 11.84 (21, 1378) | | |  |  | 12.70 (22, 1377) | | |  |  | 12.49 (23, 1376) | | |  |
| *F*-change (*df*_numerator_, *df*_denominator_) |  | 3.24 (2, 1378) | | |  |  | 26.27 (1, 1377) | | |  |  | 10.71 (1, 1376) | | |  |
| *Note*. †*p* < .10. **p* < .05. ***p* < .01. ****p* < .001.  Nominal demographic variables (e.g., employment) are dummy coded. Missingness dummy variables are not reported. | | | | | | | | | | | | | | | |

| Table S16  *Regression Models for Spending and Spending Variety Predicting Positive Affect (Sample 3, Time 1; Self-Reported Hedonic/Utilitarian Categories, Excluding Equally Hedonic/Utilitarian Categories)* | | | | | | | | | | | | | | | |
| --- | --- | --- | --- | --- | --- | --- | --- | --- | --- | --- | --- | --- | --- | --- | --- |
|  |  | Model 1: Utilitarian spending and variety | | |  |  | Model 2: Hedonic spending | | |  |  | Model 3: Hedonic variety | | | |
| Predictor |  | *B* (*SE*) |  | β |  |  | *B* (*SE*) |  | β |  |  | *B* (*SE*) |  | β | *r*_semipartial_ |
| Intercept |  | 4.44 (0.33) |  | .00*** |  |  | 4.25 (0.33) |  | .00*** |  |  | 4.27 (0.33) |  | .00*** |  |
| Age | - | 0.01 (0.00) | - | .09** |  | - | 0.01 (0.00) | - | .09** |  | - | 0.01 (0.00) | - | .08* | .06 |
| Female | - | 0.06 (0.07) | - | .02 |  | - | 0.07 (0.07) | - | .02 |  | - | 0.07 (0.07) | - | .03 | .02 |
| Married |  | 1.06 (0.10) |  | .35*** |  |  | 1.01 (0.10) |  | .34*** |  |  | 1.01 (0.10) |  | .34*** | .26 |
| In relationship, unmarried |  | 0.66 (0.10) |  | .19*** |  |  | 0.63 (0.10) |  | .18*** |  |  | 0.61 (0.10) |  | .18*** | .16 |
| Separated |  | 0.39 (0.19) |  | .06* |  |  | 0.43 (0.19) |  | .06* |  |  | 0.42 (0.19) |  | .06* | .06 |
| Widowed |  | 0.45 (0.36) |  | .03 |  |  | 0.40 (0.35) |  | .03 |  |  | 0.44 (0.35) |  | .03 | .03 |
| Student |  | 0.44 (0.09) |  | .15*** |  |  | 0.43 (0.08) |  | .14*** |  |  | 0.42 (0.08) |  | .14*** | .12 |
| Employed |  | 0.13 (0.10) |  | .04 |  |  | 0.12 (0.10) |  | .04 |  |  | 0.12 (0.10) |  | .04 | .03 |
| Retired or not seeking employment |  | 0.12 (0.14) |  | .03 |  |  | 0.13 (0.14) |  | .03 |  |  | 0.13 (0.14) |  | .03 | .02 |
| log Liquid wealth |  | 0.14 (0.07) |  | .06* |  |  | 0.11 (0.07) |  | .04 |  |  | 0.10 (0.07) |  | .04 | .03 |
| log Income |  | 0.07 (0.03) |  | .06* |  |  | 0.06 (0.03) |  | .05† |  |  | 0.06 (0.03) |  | .05† | .04 |
| log Investments |  | 0.07 (0.02) |  | .10** |  |  | 0.06 (0.02) |  | .09** |  |  | 0.06 (0.02) |  | .08** | .07 |
| Debt status (1 = has debt) | - | 0.14 (0.07) | - | .05† |  | - | 0.15 (0.07) | - | .05* |  | - | 0.15 (0.07) | - | .05* | .05 |
| log Utilitarian spending (exact) | - | 0.09 (0.05) | - | .06* |  | - | 0.16 (0.05) | - | .11*** |  | - | 0.15 (0.05) | - | .10** | .08 |
| Utilitarian variety |  | 0.12 (0.06) |  | .06* |  |  | 0.09 (0.06) |  | .04 |  |  | 0.03 (0.06) |  | .02 | .01 |
| log Hedonic spending (exact) |  |  |  |  |  |  | 0.22 (0.04) |  | .14*** |  |  | 0.13 (0.05) |  | .09* | .06 |
| Hedonic variety |  |  |  |  |  |  |  |  |  |  |  | 0.19 (0.06) |  | .10** | .07 |
| *R*^2^ (adjusted *R*^2^) |  | .15 (.14) | | |  |  | .17 (.15) | | |  |  | .17 (.16) | | |  |
| Δ*R*^2^ |  | .004 | | |  |  | .014 | | |  |  | .005 | | |  |
| Model *F* (*df*_numerator_, *df*_denominator_) |  | 11.80 (21, 1378) | | |  |  | 12.49 (22, 1377) | | |  |  | 12.41 (23, 1376) | | |  |
| *F*-change (*df*_numerator_, *df*_denominator_) |  | 2.86 (2, 1378) | | |  |  | 23.02 (1, 1377) | | |  |  | 9.06 (1, 1376) | | |  |
| *Note*. †*p* < .10. **p* < .05. ***p* < .01. ****p* < .001.  Nominal demographic variables (e.g., employment) are dummy coded. Missingness dummy variables are not reported. | | | | | | | | | | | | | | | |

| Table S17  *Preliminary Regression Models Predicting Life Satisfaction (Sample 3, Time 1)* | | | | | | | | | |
| --- | --- | --- | --- | --- | --- | --- | --- | --- | --- |
|  |  | Model 1: Demographics only | | |  |  | Model 2: Financial variables | | |
| Predictor |  | *B* (*SE*) |  | β |  |  | *B* (*SE*) |  | β |
| Intercept |  | 3.34 (0.17) |  | .00*** |  |  | 2.33 (0.31) |  | .00*** |
| Age | - | 0.01 (0.00) | - | .04 |  | - | 0.01 (0.00) | - | .06† |
| Female | - | 0.14 (0.08) | - | .05† |  | - | 0.01 (0.08) | - | .00 |
| Married |  | 1.44 (0.10) |  | .44*** |  |  | 1.34 (0.10) |  | .41*** |
| In relationship, unmarried |  | 0.60 (0.10) |  | .16*** |  |  | 0.58 (0.10) |  | .16*** |
| Separated |  | 0.38 (0.20) |  | .05† |  |  | 0.52 (0.20) |  | .07** |
| Widowed |  | 0.49 (0.38) |  | .03 |  |  | 0.43 (0.37) |  | .03 |
| Student |  | 0.45 (0.09) |  | .14*** |  |  | 0.50 (0.09) |  | .15*** |
| Employed |  | 0.47 (0.10) |  | .14*** |  |  | 0.29 (0.11) |  | .09** |
| Retired |  | 0.29 (0.15) |  | .06† |  |  | 0.29 (0.15) |  | .06* |
| log Liquid wealth |  |  |  |  |  |  | 0.27 (0.08) |  | .10*** |
| log Income |  |  |  |  |  |  | 0.03 (0.04) |  | .03 |
| log Investments |  |  |  |  |  |  | 0.10 (0.02) |  | .14*** |
| Debt status (1 = has debt) |  |  |  |  |  | - | 0.16 (0.08) | - | .05* |
| *R*^2^ (adjusted *R*^2^) |  | .17 (.17) | | |  |  | .21 (.20) | | |
| Δ*R*^2^ |  |  | | |  |  | .040 | | |
| Model *F* (*df*_numerator_, *df*_denominator_) |  | 26.20 (11, 1388) | | |  |  | 19.57 (19, 1380) | | |
| *F*-change (*df*_numerator_, *df*_denominator_) |  |  | | |  |  | 8.83 (8, 1380) | | |
| *Note*. †*p* < .10. **p* < .05. ***p* < .01. ****p* < .001.  Nominal demographic variables (e.g., employment) are dummy coded. Missingness dummy variables are not reported. | | | | | | | | | |

| Table S18  *Regression Models for Spending and Spending Variety Predicting Life Satisfaction (Sample 3, Time 1; Externally-Rated Hedonic/Utilitarian Categories)* | | | | | | | | | | | | | | | |
| --- | --- | --- | --- | --- | --- | --- | --- | --- | --- | --- | --- | --- | --- | --- | --- |
|  |  | Model 1: Utilitarian spending and variety | | |  |  | Model 2: Hedonic spending | | |  |  | Model 3: Hedonic variety | | | |
| Predictor |  | *B* (*SE*) |  | β |  |  | *B* (*SE*) |  | β |  |  | *B* (*SE*) |  | β | *r*_semipartial_ |
| Intercept |  | 2.54 (0.33) |  | .00*** |  |  | 2.54 (0.33) |  | .00*** |  |  | 2.55 (0.33) |  | .00*** |  |
| Age | - | 0.01 (0.00) | - | .07* |  | - | 0.01 (0.00) | - | .07* |  | - | 0.01 (0.00) | - | .07* | .05 |
| Female |  | 0.00 (0.08) |  | .00 |  |  | 0.01 (0.08) |  | .00 |  | - | 0.00 (0.08) | - | .00 | .00 |
| Married |  | 1.30 (0.10) |  | .40*** |  |  | 1.30 (0.10) |  | .40*** |  |  | 1.30 (0.10) |  | .40*** | .30 |
| In relationship, unmarried |  | 0.58 (0.10) |  | .16*** |  |  | 0.59 (0.10) |  | .16*** |  |  | 0.58 (0.10) |  | .16*** | .14 |
| Separated |  | 0.53 (0.20) |  | .07** |  |  | 0.52 (0.20) |  | .07** |  |  | 0.52 (0.20) |  | .07** | .06 |
| Widowed |  | 0.46 (0.37) |  | .03 |  |  | 0.46 (0.37) |  | .03 |  |  | 0.48 (0.37) |  | .03 | .03 |
| Student |  | 0.50 (0.09) |  | .15*** |  |  | 0.50 (0.09) |  | .15*** |  |  | 0.50 (0.09) |  | .15*** | .13 |
| Employed |  | 0.27 (0.11) |  | .08* |  |  | 0.27 (0.11) |  | .08* |  |  | 0.27 (0.11) |  | .08* | .06 |
| Retired or not seeking employment |  | 0.29 (0.14) |  | .06* |  |  | 0.29 (0.14) |  | .06* |  |  | 0.29 (0.14) |  | .06* | .05 |
| log Liquid wealth |  | 0.28 (0.08) |  | .10*** |  |  | 0.28 (0.08) |  | .10*** |  |  | 0.28 (0.08) |  | .10*** | .09 |
| log Income |  | 0.03 (0.04) |  | .03 |  |  | 0.03 (0.04) |  | .03 |  |  | 0.03 (0.04) |  | .03 | .02 |
| log Investments |  | 0.11 (0.02) |  | .14*** |  |  | 0.11 (0.02) |  | .14*** |  |  | 0.11 (0.02) |  | .14*** | .12 |
| Debt status (1 = has debt) | - | 0.19 (0.08) | - | .06* |  | - | 0.19 (0.08) | - | .06* |  | - | 0.19 (0.08) | - | .06* | .06 |
| log Utilitarian spending (exact) | - | 0.13 (0.06) | - | .07* |  | - | 0.12 (0.07) | - | .06† |  | - | 0.12 (0.07) | - | .06† | .04 |
| Utilitarian variety |  | 0.24 (0.06) |  | .11*** |  |  | 0.24 (0.07) |  | .11*** |  |  | 0.22 (0.07) |  | .10** | .08 |
| log Hedonic spending (exact) |  |  |  |  |  | - | 0.02 (0.06) | - | .01 |  | - | 0.05 (0.06) | - | .03 | .02 |
| Hedonic variety |  |  |  |  |  |  |  |  |  |  |  | 0.08 (0.07) |  | .04 | .03 |
| *R*^2^ (adjusted *R*^2^) |  | .22 (.21) | | |  |  | .22 (.21) | | |  |  | .22 (.21) | | |  |
| Δ*R*^2^ |  | .009 | | |  |  | .000 | | |  |  | .001 | | |  |
| Model *F* (*df*_numerator_, *df*_denominator_) |  | 18.65 (21, 1378) | | |  |  | 17.80 (22, 1377) | | |  |  | 17.08 (23, 1376) | | |  |
| *F*-change (*df*_numerator_, *df*_denominator_) |  | 8.03 (2, 1378) | | |  |  | 0.10 (1, 1377) | | |  |  | 1.25 (1, 1376) | | |  |
| *Note*. †*p* < .10. **p* < .05. ***p* < .01. ****p* < .001.  Nominal demographic variables (e.g., employment) are dummy coded. Missingness dummy variables are not reported. | | | | | | | | | | | | | | | |

| Table S19  *Regression Models for Spending and Spending Variety Predicting Life Satisfaction (Sample 3, Time 1; Self-Reported Hedonic/Utilitarian Categories, Equally Hedonic/Utilitarian Categories Allocated 50% Hedonic/50% Utilitarian)* | | | | | | | | | | | | | | | |
| --- | --- | --- | --- | --- | --- | --- | --- | --- | --- | --- | --- | --- | --- | --- | --- |
|  |  | Model 1: Utilitarian spending and variety | | |  |  | Model 2: Hedonic spending | | |  |  | Model 3: Hedonic variety | | | |
| Predictor |  | *B* (*SE*) |  | β |  |  | *B* (*SE*) |  | β |  |  | *B* (*SE*) |  | β | *r*_semipartial_ |
| Intercept |  | 2.60 (0.32) |  | .00*** |  |  | 2.48 (0.33) |  | .00*** |  |  | 2.48 (0.33) |  | .00*** |  |
| Age | - | 0.01 (0.00) | - | .07* |  | - | 0.01 (0.00) | - | .06* |  | - | 0.01 (0.00) | - | .06† | .05 |
| Female |  | 0.00 (0.08) |  | .00 |  | - | 0.01 (0.08) | - | .00 |  | - | 0.01 (0.08) | - | .00 | .00 |
| Married |  | 1.34 (0.10) |  | .41*** |  |  | 1.31 (0.10) |  | .40*** |  |  | 1.31 (0.10) |  | .40*** | .31 |
| In relationship, unmarried |  | 0.60 (0.10) |  | .16*** |  |  | 0.58 (0.10) |  | .16*** |  |  | 0.58 (0.10) |  | .15*** | .14 |
| Separated |  | 0.53 (0.20) |  | .07** |  |  | 0.56 (0.20) |  | .08** |  |  | 0.56 (0.20) |  | .08** | .07 |
| Widowed |  | 0.46 (0.37) |  | .03 |  |  | 0.43 (0.37) |  | .03 |  |  | 0.43 (0.37) |  | .03 | .03 |
| Student |  | 0.51 (0.09) |  | .16*** |  |  | 0.51 (0.09) |  | .15*** |  |  | 0.50 (0.09) |  | .15*** | .13 |
| Employed |  | 0.29 (0.11) |  | .09** |  |  | 0.27 (0.11) |  | .08* |  |  | 0.27 (0.11) |  | .08* | .06 |
| Retired or not seeking employment |  | 0.30 (0.15) |  | .06* |  |  | 0.30 (0.14) |  | .06* |  |  | 0.30 (0.14) |  | .06* | .05 |
| log Liquid wealth |  | 0.30 (0.08) |  | .11*** |  |  | 0.27 (0.08) |  | .10*** |  |  | 0.27 (0.08) |  | .10*** | .08 |
| log Income |  | 0.04 (0.04) |  | .03 |  |  | 0.03 (0.04) |  | .03 |  |  | 0.03 (0.04) |  | .03 | .02 |
| log Investments |  | 0.11 (0.02) |  | .14*** |  |  | 0.10 (0.02) |  | .14*** |  |  | 0.10 (0.02) |  | .14*** | .11 |
| Debt status (1 = has debt) | - | 0.16 (0.08) | - | .05* |  | - | 0.16 (0.08) | - | .05* |  | - | 0.16 (0.08) | - | .05* | .05 |
| log Utilitarian spending (exact) | - | 0.16 (0.05) | - | .09** |  | - | 0.26 (0.06) | - | .14*** |  | - | 0.25 (0.06) | - | .14*** | .10 |
| Utilitarian variety |  | 0.14 (0.06) |  | .07* |  |  | 0.10 (0.06) |  | .05 |  |  | 0.09 (0.07) |  | .04 | .03 |
| log Hedonic spending (exact) |  |  |  |  |  |  | 0.21 (0.06) |  | .11** |  |  | 0.20 (0.07) |  | .10** | .07 |
| Hedonic variety |  |  |  |  |  |  |  |  |  |  |  | 0.03 (0.07) |  | .02 | .01 |
| *R*^2^ (adjusted *R*^2^) |  | .22 (.21) | | |  |  | .22 (.21) | | |  |  | .22 (.21) | | |  |
| Δ*R*^2^ |  | .006 | | |  |  | .006 | | |  |  | .000 | | |  |
| Model *F* (*df*_numerator_, *df*_denominator_) |  | 18.34 (21, 1378) | | |  |  | 18.11 (22, 1377) | | |  |  | 17.33 (23, 1376) | | |  |
| *F*-change (*df*_numerator_, *df*_denominator_) |  | 5.50 (2, 1378) | | |  |  | 10.53 (1, 1377) | | |  |  | 0.25 (1, 1376) | | |  |
| *Note*. †*p* < .10. **p* < .05. ***p* < .01. ****p* < .001.  Nominal demographic variables (e.g., employment) are dummy coded. Missingness dummy variables are not reported. | | | | | | | | | | | | | | | |

| Table S20  *Regression Models for Spending and Spending Variety Predicting Life Satisfaction (Sample 3, Time 1; Self-Reported Hedonic/Utilitarian Categories, Excluding Equally Hedonic/Utilitarian Categories)* | | | | | | | | | | | | | | | |
| --- | --- | --- | --- | --- | --- | --- | --- | --- | --- | --- | --- | --- | --- | --- | --- |
|  |  | Model 1: Utilitarian spending and variety | | |  |  | Model 2: Hedonic spending | | |  |  | Model 3: Hedonic variety | | | |
| Predictor |  | *B* (*SE*) |  | β |  |  | *B* (*SE*) |  | β |  |  | *B* (*SE*) |  | β | *r*_semipartial_ |
| Intercept |  | 2.55 (0.32) |  | .00*** |  |  | 2.46 (0.32) |  | .00*** |  |  | 2.48 (0.32) |  | .00*** |  |
| Age | - | 0.01 (0.00) | - | .07* |  | - | 0.01 (0.00) | - | .06* |  | - | 0.01 (0.00) | - | .06† | .04 |
| Female |  | 0.01 (0.08) |  | .00 |  |  | 0.00 (0.08) |  | .00 |  | - | 0.00 (0.08) | - | .00 | .00 |
| Married |  | 1.33 (0.10) |  | .41*** |  |  | 1.30 (0.10) |  | .40*** |  |  | 1.30 (0.10) |  | .40*** | .30 |
| In relationship, unmarried |  | 0.60 (0.10) |  | .16*** |  |  | 0.58 (0.10) |  | .16*** |  |  | 0.57 (0.10) |  | .15*** | .13 |
| Separated |  | 0.52 (0.20) |  | .07** |  |  | 0.54 (0.20) |  | .07** |  |  | 0.54 (0.20) |  | .07** | .07 |
| Widowed |  | 0.47 (0.37) |  | .03 |  |  | 0.44 (0.37) |  | .03 |  |  | 0.46 (0.37) |  | .03 | .03 |
| Student |  | 0.51 (0.09) |  | .16*** |  |  | 0.51 (0.09) |  | .15*** |  |  | 0.50 (0.09) |  | .15*** | .13 |
| Employed |  | 0.29 (0.11) |  | .09** |  |  | 0.29 (0.11) |  | .09** |  |  | 0.28 (0.11) |  | .08** | .06 |
| Retired or not seeking employment |  | 0.31 (0.15) |  | .06* |  |  | 0.31 (0.14) |  | .07* |  |  | 0.32 (0.14) |  | .07* | .05 |
| log Liquid wealth |  | 0.30 (0.08) |  | .11*** |  |  | 0.27 (0.08) |  | .10*** |  |  | 0.27 (0.08) |  | .10*** | .08 |
| log Income |  | 0.04 (0.04) |  | .04 |  |  | 0.04 (0.04) |  | .03 |  |  | 0.04 (0.04) |  | .03 | .02 |
| log Investments |  | 0.11 (0.02) |  | .14*** |  |  | 0.10 (0.02) |  | .14*** |  |  | 0.10 (0.02) |  | .14*** | .11 |
| Debt status (1 = has debt) | - | 0.16 (0.08) | - | .05* |  | - | 0.17 (0.08) | - | .05* |  | - | 0.17 (0.08) | - | .05* | .05 |
| log Utilitarian spending (exact) | - | 0.15 (0.05) | - | .09** |  | - | 0.19 (0.05) | - | .12*** |  | - | 0.18 (0.05) | - | .11*** | .09 |
| Utilitarian variety |  | 0.15 (0.06) |  | .07* |  |  | 0.13 (0.06) |  | .06* |  |  | 0.10 (0.07) |  | .04 | .03 |
| log Hedonic spending (exact) |  |  |  |  |  |  | 0.13 (0.05) |  | .08** |  |  | 0.08 (0.06) |  | .05 | .03 |
| Hedonic variety |  |  |  |  |  |  |  |  |  |  |  | 0.11 (0.07) |  | .05† | .04 |
| *R*^2^ (adjusted *R*^2^) |  | .22 (.21) | | |  |  | .22 (.21) | | |  |  | .22 (.21) | | |  |
| Δ*R*^2^ |  | .006 | | |  |  | .004 | | |  |  | .002 | | |  |
| Model *F* (*df*_numerator_, *df*_denominator_) |  | 18.35 (21, 1378) | | |  |  | 17.94 (22, 1377) | | |  |  | 17.31 (23, 1376) | | |  |
| *F*-change (*df*_numerator_, *df*_denominator_) |  | 5.57 (2, 1378) | | |  |  | 7.53 (1, 1377) | | |  |  | 2.87 (1, 1376) | | |  |
| *Note*. †*p* < .10. **p* < .05. ***p* < .01. ****p* < .001.  Nominal demographic variables (e.g., employment) are dummy coded. Missingness dummy variables are not reported. | | | | | | | | | | | | | | | |

| Table S21  *Descriptive Statistics from Sample 2, Time 2* | | | | | | |
| --- | --- | --- | --- | --- | --- | --- |
|  | Mean | Standard deviation | Minimum | Median | Maximum | |
| Age in years | 37.09 | 12.29 | 18 | 35 | 73 | |
| Life satisfaction | 4.16 | 1.33 | 1.00 | 4.40 | 7.00 | |
| Positive affect | 4.07 | 1.29 | 1.00 | 4.00 | 7.00 | |
| Hedonic variety | 1.47 | 0.66 | 0.00 | 1.58 | 2.70 | |
| Utilitarian variety | 1.76 | 0.61 | 0.00 | 1.81 | 3.22 | |
| Hedonic spending (prior 12 months, £) | 878.21 | 1174.62 | 0.00 | 577.50 | 12380.00 | |
| Utilitarian spending (prior 12 months, £) | 8765.00 | 28584.34 | 1.50 | 3944.50 | 502812.00 | |
| Income (annual, £) | 21022.15 | 42783.99 | 0.00 | 15000.00 | 850000.00 | |
| Liquid wealth (monthly, £) | 11055.45 | 43612.44 | -7495.00 | 1325.00 | 698500.00 | |
| Investments (time-of-study, £) | 15582.91 | 96620.77 | 0.00 | 0.00 | 1500000.00 | |
| *Categorical Variables* | Category | *n* |  |  |  | |
| Gender (1 other) | Female | 381 |  |  |  | |
|  | Male | 250 |  |  |  | |
| Relationship status (21 missing) | Married or relationship | 269 |  |  |  | |
|  | Separated | 38 |  |  |  | |
|  | Widowed | 5 |  |  |  | |
|  | Single | 299 |  |  |  | |
| Employment status (34 missing) | Employed | 460 |  |  |  | |
|  | Student | 148 | (98 student and employed) | | |  |
|  | Retired | 86 | (14 retired and student) | |  | |
|  | None | 16 |  |  |  | |
| Debt status (0 missing) | Has debt | 230 |  |  |  | |
|  | No debt | 402 |  |  |  | |
| *Note*. Life satisfaction was measured using the Satisfaction With Life Scale (Diener et al., 1985). Positive affect was measured with the Affect-Adjective Scale (Diener & Emmons, 1984). Both outcomes used a 7-point Likert scale.  All values are Time 2 statistics. Total *N* = 632. Employment status sample sizes sum to more than the total *N* because some participants reported multiple employment statuses (e.g., employed and student). | | | | | | |

| Table S22  *Correlation Matrix of Sample 2, Time 2 Variables* | | | | | | | | | | | | |
| --- | --- | --- | --- | --- | --- | --- | --- | --- | --- | --- | --- | --- |
|  |  | A | B | C | D | E | F | G | H | I | J | K |
| A | Life satisfaction (Time 1) |  |  |  |  |  |  |  |  |  |  |  |
| B | Life satisfaction (Time 2) | .84*** |  |  |  |  |  |  |  |  |  |  |
| C | Positive affect (Time 1) | .68*** | .63*** |  |  |  |  |  |  |  |  |  |
| D | Positive affect (Time 2) | .61*** | .69*** | .75*** |  |  |  |  |  |  |  |  |
| E | Hedonic variety | .08* | .10** | .13** | .15*** |  |  |  |  |  |  |  |
| F | Utilitarian variety | .02 | .00 | .06 | .04 | .48*** |  |  |  |  |  |  |
| G | log Total hedonic spending | .10* | .08* | .12** | .12** | .58*** | .49*** |  |  |  |  |  |
| H | log Total utilitarian spending | .19*** | .17*** | .08* | .13** | .27*** | .19*** | .39*** |  |  |  |  |
| I | Age | .02 | .00 | -.05 | -.03 | -.04 | .25*** | .10** | .18*** |  |  |  |
| J | log Income | .03 | .03 | -.01 | .00 | .04 | .20*** | .14*** | .23*** | .20*** |  |  |
| K | log Liquid wealth | .12** | .12** | .05 | .10* | -.04 | .03 | .08* | .18*** | .20*** | .15*** |  |
| L | log Investments | .08* | .08* | .04 | .06 | -.01 | .04 | .08 | .16*** | .20*** | .16*** | .34*** |
| *Note*. **p* < .05. ***p* < .01. ****p* < .001. *N* = 632. | | | | | | | | | | | | |

| Table S23  *Preliminary Regression Models Predicting Positive Affect (Sample 2, Time 2 Controlling for Time 1)* | | | | | | | | | | | | | | |
| --- | --- | --- | --- | --- | --- | --- | --- | --- | --- | --- | --- | --- | --- | --- |
|  |  | Time 1 well-being only | | |  |  | Demographics only | | |  |  | Financial variables | | |
| Predictor |  | *B* (*SE*) |  | β |  |  | *B* (*SE*) |  | β |  |  | *B* (*SE*) |  | β |
| Intercept |  | 4.07 (0.03) |  | *** |  |  | 4.24 (0.18) |  | *** |  |  | 3.34 (0.48) |  | *** |
| Time 1 Positive affect (centered) |  | 0.73 (0.03) |  | .75*** |  |  | 0.71 (0.03) |  | .73*** |  |  | 0.70 (0.03) |  | .72*** |
| Age |  |  |  |  |  | - | 0.00 (0.00) | - | .04 |  | - | 0.01 (0.00) | - | .05 |
| Female |  |  |  |  |  | - | 0.02 (0.07) | - | .01 |  |  | 0.00 (0.07) |  | .00 |
| Married |  |  |  |  |  |  | 0.19 (0.08) |  | .07* |  |  | 0.18 (0.08) |  | .07* |
| Separated |  |  |  |  |  |  | 0.19 (0.16) |  | .04 |  |  | 0.20 (0.16) |  | .04 |
| Widowed |  |  |  |  |  | - | 0.02 (0.40) | - | .00 |  | - | 0.01 (0.40) | - | .00 |
| Student |  |  |  |  |  | - | 0.03 (0.10) | - | .01 |  | - | 0.03 (0.10) | - | .01 |
| Employed |  |  |  |  |  | - | 0.11 (0.13) | - | .04 |  | - | 0.16 (0.14) | - | .06 |
| Retired |  |  |  |  |  | - | 0.00 (0.17) | - | .00 |  | - | 0.04 (0.17) | - | .01 |
| log Liquid wealth |  |  |  |  |  |  |  |  |  |  |  | 0.22 (0.11) |  | .06* |
| log Income |  |  |  |  |  |  |  |  |  |  |  | 0.02 (0.04) |  | .02 |
| log Investments |  |  |  |  |  |  |  |  |  |  |  | 0.01 (0.02) |  | .01 |
| Debt status (1 = has debt) |  |  |  |  |  |  |  |  |  |  |  | 0.01 (0.07) |  | .00 |
| *R*^2^ (adjusted *R*^2^) |  | .55 (.55) | | |  |  | .56 (.55) | | |  |  | .57 (.56) | | |
| Δ*R*^2^ |  |  |  |  |  |  | .009 |  |  |  |  | .005 |  |  |
| Model *F* (*df*_numerator_, *df*_denominator_) |  | 786.15 (1, 630) | | |  |  | 66.68 (12, 619) | | |  |  | 42.56 (19, 612) | | |
| *F*-change (*df*_numerator_, *df*_denominator_) |  |  | | |  |  | 1.12 (11, 619) | | |  |  | 1.09 (7, 612) | | |
| *Note*. †*p* < .10. **p* < .05. ***p* < .01. ****p* < .001.  Nominal demographic variables (e.g., employment) are dummy coded. Demographic variables were measured at Time 1; all other variables were measured at Time 2 except where noted. Missingness dummy variables are not reported. | | | | | | | | | | | | | | |

| Table S24  *Regression Models for Spending and Spending Variety Predicting Positive Affect (Sample 2, Time 2 Controlling for Time 1)* | | | | | | | | | | | | | | | |
| --- | --- | --- | --- | --- | --- | --- | --- | --- | --- | --- | --- | --- | --- | --- | --- |
|  |  | Model 1: Utilitarian spending and variety | | |  |  | Model 2: Hedonic spending | | |  |  | Model 3: Hedonic variety | | | |
| Predictor |  | *B* (*SE*) |  | β |  |  | *B* (*SE*) |  | β |  |  | *B* (*SE*) |  | β | *r*_semipartial_ |
| Intercept |  | 3.15 (0.49) |  | *** |  |  | 3.13 (0.49) |  | *** |  |  | 3.09 (0.49) |  | *** |  |
| Time 1 Positive affect (centered) |  | 0.70 (0.03) |  | .72*** |  |  | 0.70 (0.03) |  | .72*** |  |  | 0.70 (0.03) |  | .72*** | .68 |
| Age | - | 0.01 (0.00) | - | .06 |  | - | 0.01 (0.00) | - | .06 |  | - | 0.00 (0.00) | - | .05 | .03 |
| Female |  | 0.00 (0.07) |  | .00 |  |  | 0.01 (0.07) |  | .00 |  |  | 0.01 (0.07) |  | .01 | .01 |
| Married |  | 0.17 (0.08) |  | .07* |  |  | 0.17 (0.08) |  | .07* |  |  | 0.18 (0.08) |  | .07* | .06 |
| Separated |  | 0.21 (0.16) |  | .04 |  |  | 0.21 (0.16) |  | .04 |  |  | 0.22 (0.16) |  | .04 | .04 |
| Widowed | - | 0.01 (0.40) | - | .00 |  | - | 0.01 (0.40) | - | .00 |  | - | 0.04 (0.40) | - | .00 | .00 |
| Student | - | 0.07 (0.10) | - | .02 |  | - | 0.07 (0.10) | - | .02 |  | - | 0.07 (0.10) | - | .02 | .02 |
| Employed | - | 0.17 (0.14) | - | .06 |  | - | 0.17 (0.14) | - | .06 |  | - | 0.18 (0.14) | - | .06 | .03 |
| Retired | - | 0.03 (0.17) | - | .01 |  | - | 0.03 (0.17) | - | .01 |  | - | 0.01 (0.17) | - | .00 | .00 |
| log Liquid wealth |  | 0.19 (0.11) |  | .05† |  |  | 0.19 (0.11) |  | .05† |  |  | 0.20 (0.11) |  | .05† | .05 |
| log Income |  | 0.01 (0.04) |  | .01 |  |  | 0.01 (0.04) |  | .01 |  |  | 0.02 (0.04) |  | .02 | .01 |
| log Investments |  | 0.00 (0.02) |  | .01 |  |  | 0.00 (0.02) |  | .01 |  |  | 0.01 (0.02) |  | .01 | .01 |
| Debt status (1 = has debt) |  | 0.00 (0.08) |  | .00 |  |  | 0.00 (0.08) |  | .00 |  | - | 0.00 (0.08) | - | .00 | .00 |
| log Utilitarian spending (exact) |  | 0.13 (0.07) |  | .06* |  |  | 0.12 (0.07) |  | .05† |  |  | 0.10 (0.07) |  | .04 | .04 |
| Utilitarian variety | - | 0.05 (0.06) | - | .02 |  | - | 0.07 (0.07) | - | .03 |  | - | 0.12 (0.07) | - | .06† | .04 |
| log Hedonic spending (exact) |  |  |  |  |  |  | 0.04 (0.06) |  | .02 |  | - | 0.02 (0.07) | - | .01 | .01 |
| Hedonic variety |  |  |  |  |  |  |  |  |  |  |  | 0.16 (0.07) |  | .08* | .06 |
| *R*^2^ (adjusted *R*^2^) |  | .57 (.56) | | |  |  | .57 (.56) | | |  |  | .58 (.56) | | |  |
| Δ*R*^2^ |  | .003 |  |  |  |  | .000 |  |  |  |  | .003 |  |  |  |
| Model *F* (*df*_numerator_, *df*_denominator_) |  | 38.84 (21, 610) | | |  |  | 37.06 (22, 609) | | |  |  | 35.91 (23, 608) | | |  |
| *F*-change (*df*_numerator_, *df*_denominator_) |  | 2.10 (2, 610) | | |  |  | 0.44 (1, 609) | | |  |  | 5.07 (1, 608) | | |  |
| *Note*. †*p* < .10. **p* < .05. ***p* < .01. ****p* < .001.  Nominal demographic variables (e.g., employment) are dummy coded. Demographic variables were measured at Time 1; all other variables were measured at Time 2 except where noted. Missingness dummy variables are not reported. | | | | | | | | | | | | | | | |

| Table S25  *Preliminary Regression Models Predicting Life Satisfaction (Sample 2, Time 2 Controlling for Time 1)* | | | | | | | | | | | | | | |
| --- | --- | --- | --- | --- | --- | --- | --- | --- | --- | --- | --- | --- | --- | --- |
|  |  | Time 1 well-being only | | |  |  | Demographics only | | |  |  | Financial variables | | |
| Predictor |  | *B* (*SE*) |  | β |  |  | *B* (*SE*) |  | β |  |  | *B* (*SE*) |  | β |
| Intercept |  | 4.16 (0.03) |  | *** |  |  | 4.11 (0.15) |  | *** |  |  | 3.86 (0.40) |  | *** |
| Time 1 Life satisfaction (centered) |  | 0.86 (0.02) |  | .84*** |  |  | 0.84 (0.02) |  | .83*** |  |  | 0.84 (0.02) |  | .82*** |
| Age |  |  |  |  |  | - | 0.01 (0.00) | - | .05† |  | - | 0.01 (0.00) | - | .06† |
| Female |  |  |  |  |  |  | 0.03 (0.06) |  | .01 |  |  | 0.04 (0.06) |  | .02 |
| Married |  |  |  |  |  |  | 0.21 (0.07) |  | .08** |  |  | 0.21 (0.07) |  | .08** |
| Separated |  |  |  |  |  |  | 0.28 (0.13) |  | .05* |  |  | 0.28 (0.13) |  | .05* |
| Widowed |  |  |  |  |  |  | 0.18 (0.33) |  | .01 |  |  | 0.17 (0.33) |  | .01 |
| Student |  |  |  |  |  |  | 0.08 (0.08) |  | .03 |  |  | 0.08 (0.08) |  | .02 |
| Employed |  |  |  |  |  |  | 0.11 (0.11) |  | .04 |  |  | 0.12 (0.12) |  | .04 |
| Retired |  |  |  |  |  |  | 0.08 (0.14) |  | .02 |  |  | 0.07 (0.14) |  | .02 |
| log Liquid wealth |  |  |  |  |  |  |  |  |  |  |  | 0.07 (0.09) |  | .02 |
| log Income |  |  |  |  |  |  |  |  |  |  | - | 0.00 (0.03) | - | .00 |
| log Investments |  |  |  |  |  |  |  |  |  |  |  | 0.01 (0.02) |  | .02 |
| Debt status (1 = has debt) |  |  |  |  |  |  |  |  |  |  | - | 0.05 (0.06) | - | .02 |
| *R*^2^ (adjusted *R*^2^) |  | .71 (.71) | | |  |  | .71 (.71) | | |  |  | .71 (.71) | | |
| Δ*R*^2^ |  |  |  |  |  |  | .007 |  |  |  |  | .002 |  |  |
| Model *F* (*df*_numerator_, *df*_denominator_) |  | 1553.90 (1, 630) | | |  |  | 131.96 (12, 619) | | |  |  | 83.19 (19, 612) | | |
| *F*-change (*df*_numerator_, *df*_denominator_) |  |  | | |  |  | 1.49 (11, 619) | | |  |  | 0.60 (7, 612) | | |
| *Note*. †*p* < .10. **p* < .05. ***p* < .01. ****p* < .001.  Nominal demographic variables (e.g., employment) are dummy coded. Missingness dummy variables are not reported. | | | | | | | | | | | | | | |

| Table S26  *Regression Models for Spending and Spending Variety Predicting Life Satisfaction (Sample 2, Time 2 Controlling for Time 1)* | | | | | | | | | | | | | | | |
| --- | --- | --- | --- | --- | --- | --- | --- | --- | --- | --- | --- | --- | --- | --- | --- |
|  |  | Model 1: Utilitarian spending and variety | | |  |  | Model 2: Hedonic spending | | |  |  | Model 3: Hedonic variety | | | |
| Predictor |  | *B* (*SE*) |  | β |  |  | *B* (*SE*) |  | β |  |  | *B* (*SE*) |  | β | *r*_semipartial_ |
| Intercept |  | 3.86 (0.41) |  | *** |  |  | 3.86 (0.41) |  | *** |  |  | 3.82 (0.41) |  | *** |  |
| Time 1 Life satisfaction (centered) |  | 0.84 (0.02) |  | .82*** |  |  | 0.84 (0.02) |  | .82*** |  |  | 0.83 (0.02) |  | .82*** | .76 |
| Age | - | 0.01 (0.00) | - | .05† |  | - | 0.01 (0.00) | - | .05† |  | - | 0.01 (0.00) | - | .05 | .03 |
| Female |  | 0.05 (0.06) |  | .02 |  |  | 0.05 (0.06) |  | .02 |  |  | 0.06 (0.06) |  | .02 | .02 |
| Married |  | 0.21 (0.07) |  | .08** |  |  | 0.21 (0.07) |  | .08** |  |  | 0.22 (0.07) |  | .08** | .06 |
| Separated |  | 0.28 (0.13) |  | .05* |  |  | 0.28 (0.13) |  | .05* |  |  | 0.29 (0.13) |  | .05* | .05 |
| Widowed |  | 0.17 (0.33) |  | .01 |  |  | 0.17 (0.33) |  | .01 |  |  | 0.15 (0.33) |  | .01 | .01 |
| Student |  | 0.07 (0.08) |  | .02 |  |  | 0.07 (0.08) |  | .02 |  |  | 0.06 (0.08) |  | .02 | .02 |
| Employed |  | 0.11 (0.12) |  | .04 |  |  | 0.11 (0.12) |  | .04 |  |  | 0.11 (0.12) |  | .04 | .02 |
| Retired |  | 0.08 (0.14) |  | .02 |  |  | 0.08 (0.14) |  | .02 |  |  | 0.09 (0.14) |  | .02 | .01 |
| log Liquid wealth |  | 0.07 (0.10) |  | .02 |  |  | 0.06 (0.10) |  | .02 |  |  | 0.08 (0.09) |  | .02 | .02 |
| log Income | - | 0.00 (0.03) | - | .00 |  | - | 0.00 (0.03) | - | .00 |  |  | 0.00 (0.03) |  | .00 | .00 |
| log Investments |  | 0.01 (0.02) |  | .02 |  |  | 0.01 (0.02) |  | .02 |  |  | 0.01 (0.02) |  | .02 | .02 |
| Debt status (1 = has debt) | - | 0.04 (0.06) | - | .02 |  | - | 0.04 (0.06) | - | .02 |  | - | 0.04 (0.06) | - | .02 | .01 |
| log Utilitarian spending (exact) |  | 0.02 (0.06) |  | .01 |  |  | 0.01 (0.06) |  | .01 |  | - | 0.00 (0.06) | - | .00 | .00 |
| Utilitarian variety | - | 0.03 (0.05) | - | .01 |  | - | 0.03 (0.06) | - | .02 |  | - | 0.08 (0.06) | - | .04 | .03 |
| log Hedonic spending (exact) |  |  |  |  |  |  | 0.01 (0.05) |  | .00 |  | - | 0.05 (0.06) | - | .02 | .02 |
| Hedonic variety |  |  |  |  |  |  |  |  |  |  |  | 0.14 (0.06) |  | .07* | .05 |
| *R*^2^ (adjusted *R*^2^) |  | .71 (.71) | | |  |  | .72 (.71) | | |  |  | .72 (.71) | | |  |
| Δ*R*^2^ |  | .000 |  |  |  |  | .000 |  |  |  |  | .003 |  |  |  |
| Model *F* (*df*_numerator_, *df*_denominator_) |  | 75.09 (21, 610) | | |  |  | 71.56 (22, 609) | | |  |  | 69.26 (23, 608) | | |  |
| *F*-change (*df*_numerator_, *df*_denominator_) |  | 0.19 (2, 610) | | |  |  | 0.85 (1, 609) | | |  |  | 5.89 (1, 608) | | |  |
| *Note*. †*p* < .10. **p* < .05. ***p* < .01. ****p* < .001.  Nominal demographic variables (e.g., employment) are dummy coded. Missingness dummy variables are not reported. | | | | | | | | | | | | | | | |

| Table S27  *Descriptive Statistics from Sample 3, Time 2* | | | | | | |
| --- | --- | --- | --- | --- | --- | --- |
|  | Mean | Standard deviation | Minimum | Median | Maximum | |
| Age in years | 34.12 | 12.70 | 18 | 31 | 77 | |
| Life satisfaction | 4.04 | 1.56 | 1.00 | 4.20 | 7.00 | |
| Positive affect | 4.11 | 1.41 | 1.00 | 4.00 | 7.00 | |
| Hedonic variety (externally-rated hedonic) | 1.09 | 0.66 | 0.00 | 1.17 | 2.74 | |
| Hedonic variety (self-reported hedonic, ver. 1) | 1.17 | 0.70 | 0.00 | 1.20 | 3.86 | |
| Hedonic variety (self-reported hedonic, ver. 2) | 1.57 | 0.69 | 0.00 | 1.66 | 3.90 | |
| Utilitarian variety (externally-rated utilitarian) | 1.49 | 0.65 | 0.00 | 1.55 | 4.05 | |
| Utilitarian variety (self-reported utilitarian, ver. 1) | 1.32 | 0.68 | 0.00 | 1.36 | 3.27 | |
| Utilitarian variety (self-reported utilitarian, ver. 2) | 1.60 | 0.68 | 0.00 | 1.67 | 3.35 | |
| Hedonic spending ($; externally-rated hedonic) | 1359.26 | 2580.34 | 0.00 | 718.00 | 41910.00 | |
| Hedonic spending ($; self-reported hedonic, ver. 1) | 1825.85 | 3700.45 | 0.00 | 724.50 | 40460.00 | |
| Hedonic spending ($; self-reported hedonic, ver. 2) | 3787.05 | 15725.47 | 0.00 | 1495.75 | 363590.00 | |
| Utilitarian spending ($; externally-rated utilitarian) | 26439.28 | 371372.67 | 0.00 | 6349.50 | 9927353.00 | |
| Utilitarian spending ($; self-reported utilitarian, ver. 1) | 23277.20 | 370279.37 | 0.00 | 5049.20 | 9928423.00 | |
| Utilitarian spending ($; self-reported utilitarian, ver. 2) | 29817.94 | 118371.43 | 0.00 | 14808.25 | 2930121.50 | |
| Income (annual, $) | 38989.53 | 84534.54 | 0.00 | 25000.00 | 2000000.00 | |
| Liquid wealth (monthly, $) | 27513.21 | 411619.38 | -700.00 | 1950.00 | 10850000.00 | |
| Investments (time-of-study, $) | 41655.51 | 150830.25 | 0.00 | 0.00 | 1500000.00 | |
| *Categorical Variables* | Category | *n* |  |  |  | |
| Gender (0 missing) | Female | 364 |  |  |  | |
|  | Male | 356 |  |  |  | |
| Relationship status (6 missing) | Married | 215 |  |  |  | |
|  | In relationship | 157 |  |  |  | |
|  | Separated | 43 |  |  |  | |
|  | Widowed | 9 |  |  |  | |
|  | Single | 290 |  |  |  | |
| Employment status (0 missing) | Employed | 493 |  |  |  | |
|  | Student | 221 | (140 student and employed) | | |  |
|  | Retired or not seeking | 82 | (19 not seeking and student) | | |  |
|  | None | 83 |  |  |  | |
| Debt status (0 missing) | Has debt | 347 |  |  |  | |
|  | No debt | 373 |  |  |  | |
| *Note*. Life satisfaction was measured using the Satisfaction With Life Scale (Diener et al., 1985). Positive affect was measured with the Affect-Adjective Scale (Diener & Emmons, 1984). Both outcomes used a 7-point Likert scale.  For self-reported hedonic vs. utilitarian variables, version 1 excludes categories rated as equally hedonic and utilitarian; version 2 allocates equally hedonic/utilitarian categories as 50% hedonic and utilitarian.  All values are Time 2 statistics. Total *N* = 720. Employment status sample sizes sum to more than the total *N* because some participants reported multiple employment statuses (e.g., employed and student). | | | | | | |

| Table S28  *Correlation Matrix of Sample 3, Time 2 Variables* | | | | | | | | | | | |
| --- | --- | --- | --- | --- | --- | --- | --- | --- | --- | --- | --- |
|  |  | A | B | C | D | E | F | G | H | I |  |
| A | Life satisfaction (Time 1) |  |  |  |  |  |  |  |  |  |  |
| B | Life satisfaction (Time 2) | .87*** |  |  |  |  |  |  |  |  |  |
| C | Positive affect (Time 1) | .72*** | .66*** |  |  |  |  |  |  |  |  |
| D | Positive affect (Time 2) | .65*** | .71*** | .77*** |  |  |  |  |  |  |  |
| E | Hedonic variety (externally-rated hedonic) | .13*** | .17*** | .11** | .12** |  |  |  |  |  |  |
| F | Hedonic variety (self-reported hedonic, ver. 1) | .16*** | .19*** | .15*** | .16*** | .78*** |  |  |  |  |  |
| G | Hedonic variety (self-reported hedonic, ver. 2) | .15*** | .18*** | .14*** | .13*** | .79*** | .82*** |  |  |  |  |
| H | Utilitarian variety (externally-rated utilitarian) | .17*** | .16*** | .13*** | .08* | .43*** | .44*** | .57*** |  |  |  |
| I | Utilitarian variety (self-reported utilitarian, ver. 1) | .13*** | .11** | .10* | .05 | .36*** | .30*** | .34*** | .85*** |  |  |
| J | Utilitarian variety (self-reported utilitarian, ver. 2) | .13*** | .12** | .09* | .04 | .43*** | .33*** | .51*** | .92*** | .89*** |  |
| K | log Total hedonic spending (externally-rated hedonic) | .15*** | .18*** | .11** | .14*** | .60*** | .52*** | .62*** | .43*** | .37*** |  |
| L | log Total hedonic spending (self-reported hedonic, ver. 1) | .16*** | .20*** | .11** | .16*** | .54*** | .63*** | .59*** | .42*** | .32*** |  |
| M | log Total hedonic spending (self-reported hedonic, ver. 2) | .18*** | .24*** | .13*** | .16*** | .51*** | .52*** | .59*** | .49*** | .34*** |  |
| N | log Total utilitarian spending (externally-rated utilitarian) | .13*** | .18*** | .08* | .10** | .35*** | .31*** | .37*** | .51*** | .49*** |  |
| O | log Total utilitarian spending (self-reported utilitarian, ver. 1) | .10** | .13*** | .06 | .07 | .32*** | .25*** | .31*** | .50*** | .55*** |  |
| P | log Total utilitarian spending (self-reported utilitarian, ver. 2) | .12** | .16*** | .08* | .09* | .35*** | .26*** | .34*** | .50*** | .53*** |  |
| Q | Age | .06 | .04 | -.01 | .02 | .05 | .03 | .09* | .32*** | .35*** |  |
| R | log Income | .12** | .14*** | .07 | .05 | .19*** | .21*** | .23*** | .34*** | .30*** |  |
| S | log Liquid wealth | .23*** | .26*** | .15*** | .15*** | .16*** | .19*** | .21*** | .20*** | .16*** |  |
| T | log Investments | .22*** | .24*** | .15*** | .16*** | .16*** | .16*** | .15*** | .20*** | .23*** |  |
| U | Debt status (1 = has debt) | -.02 | -.04 | -.03 | -.05 | .14*** | .14*** | .15*** | .21*** | .20*** |  |

| Table S28 (continued) | | | | | | | | | | | | |
| --- | --- | --- | --- | --- | --- | --- | --- | --- | --- | --- | --- | --- |
|  |  | J | K | L | M | N | O | P | Q | R | S | T |
| J | Utilitarian variety (self-reported utilitarian, ver. 2) |  |  |  |  |  |  |  |  |  |  |  |
| K | log Total hedonic spending (externally-rated hedonic) | .44*** |  |  |  |  |  |  |  |  |  |  |
| L | log Total hedonic spending (self-reported hedonic, ver. 1) | .33*** | .77*** |  |  |  |  |  |  |  |  |  |
| M | log Total hedonic spending (self-reported hedonic, ver. 2) | .45*** | .77*** | .82*** |  |  |  |  |  |  |  |  |
| N | log Total utilitarian spending (externally-rated utilitarian) | .47*** | .52*** | .50*** | .63*** |  |  |  |  |  |  |  |
| O | log Total utilitarian spending (self-reported utilitarian, ver. 1) | .47*** | .44*** | .43*** | .46*** | .90*** |  |  |  |  |  |  |
| P | log Total utilitarian spending (self-reported utilitarian, ver. 2) | .49*** | .50*** | .45*** | .58*** | .96*** | .94*** |  |  |  |  |  |
| Q | Age | .33*** | .15*** | .07 | .14*** | .25*** | .26*** | .26*** |  |  |  |  |
| R | log Income | .30*** | .25*** | .27*** | .33*** | .42*** | .36*** | .38*** | .22*** |  |  |  |
| S | log Liquid wealth | .15*** | .19*** | .27*** | .30*** | .30*** | .27*** | .27*** | .01 | .33*** |  |  |
| T | log Investments | .18*** | .21*** | .24*** | .26*** | .35*** | .35*** | .36*** | .19*** | .33*** | .49*** |  |
| U | Debt status (1 = has debt) | .20*** | .16*** | .11** | .15*** | .19*** | .18*** | .18*** | .13*** | .11** | -.16*** | .03 |
| *Note*. **p* < .05. ***p* < .01. ****p* < .001. *N* = 718.  For self-reported hedonic vs. utilitarian variables, version 1 excludes categories rated as equally hedonic and utilitarian; version 2 allocates equally hedonic/utilitarian categories as 50% hedonic and utilitarian. | | | | | | | | | | | | |

| Table S29  *Preliminary Regression Models Predicting Positive Affect (Sample 3, Time 2 Controlling for Time 1)* | | | | | | | | | | | | | | |
| --- | --- | --- | --- | --- | --- | --- | --- | --- | --- | --- | --- | --- | --- | --- |
|  |  | Time 1 well-being only | | |  |  | Demographics only | | |  |  | Financial variables | | |
| Predictor |  | *B* (*SE*) |  | β |  |  | *B* (*SE*) |  | β |  |  | *B* (*SE*) |  | β |
| Intercept |  | 4.10 (0.03) |  | .00*** |  |  | 3.86 (0.14) |  | .00*** |  |  | 3.89 (0.27) |  | .00*** |
| Time 1 Positive affect (centered) |  | 0.76 (0.02) |  | .77*** |  |  | 0.73 (0.02) |  | .74*** |  |  | 0.72 (0.02) |  | .74*** |
| Age |  |  |  |  |  |  | 0.00 (0.00) |  | .01 |  |  | 0.00 (0.00) |  | .02 |
| Female |  |  |  |  |  | - | 0.08 (0.07) | - | .03 |  | - | 0.06 (0.07) | - | .02 |
| Married |  |  |  |  |  |  | 0.24 (0.09) |  | .08** |  |  | 0.26 (0.10) |  | .09** |
| In relationship, unmarried |  |  |  |  |  |  | 0.09 (0.09) |  | .03 |  |  | 0.09 (0.09) |  | .03 |
| Separated |  |  |  |  |  | - | 0.03 (0.16) | - | .00 |  | - | 0.02 (0.16) | - | .00 |
| Widowed |  |  |  |  |  |  | 0.27 (0.32) |  | .02 |  |  | 0.23 (0.33) |  | .02 |
| Student |  |  |  |  |  |  | 0.11 (0.08) |  | .04 |  |  | 0.13 (0.08) |  | .04 |
| Employed |  |  |  |  |  |  | 0.12 (0.09) |  | .04 |  |  | 0.13 (0.09) |  | .04 |
| Retired or not seeking employment |  |  |  |  |  |  | 0.20 (0.13) |  | .05 |  |  | 0.18 (0.13) |  | .04 |
| log Liquid wealth |  |  |  |  |  |  |  |  |  |  |  | 0.02 (0.07) |  | .01 |
| log Income |  |  |  |  |  |  |  |  |  |  | - | 0.03 (0.03) | - | .03 |
| log Investments |  |  |  |  |  |  |  |  |  |  |  | 0.02 (0.02) |  | .02 |
| Debt status (1 = has debt) |  |  |  |  |  |  |  |  |  |  | - | 0.11 (0.07) | - | .04 |
| *R*^2^ (adjusted *R*^2^) |  | .60 (.60) | | |  |  | .61 (.60) | | |  |  | .61 (.60) | | |
| Δ*R*^2^ |  |  | | |  |  | .014 | | |  |  | .004 | | |
| Model *F* (*df*_numerator_, *df*_denominator_) |  | 1055.87 (1, 716) | | |  |  | 100.14 (11, 706) | | |  |  | 61.58 (18, 699) | | |
| *F*-change (*df*_numerator_, *df*_denominator_) |  |  | | |  |  | 2.44 (10, 706) | | |  |  | 0.99 (7, 699) | | |
| *Note*. **p* < .05. ***p* < .01. ****p* < .001.  Nominal demographic variables (e.g., employment) are dummy coded. Missingness dummy variables are not reported. | | | | | | | | | | | | | | |

| Table S30  *Regression Models for Spending and Spending Variety Predicting Positive Affect (Sample 3, Time 2 Controlling for Time 1; Externally-Rated Hedonic/Utilitarian Categories)* | | | | | | | | | | | | | | | |
| --- | --- | --- | --- | --- | --- | --- | --- | --- | --- | --- | --- | --- | --- | --- | --- |
|  |  | Model 1: Utilitarian spending and variety | | |  |  | Model 2: Hedonic spending | | |  |  | Model 3: Hedonic variety | | | |
| Predictor |  | *B* (*SE*) |  | β |  |  | *B* (*SE*) |  | β |  |  | *B* (*SE*) |  | β | *r*_semipartial_ |
| Intercept |  | 3.76 (0.28) |  | .00*** |  |  | 3.70 (0.28) |  | .00*** |  |  | 3.71 (0.28) |  | .00*** |  |
| Time 1 Positive affect (centered) |  | 0.73 (0.02) |  | .74*** |  |  | 0.73 (0.02) |  | .74*** |  |  | 0.73 (0.02) |  | .74*** | .68 |
| Age |  | 0.00 (0.00) |  | .03 |  |  | 0.00 (0.00) |  | .03 |  |  | 0.00 (0.00) |  | .03 | .02 |
| Female | - | 0.07 (0.07) | - | .03 |  | - | 0.07 (0.07) | - | .03 |  | - | 0.08 (0.07) | - | .03 | .03 |
| Married |  | 0.28 (0.10) |  | .09** |  |  | 0.27 (0.10) |  | .09** |  |  | 0.27 (0.10) |  | .09** | .07 |
| In relationship, unmarried |  | 0.10 (0.09) |  | .03 |  |  | 0.08 (0.09) |  | .02 |  |  | 0.08 (0.09) |  | .02 | .02 |
| Separated |  | 0.00 (0.16) |  | .00 |  |  | 0.00 (0.16) |  | .00 |  |  | 0.00 (0.16) |  | .00 | .00 |
| Widowed |  | 0.23 (0.32) |  | .02 |  |  | 0.25 (0.32) |  | .02 |  |  | 0.24 (0.32) |  | .02 | .02 |
| Student |  | 0.12 (0.08) |  | .04 |  |  | 0.12 (0.08) |  | .04 |  |  | 0.12 (0.08) |  | .04 | .03 |
| Employed |  | 0.12 (0.09) |  | .04 |  |  | 0.11 (0.09) |  | .04 |  |  | 0.12 (0.09) |  | .04 | .03 |
| Retired or not seeking employment |  | 0.17 (0.13) |  | .04 |  |  | 0.17 (0.13) |  | .04 |  |  | 0.17 (0.13) |  | .04 | .03 |
| log Liquid wealth |  | 0.03 (0.07) |  | .02 |  |  | 0.03 (0.07) |  | .01 |  |  | 0.03 (0.07) |  | .01 | .01 |
| log Income | - | 0.03 (0.04) | - | .03 |  | - | 0.03 (0.04) | - | .03 |  | - | 0.03 (0.04) | - | .03 | .02 |
| log Investments |  | 0.01 (0.02) |  | .02 |  |  | 0.01 (0.02) |  | .01 |  |  | 0.01 (0.02) |  | .01 | .01 |
| Debt status (1 = has debt) | - | 0.09 (0.07) | - | .03 |  | - | 0.10 (0.07) | - | .03 |  | - | 0.10 (0.07) | - | .03 | .03 |
| log Utilitarian spending (exact) |  | 0.08 (0.05) |  | .04 |  |  | 0.04 (0.06) |  | .02 |  |  | 0.04 (0.06) |  | .02 | .02 |
| Utilitarian variety | - | 0.15 (0.06) | - | .07* |  | - | 0.18 (0.06) | - | .08** |  | - | 0.19 (0.07) | - | .09** | .07 |
| log Hedonic spending (exact) |  |  |  |  |  |  | 0.10 (0.05) |  | .06* |  |  | 0.09 (0.06) |  | .05 | .04 |
| Hedonic variety |  |  |  |  |  |  |  |  |  |  |  | 0.04 (0.07) |  | .02 | .01 |
| *R*^2^ (adjusted *R*^2^) |  | .62 (.61) | | |  |  | .62 (.61) | | |  |  | .62 (.61) | | |  |
| Δ*R*^2^ |  | .003 | | |  |  | .002 | | |  |  | .000 | | |  |
| Model *F* (*df*_numerator_, *df*_denominator_) |  | 56.05 (20, 697) | | |  |  | 53.86 (21, 696) | | |  |  | 51.38 (22, 695) | | |  |
| *F*-change (*df*_numerator_, *df*_denominator_) |  | 3.03 (2, 697) | | |  |  | 4.53 (1, 696) | | |  |  | 0.31 (1, 695) | | |  |
| *Note*. **p* < .05. ***p* < .01. ****p* < .001.  Nominal demographic variables (e.g., employment) are dummy coded. Missingness dummy variables are not reported. | | | | | | | | | | | | | | | |

| Table S31  *Regression Models for Spending and Spending Variety Predicting Positive Affect (Sample 3, Time 2 Controlling for Time 1; Self-Reported Hedonic/Utilitarian Categories, Equally Hedonic/Utilitarian Categories Allocated 50% Hedonic/50% Utilitarian)* | | | | | | | | | | | | | | | |
| --- | --- | --- | --- | --- | --- | --- | --- | --- | --- | --- | --- | --- | --- | --- | --- |
|  |  | Model 1: Utilitarian spending and variety | | |  |  | Model 2: Hedonic spending | | |  |  | Model 3: Hedonic variety | | | |
| Predictor |  | *B* (*SE*) |  | β |  |  | *B* (*SE*) |  | β |  |  | *B* (*SE*) |  | β | *r*_semipartial_ |
| Intercept |  | 3.80 (0.28) |  | .00*** |  |  | 3.76 (0.28) |  | .00*** |  |  | 3.77 (0.28) |  | .00*** |  |
| Time 1 Positive affect (centered) |  | 0.73 (0.02) |  | .74*** |  |  | 0.73 (0.02) |  | .74*** |  |  | 0.72 (0.02) |  | .74*** | .68 |
| Age |  | 0.00 (0.00) |  | .03 |  |  | 0.00 (0.00) |  | .04 |  |  | 0.00 (0.00) |  | .04 | .03 |
| Female | - | 0.07 (0.07) | - | .02 |  | - | 0.07 (0.07) | - | .03 |  | - | 0.07 (0.07) | - | .03 | .02 |
| Married |  | 0.28 (0.10) |  | .09** |  |  | 0.26 (0.10) |  | .08** |  |  | 0.26 (0.10) |  | .08** | .06 |
| In relationship, unmarried |  | 0.10 (0.09) |  | .03 |  |  | 0.08 (0.09) |  | .02 |  |  | 0.08 (0.09) |  | .02 | .02 |
| Separated | - | 0.00 (0.16) | - | .00 |  | - | 0.01 (0.16) | - | .00 |  | - | 0.02 (0.16) | - | .00 | .00 |
| Widowed |  | 0.22 (0.32) |  | .02 |  |  | 0.23 (0.32) |  | .02 |  |  | 0.23 (0.32) |  | .02 | .02 |
| Student |  | 0.11 (0.08) |  | .04 |  |  | 0.11 (0.08) |  | .03 |  |  | 0.10 (0.08) |  | .03 | .03 |
| Employed |  | 0.12 (0.09) |  | .04 |  |  | 0.12 (0.09) |  | .04 |  |  | 0.12 (0.09) |  | .04 | .03 |
| Retired or not seeking employment |  | 0.17 (0.13) |  | .04 |  |  | 0.18 (0.13) |  | .04 |  |  | 0.18 (0.13) |  | .04 | .03 |
| log Liquid wealth |  | 0.03 (0.07) |  | .01 |  |  | 0.00 (0.07) |  | .00 |  |  | 0.00 (0.07) |  | .00 | .00 |
| log Income | - | 0.03 (0.03) | - | .02 |  | - | 0.03 (0.03) | - | .03 |  | - | 0.03 (0.03) | - | .03 | .02 |
| log Investments |  | 0.01 (0.02) |  | .02 |  |  | 0.01 (0.02) |  | .02 |  |  | 0.01 (0.02) |  | .02 | .01 |
| Debt status (1 = has debt) | - | 0.09 (0.07) | - | .03 |  | - | 0.10 (0.07) | - | .04 |  | - | 0.10 (0.07) | - | .04 | .03 |
| log Utilitarian spending (exact) |  | 0.07 (0.05) |  | .04 |  |  | 0.01 (0.06) |  | .01 |  |  | 0.02 (0.06) |  | .01 | .01 |
| Utilitarian variety | - | 0.17 (0.06) | - | .08** |  | - | 0.20 (0.06) | - | .10*** |  | - | 0.22 (0.07) | - | .10*** | .08 |
| log Hedonic spending (exact) |  |  |  |  |  |  | 0.14 (0.05) |  | .08** |  |  | 0.12 (0.06) |  | .07* | .05 |
| Hedonic variety |  |  |  |  |  |  |  |  |  |  |  | 0.05 (0.06) |  | .02 | .02 |
| *R*^2^ (adjusted *R*^2^) |  | .62 (.61) | | |  |  | .62 (.61) | | |  |  | .62 (.61) | | |  |
| Δ*R*^2^ |  | .005 | | |  |  | .004 | | |  |  | .000 | | |  |
| Model *F* (*df*_numerator_, *df*_denominator_) |  | 56.33 (20, 697) | | |  |  | 54.43 (21, 696) | | |  |  | 51.95 (22, 695) | | |  |
| *F*-change (*df*_numerator_, *df*_denominator_) |  | 4.12 (2, 697) | | |  |  | 6.91 (1, 696) | | |  |  | 0.53 (1, 695) | | |  |
| *Note*. **p* < .05. ***p* < .01. ****p* < .001.  Nominal demographic variables (e.g., employment) are dummy coded. Missingness dummy variables are not reported. | | | | | | | | | | | | | | | |

| Table S32  *Regression Models for Spending and Spending Variety Predicting Positive Affect (Sample 3, Time 2 Controlling for Time 1; Self-Reported Hedonic/Utilitarian Categories, Excluding Equally Hedonic/Utilitarian Categories)* | | | | | | | | | | | | | | | |
| --- | --- | --- | --- | --- | --- | --- | --- | --- | --- | --- | --- | --- | --- | --- | --- |
|  |  | Model 1: Utilitarian spending and variety | | |  |  | Model 2: Hedonic spending | | |  |  | Model 3: Hedonic variety | | | |
| Predictor |  | *B* (*SE*) |  | β |  |  | *B* (*SE*) |  | β |  |  | *B* (*SE*) |  | β | *r*_semipartial_ |
| Intercept |  | 3.97 (0.28) |  | .00*** |  |  | 3.82 (0.28) |  | .00*** |  |  | 3.83 (0.28) |  | .00*** |  |
| Time 1 Positive affect (centered) |  | 0.72 (0.02) |  | .74*** |  |  | 0.72 (0.03) |  | .73*** |  |  | 0.72 (0.03) |  | .73*** | .67 |
| Age |  | 0.00 (0.00) |  | .03 |  |  | 0.00 (0.00) |  | .03 |  |  | 0.00 (0.00) |  | .04 | .03 |
| Female | - | 0.06 (0.07) | - | .02 |  | - | 0.07 (0.07) | - | .02 |  | - | 0.07 (0.07) | - | .03 | .02 |
| Married |  | 0.29 (0.10) |  | .09** |  |  | 0.28 (0.10) |  | .09** |  |  | 0.28 (0.10) |  | .09** | .07 |
| In relationship, unmarried |  | 0.09 (0.09) |  | .03 |  |  | 0.08 (0.09) |  | .02 |  |  | 0.08 (0.09) |  | .02 | .02 |
| Separated | - | 0.01 (0.16) | - | .00 |  |  | 0.00 (0.16) |  | .00 |  | - | 0.00 (0.16) | - | .00 | .00 |
| Widowed |  | 0.24 (0.32) |  | .02 |  |  | 0.21 (0.32) |  | .02 |  |  | 0.20 (0.32) |  | .02 | .01 |
| Student |  | 0.13 (0.08) |  | .04 |  |  | 0.12 (0.08) |  | .04 |  |  | 0.12 (0.08) |  | .04 | .03 |
| Employed |  | 0.16 (0.09) |  | .05† |  |  | 0.16 (0.09) |  | .05† |  |  | 0.16 (0.09) |  | .05† | .04 |
| Retired or not seeking employment |  | 0.20 (0.13) |  | .04 |  |  | 0.22 (0.13) |  | .05† |  |  | 0.23 (0.13) |  | .05† | .04 |
| log Liquid wealth |  | 0.02 (0.07) |  | .01 |  |  | 0.02 (0.07) |  | .01 |  |  | 0.02 (0.07) |  | .01 | .01 |
| log Income | - | 0.02 (0.04) | - | .02 |  | - | 0.02 (0.03) | - | .02 |  | - | 0.02 (0.03) | - | .02 | .01 |
| log Investments |  | 0.02 (0.02) |  | .03 |  |  | 0.01 (0.02) |  | .02 |  |  | 0.01 (0.02) |  | .02 | .02 |
| Debt status (1 = has debt) | - | 0.09 (0.07) | - | .03 |  | - | 0.09 (0.07) | - | .03 |  | - | 0.09 (0.07) | - | .03 | .03 |
| log Utilitarian spending (exact) | - | 0.02 (0.05) | - | .01 |  | - | 0.05 (0.05) | - | .04 |  | - | 0.05 (0.05) | - | .03 | .03 |
| Utilitarian variety | - | 0.10 (0.06) | - | .05† |  | - | 0.13 (0.06) | - | .06* |  | - | 0.14 (0.06) | - | .07* | .05 |
| log Hedonic spending (exact) |  |  |  |  |  |  | 0.11 (0.05) |  | .07* |  |  | 0.08 (0.05) |  | .05 | .04 |
| Hedonic variety |  |  |  |  |  |  |  |  |  |  |  | 0.06 (0.06) |  | .03 | .02 |
| *R*^2^ (adjusted *R*^2^) |  | .62 (.60) | | |  |  | .62 (.61) | | |  |  | .62 (.61) | | |  |
| Δ*R*^2^ |  | .002 | | |  |  | .003 | | |  |  | .000 | | |  |
| Model *F* (*df*_numerator_, *df*_denominator_) |  | 55.81 (20, 697) | | |  |  | 53.77 (21, 696) | | |  |  | 51.36 (22, 695) | | |  |
| *F*-change (*df*_numerator_, *df*_denominator_) |  | 2.12 (2, 697) | | |  |  | 5.63 (1, 696) | | |  |  | 0.87 (1, 695) | | |  |
| *Note*. †*p* < .10. **p* < .05. ***p* < .01. ****p* < .001.  Nominal demographic variables (e.g., employment) are dummy coded. Missingness dummy variables are not reported. | | | | | | | | | | | | | | | |

| Table S33  *Preliminary Regression Models Predicting Life Satisfaction (Sample 3, Time 2 Controlling for Time 1)* | | | | | | | | | | | | | | |
| --- | --- | --- | --- | --- | --- | --- | --- | --- | --- | --- | --- | --- | --- | --- |
|  |  | Time 1 well-being only | | |  |  | Demographics only | | |  |  | Financial variables | | |
| Predictor |  | *B* (*SE*) |  | β |  |  | *B* (*SE*) |  | β |  |  | *B* (*SE*) |  | β |
| Intercept |  | 4.04 (0.03) |  | .00*** |  |  | 3.80 (0.12) |  | .00*** |  |  | 3.45 (0.23) |  | .00*** |
| Time 1 Life satisfaction (centered) |  | 0.87 (0.02) |  | .87*** |  |  | 0.83 (0.02) |  | .83*** |  |  | 0.82 (0.02) |  | .82*** |
| Age |  |  |  |  |  | - | 0.00 (0.00) | - | .03 |  | - | 0.00 (0.00) | - | .03 |
| Female |  |  |  |  |  | - | 0.02 (0.06) | - | .01 |  |  | 0.01 (0.06) |  | .00 |
| Married |  |  |  |  |  |  | 0.30 (0.08) |  | .09*** |  |  | 0.29 (0.08) |  | .09*** |
| In relationship, unmarried |  |  |  |  |  |  | 0.14 (0.08) |  | .04† |  |  | 0.13 (0.08) |  | .03† |
| Separated |  |  |  |  |  |  | 0.08 (0.13) |  | .01 |  |  | 0.10 (0.13) |  | .02 |
| Widowed |  |  |  |  |  |  | 0.23 (0.27) |  | .02 |  |  | 0.16 (0.27) |  | .01 |
| Student |  |  |  |  |  |  | 0.18 (0.07) |  | .05** |  |  | 0.20 (0.07) |  | .06** |
| Employed |  |  |  |  |  |  | 0.24 (0.07) |  | .07** |  |  | 0.18 (0.08) |  | .05* |
| Retired or not seeking employment |  |  |  |  |  |  | 0.28 (0.11) |  | .06* |  |  | 0.29 (0.11) |  | .06** |
| log Liquid wealth |  |  |  |  |  |  |  |  |  |  |  | 0.08 (0.06) |  | .03 |
| log Income |  |  |  |  |  |  |  |  |  |  |  | 0.03 (0.03) |  | .03 |
| log Investments |  |  |  |  |  |  |  |  |  |  |  | 0.01 (0.02) |  | .02 |
| Debt status (1 = has debt) |  |  |  |  |  |  |  |  |  |  | - | 0.08 (0.06) | - | .03 |
| *R*^2^ (adjusted *R*^2^) |  | .76 (.76) | | |  |  | .77 (.77) | | |  |  | .78 (.77) | | |
| Δ*R*^2^ |  |  | | |  |  | .014 | | |  |  | .004 | | |
| Model *F* (*df*_numerator_, *df*_denominator_) |  | 2271.35 (1, 716) | | |  |  | 219.78 (11, 706) | | |  |  | 136.27 (18, 699) | | |
| *F*-change (*df*_numerator_, *df*_denominator_) |  |  | | |  |  | 4.26 (10, 706) | | |  |  | 1.92 (7, 699) | | |
| *Note*. †*p* < .10. **p* < .05. ***p* < .01. ****p* < .001.  Nominal demographic variables (e.g., employment) are dummy coded. Missingness dummy variables are not reported. | | | | | | | | | | | | | | |

| Table S34  *Regression Models for Spending and Spending Variety Predicting Life Satisfaction (Sample 3, Time 2 Controlling for Time 1; Externally-Rated Hedonic/Utilitarian Categories)* | | | | | | | | | | | | | | | |
| --- | --- | --- | --- | --- | --- | --- | --- | --- | --- | --- | --- | --- | --- | --- | --- |
|  |  | Model 1: Utilitarian spending and variety | | |  |  | Model 2: Hedonic spending | | |  |  | Model 3: Hedonic variety | | | |
| Predictor |  | *B* (*SE*) |  | β |  |  | *B* (*SE*) |  | β |  |  | *B* (*SE*) |  | β | *r*_semipartial_ |
| Intercept |  | 3.29 (0.24) |  | .00*** |  |  | 3.27 (0.24) |  | .00*** |  |  | 3.29 (0.24) |  | .00*** |  |
| Time 1 Life satisfaction (centered) |  | 0.83 (0.02) |  | .83*** |  |  | 0.83 (0.02) |  | .82*** |  |  | 0.83 (0.02) |  | .82*** | .73 |
| Age | - | 0.00 (0.00) | - | .04 |  | - | 0.00 (0.00) | - | .04 |  | - | 0.00 (0.00) | - | .03 | .02 |
| Female |  | 0.00 (0.06) |  | .00 |  |  | 0.00 (0.06) |  | .00 |  | - | 0.00 (0.06) | - | .00 | .00 |
| Married |  | 0.28 (0.08) |  | .08*** |  |  | 0.27 (0.08) |  | .08*** |  |  | 0.28 (0.08) |  | .08*** | .06 |
| In relationship, unmarried |  | 0.12 (0.08) |  | .03 |  |  | 0.12 (0.08) |  | .03 |  |  | 0.12 (0.08) |  | .03 | .03 |
| Separated |  | 0.10 (0.13) |  | .01 |  |  | 0.10 (0.13) |  | .01 |  |  | 0.10 (0.13) |  | .01 | .01 |
| Widowed |  | 0.17 (0.27) |  | .01 |  |  | 0.18 (0.27) |  | .01 |  |  | 0.16 (0.27) |  | .01 | .01 |
| Student |  | 0.19 (0.07) |  | .06** |  |  | 0.19 (0.07) |  | .06** |  |  | 0.19 (0.07) |  | .06** | .05 |
| Employed |  | 0.17 (0.08) |  | .05* |  |  | 0.17 (0.08) |  | .05* |  |  | 0.17 (0.08) |  | .05* | .04 |
| Retired or not seeking employment |  | 0.27 (0.11) |  | .05* |  |  | 0.27 (0.11) |  | .05* |  |  | 0.29 (0.11) |  | .06** | .05 |
| log Liquid wealth |  | 0.07 (0.06) |  | .03 |  |  | 0.07 (0.06) |  | .03 |  |  | 0.06 (0.06) |  | .03 | .02 |
| log Income |  | 0.02 (0.03) |  | .02 |  |  | 0.02 (0.03) |  | .02 |  |  | 0.02 (0.03) |  | .02 | .01 |
| log Investments |  | 0.01 (0.02) |  | .01 |  |  | 0.01 (0.02) |  | .01 |  |  | 0.01 (0.02) |  | .01 | .01 |
| Debt status (1 = has debt) | - | 0.09 (0.06) | - | .03 |  | - | 0.09 (0.06) | - | .03 |  | - | 0.09 (0.06) | - | .03 | .03 |
| log Utilitarian spending (exact) |  | 0.10 (0.05) |  | .05* |  |  | 0.09 (0.05) |  | .05† |  |  | 0.10 (0.05) |  | .05† | .03 |
| Utilitarian variety | - | 0.06 (0.05) | - | .03 |  | - | 0.07 (0.05) | - | .03 |  | - | 0.10 (0.06) | - | .04† | .03 |
| log Hedonic spending (exact) |  |  |  |  |  |  | 0.03 (0.04) |  | .02 |  | - | 0.01 (0.05) | - | .01 | .00 |
| Hedonic variety |  |  |  |  |  |  |  |  |  |  |  | 0.09 (0.06) |  | .04† | .03 |
| *R*^2^ (adjusted *R*^2^) |  | .78 (.77) | | |  |  | .78 (.77) | | |  |  | .78 (.77) | | |  |
| Δ*R*^2^ |  | .002 | | |  |  | .000 | | |  |  | .001 | | |  |
| Model *F* (*df*_numerator_, *df*_denominator_) |  | 123.49 (20, 697) | | |  |  | 117.56 (21, 696) | | |  |  | 112.65 (22, 695) | | |  |
| *F*-change (*df*_numerator_, *df*_denominator_) |  | 2.66 (2, 697) | | |  |  | 0.53 (1, 696) | | |  |  | 2.89 (1, 695) | | |  |
| *Note*. †*p* < .10. **p* < .05. ***p* < .01. ****p* < .001.  Nominal demographic variables (e.g., employment) are dummy coded. Missingness dummy variables are not reported. | | | | | | | | | | | | | | | |

| Table S35  *Regression Models for Spending and Spending Variety Predicting Life Satisfaction (Sample 3, Time 2 Controlling for Time 1; Self-Reported Hedonic/Utilitarian Categories, Equally Hedonic/Utilitarian Categories Allocated 50% Hedonic/50% Utilitarian)* | | | | | | | | | | | | | | | |
| --- | --- | --- | --- | --- | --- | --- | --- | --- | --- | --- | --- | --- | --- | --- | --- |
|  |  | Model 1: Utilitarian spending and variety | | |  |  | Model 2: Hedonic spending | | |  |  | Model 3: Hedonic variety | | | |
| Predictor |  | *B* (*SE*) |  | β |  |  | *B* (*SE*) |  | β |  |  | *B* (*SE*) |  | β | *r*_semipartial_ |
| Intercept |  | 3.32 (0.24) |  | .00*** |  |  | 3.27 (0.24) |  | .00*** |  |  | 3.27 (0.24) |  | .00*** |  |
| Time 1 Life satisfaction (centered) |  | 0.83 (0.02) |  | .83*** |  |  | 0.82 (0.02) |  | .82*** |  |  | 0.82 (0.02) |  | .82*** | .73 |
| Age | - | 0.00 (0.00) | - | .03 |  | - | 0.00 (0.00) | - | .03 |  | - | 0.00 (0.00) | - | .03 | .02 |
| Female |  | 0.00 (0.06) |  | .00 |  |  | 0.00 (0.06) |  | .00 |  |  | 0.00 (0.06) |  | .00 | .00 |
| Married |  | 0.28 (0.08) |  | .08*** |  |  | 0.25 (0.08) |  | .07** |  |  | 0.25 (0.08) |  | .07** | .06 |
| In relationship, unmarried |  | 0.13 (0.08) |  | .03† |  |  | 0.10 (0.08) |  | .03 |  |  | 0.10 (0.08) |  | .03 | .02 |
| Separated |  | 0.10 (0.13) |  | .01 |  |  | 0.09 (0.13) |  | .01 |  |  | 0.08 (0.13) |  | .01 | .01 |
| Widowed |  | 0.17 (0.27) |  | .01 |  |  | 0.18 (0.27) |  | .01 |  |  | 0.18 (0.27) |  | .01 | .01 |
| Student |  | 0.19 (0.07) |  | .06** |  |  | 0.19 (0.07) |  | .06** |  |  | 0.18 (0.07) |  | .05** | .05 |
| Employed |  | 0.17 (0.08) |  | .05* |  |  | 0.17 (0.08) |  | .05* |  |  | 0.17 (0.08) |  | .05* | .04 |
| Retired or not seeking employment |  | 0.27 (0.11) |  | .06* |  |  | 0.28 (0.11) |  | .06* |  |  | 0.29 (0.11) |  | .06** | .05 |
| log Liquid wealth |  | 0.07 (0.06) |  | .03 |  |  | 0.04 (0.06) |  | .01 |  |  | 0.04 (0.06) |  | .01 | .01 |
| log Income |  | 0.03 (0.03) |  | .02 |  |  | 0.02 (0.03) |  | .02 |  |  | 0.02 (0.03) |  | .02 | .01 |
| log Investments |  | 0.01 (0.02) |  | .01 |  |  | 0.01 (0.02) |  | .01 |  |  | 0.01 (0.02) |  | .01 | .01 |
| Debt status (1 = has debt) | - | 0.08 (0.06) | - | .03 |  | - | 0.09 (0.06) | - | .03 |  | - | 0.10 (0.06) | - | .03 | .03 |
| log Utilitarian spending (exact) |  | 0.09 (0.05) |  | .04† |  |  | 0.02 (0.05) |  | .01 |  |  | 0.02 (0.05) |  | .01 | .01 |
| Utilitarian variety | - | 0.07 (0.05) | - | .03 |  | - | 0.11 (0.05) | - | .05* |  | - | 0.12 (0.05) | - | .05* | .04 |
| log Hedonic spending (exact) |  |  |  |  |  |  | 0.16 (0.05) |  | .08*** |  |  | 0.15 (0.05) |  | .08** | .05 |
| Hedonic variety |  |  |  |  |  |  |  |  |  |  |  | 0.03 (0.05) |  | .02 | .01 |
| *R*^2^ (adjusted *R*^2^) |  | .78 (.77) | | |  |  | .78 (.78) | | |  |  | .78 (.78) | | |  |
| Δ*R*^2^ |  | .001 | | |  |  | .004 | | |  |  | .000 | | |  |
| Model *F* (*df*_numerator_, *df*_denominator_) |  | 123.27 (20, 697) | | |  |  | 120.05 (21, 696) | | |  |  | 114.52 (22, 695) | | |  |
| *F*-change (*df*_numerator_, *df*_denominator_) |  | 2.17 (2, 697) | | |  |  | 13.05 (1, 696) | | |  |  | 0.41 (1, 695) | | |  |
| *Note*. **p* < .05. ***p* < .01. ****p* < .001.  Nominal demographic variables (e.g., employment) are dummy coded. Missingness dummy variables are not reported. | | | | | | | | | | | | | | | |

| Table S36  *Regression Models for Spending and Spending Variety Predicting Life Satisfaction (Sample, Time 2 Controlling for Time 1; Self-Reported Hedonic/Utilitarian Categories, Excluding Equally Hedonic/Utilitarian Categories)* | | | | | | | | | | | | | | | |
| --- | --- | --- | --- | --- | --- | --- | --- | --- | --- | --- | --- | --- | --- | --- | --- |
|  |  | Model 1: Utilitarian spending and variety | | |  |  | Model 2: Hedonic spending | | |  |  | Model 3: Hedonic variety | | | |
| Predictor |  | *B* (*SE*) |  | β |  |  | *B* (*SE*) |  | β |  |  | *B* (*SE*) |  | β | *r*_semipartial_ |
| Intercept |  | 3.40 (0.23) |  | .00*** |  |  | 3.31 (0.24) |  | .00*** |  |  | 3.32 (0.24) |  | .00*** |  |
| Time 1 Life satisfaction (centered) |  | 0.83 (0.02) |  | .82*** |  |  | 0.82 (0.02) |  | .82*** |  |  | 0.82 (0.02) |  | .82*** | .73 |
| Age | - | 0.00 (0.00) | - | .04 |  | - | 0.00 (0.00) | - | .04 |  | - | 0.00 (0.00) | - | .03 | .02 |
| Female |  | 0.01 (0.06) |  | .00 |  |  | 0.00 (0.06) |  | .00 |  |  | 0.00 (0.06) |  | .00 | .00 |
| Married |  | 0.28 (0.08) |  | .08*** |  |  | 0.27 (0.08) |  | .08*** |  |  | 0.28 (0.08) |  | .08*** | .06 |
| In relationship, unmarried |  | 0.13 (0.08) |  | .03 |  |  | 0.12 (0.08) |  | .03 |  |  | 0.12 (0.08) |  | .03 | .03 |
| Separated |  | 0.09 (0.13) |  | .01 |  |  | 0.10 (0.13) |  | .02 |  |  | 0.09 (0.13) |  | .01 | .01 |
| Widowed |  | 0.15 (0.27) |  | .01 |  |  | 0.14 (0.27) |  | .01 |  |  | 0.14 (0.27) |  | .01 | .01 |
| Student |  | 0.20 (0.07) |  | .06** |  |  | 0.20 (0.07) |  | .06** |  |  | 0.19 (0.07) |  | .06** | .05 |
| Employed |  | 0.17 (0.08) |  | .05* |  |  | 0.17 (0.08) |  | .05* |  |  | 0.17 (0.08) |  | .05* | .04 |
| Retired or not seeking employment |  | 0.28 (0.11) |  | .06* |  |  | 0.30 (0.11) |  | .06** |  |  | 0.30 (0.11) |  | .06** | .05 |
| log Liquid wealth |  | 0.07 (0.06) |  | .03 |  |  | 0.07 (0.06) |  | .03 |  |  | 0.07 (0.06) |  | .03 | .02 |
| log Income |  | 0.03 (0.03) |  | .02 |  |  | 0.03 (0.03) |  | .02 |  |  | 0.03 (0.03) |  | .02 | .02 |
| log Investments |  | 0.01 (0.02) |  | .02 |  |  | 0.01 (0.02) |  | .01 |  |  | 0.01 (0.02) |  | .01 | .01 |
| Debt status (1 = has debt) | - | 0.08 (0.06) | - | .03 |  | - | 0.08 (0.06) | - | .03 |  | - | 0.08 (0.06) | - | .03 | .03 |
| log Utilitarian spending (exact) |  | 0.03 (0.04) |  | .02 |  |  | 0.01 (0.04) |  | .01 |  |  | 0.01 (0.04) |  | .01 | .01 |
| Utilitarian variety | - | 0.01 (0.05) | - | .00 |  | - | 0.02 (0.05) | - | .01 |  | - | 0.03 (0.05) | - | .02 | .01 |
| log Hedonic spending (exact) |  |  |  |  |  |  | 0.07 (0.04) |  | .04† |  |  | 0.04 (0.05) |  | .02 | .02 |
| Hedonic variety |  |  |  |  |  |  |  |  |  |  |  | 0.06 (0.05) |  | .02 | .02 |
| *R*^2^ (adjusted *R*^2^) |  | .78 (.77) | | |  |  | .78 (.77) | | |  |  | .78 (.77) | | |  |
| Δ*R*^2^ |  | .000 | | |  |  | .001 | | |  |  | .000 | | |  |
| Model *F* (*df*_numerator_, *df*_denominator_) |  | 122.48 (20, 697) | | |  |  | 117.09 (21, 696) | | |  |  | 111.84 (22, 695) | | |  |
| *F*-change (*df*_numerator_, *df*_denominator_) |  | 0.42 (2, 697) | | |  |  | 2.82 (1, 696) | | |  |  | 1.13 (1, 695) | | |  |
| *Note*. **p* < .05. ***p* < .01. ****p* < .001.  Nominal demographic variables (e.g., employment) are dummy coded. Missingness dummy variables are not reported. | | | | | | | | | | | | | | | |

**Table S37. Recoded Open-ended Employment Responses in Study 1**

In Study 1, participants could provide open-ended responses to the employment question. These responses were recoded by one of the study authors into one of several categories (Employed, Student, Retired, or Unemployed). The table below lists the open-ended responses and the categories they were recoded into.

| **Open-ended response** | **PID** | **Recoded status** |
| --- | --- | --- |
| disabled with chronic pain | 25241249 | Unemployed |
| Self Employed | 25242363 | Employed |
| self employed | 25242895 | Employed |
| working long hours | 25267274 | Employed |
| Self employed | 25273822 | Employed |
| will be soon working student whilst compiling studies and work | 25279340 | Student |
| not working du to ill health | 25279476 | Unemployed |
| voluntary | 25285977 | Unemployed |
| Long term illness with some permitted work | 25296190 | Unemployed |
| apprentice | 25336016 | Student |
| Full time carer | 25337601 | Unemployed |
| applied for a job | 25340631 | Unemployed |
| full time carer | 25344645 | Unemployed |
| Full time student with government scholarship  and I have to work as a associate Prof. when after PhD | 25376928 | Student |
| self-employed, in and out of projects a lot, not a stable job. | 25382583 | Employed |
| carer | 25384599 | Unemployed |
| embassy job | 25405294 | Employed |
| currently on long term sick | 25415089 | Unemployed |
| locum agency worker | 25417535 | Employed |
| Maternity | 25418420 | Unemployed |
| Full time mother | 25424183 | Unemployed |
| Part-time | 25424676 | Unemployed |
| Setting up my own business | 25425538 | Unemployed |
| Supply teaching | 25456551 | Employed |
| On benefits due to disabilities | 25479143 | Unemployed |
| mother of 4 one disabled | 25480249 | Unemployed |
| disabled | 25480410 | Unemployed |
| Long term illness | 25481449 | Unemployed |
| Off work due to illness | 25483961 | Unemployed |
| medically retired | 25492452 | Unemployed |
| self-employed | 25497995 | Employed |
| working full time and studying | 25616026 | Employed |
